# The Khamkhains, Neurotrophic Drimane-Type Sesquiterpenoids Derived from a Polyporaceous Basidiomycete Originating from Thailand

**DOI:** 10.1021/acs.jnatprod.5c00669

**Published:** 2025-07-16

**Authors:** Pathompong Paomephan, Khadija Hassan, Marco Kirchenwitz, Sebastian Pfütze, Frank Surup, Ivana Císařová, Chuenchit Boonchird, Marc Stadler

**Affiliations:** † Department of Biotechnology, Faculty of Science, 98842 Mahidol University, Bangkok 10400, Thailand; ‡ Department of Microbial Drugs, Helmholtz Centre for Infection Research (HZI), Inhoffenstrasse 7, 38124 Braunschweig, Germany; § Institute of Microbiology, Technische Universität Braunschweig, Spielmannstraße 7, 38106 Braunschweig, Germany; ⊥ Department of Cell Biology, Helmholtz Centre for Infection Research, Inhoffenstrasse 7, 38124 Braunschweig, Germany; ∥ Department of Inorganic Chemistry, 37740 Charles University, Hlavova 2030/8, CZ-128 00 Prague 2, Czech Republic

## Abstract

Eight unprecedented terpenoids were isolated from submerged cultures of a polyporoid basidiomycete originating from Thailand (which had been referred to as “*Cerrena* sp.” in a previous publication) by preparative chromatography. Their chemical structures were elucidated by extensive two-dimensional nuclear magnetic resonance (NMR) spectroscopy and high-resolution mass spectrometry. One of the compounds was crystallized, and its absolute configuration was established by X-ray crystallography. Among the isolated metabolites were several members of the rare nitrogen-containing drimane type and one dimeric drimane, which consists of a nitrogen-containing monomer and a regular monomer. The latter compound represents a hitherto unknown type of terpenoid natural product. The metabolites were subjected to a biological characterization, and some of them showed significant neurotrophic effects. Notably, several of the compounds significantly enhanced the outgrowth of neurites in PC12 cells when treated with 5 ng/mL nerve growth factor. On the other hand, they were devoid of significant cytotoxic and antimicrobial effects.

## Graphical abstract

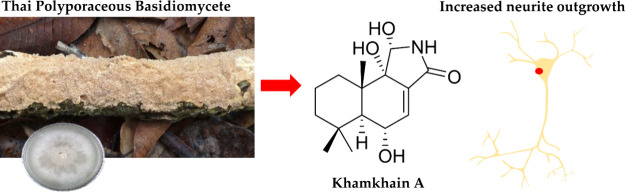

The Basidiomycota, and in particular their tropical species, constitute a rich source of bioactive metabolites with unique chemical scaffolds.^1,2^ Over the past few years, we have systematically explored these organisms for the production of novel antibiotics and other useful metabolites. Recently, we published findings on novel drimane derivatives from strains derived from Kenya and Thailand that exhibited moderate antimicrobial and cytotoxic effects.^3^ In the crude extracts of one of these strains, we also detected further secondary metabolites that appeared interesting from the HPLC-DAD/MS data. The current paper is dedicated to describing their isolation and structure elucidation as well as the biological characterization of several metabolites from cultures of a fungus collected in Thailand that was previously referred to as a “*Cerrena* sp”. We also provide some new evidence on the taxonomic position of the strain according to a comparison of its sequence data with new reference data that meanwhile became available from a study on Chinese polypores.^4^ By investigating the neurotrophic activities of drimane-type sesquiterpenoids, this research aims to expand the understanding of fungal bioactive metabolites and their potential applications in developing innovative neuroprotective therapies. Ultimately, this work contributes to the growing field of mycopharmaceuticals, offering promising avenues for combating neurodegenerative disorders.

## Results and Discussion

### Fungal Material and Preparation of Crude Extracts

A preliminary taxonomic characterization of the fungus, its fermentation, and the preparation of crude extracts have been previously described.^3^ The compounds reported herein were isolated concurrently with the drimanes already described in the previous paper using the same methodology. The producer strain was previously referred to as “a new species of *Cerrena*”. However, we found a flaw in our previous work that needs to be corrected! Here, we update the taxonomy of the producing organism, in light of new evidence that came up since publication of our first paper,^3^ pointing toward the fungus belonging to a different genus. The ITS nRDNA of strain BCC 84628 (with GenBank acc. no. MW512503) was found in retrospective to possess only ca. 90% identity to the published sequences of other *Cerrena* spp., including some vouchers from South America that can be regarded as authentic, as they were deposited by specialists. Therefore, it is not clear if the Thai fungus belongs to that genus as previously postulated in our preceding paper.^3^ A sequence identity of 90% is normally regarded in other groups of fungi as proof that the respective fungus belongs to the same family or order. As many fungal genomes were recently shown to possess very high degrees of polymorphisms in their rDNA,^5,6^ this DNA locus should not be used for discrimination of species without other corroborating evidence. It was even found that the ITS rDNA copies in one and the same genome may have only 89% identity.^7^ On the other hand, we compared the data on strain BCC 84628 with the more recently published sequences in GenBank and found that the strain studied by Wang et al.,^4^ which was published two years ago, contained more similar sequences than those from *Cerrena*, and the ITS sequence of the type strain of the recently erected Chinese species *Megasporia sinuosa* (GenBank acc. no. NG243097) and other sequences derived from different vouchers of the same species have over 97% identity to that of strain BCC 84628. We still hesitate to assign the Thai strain to a known genus because this would require more detailed morphological studies. The strain is therefore referred to under its BCC number in this manuscript. It should also be mentioned that the strain, as well as the phylogenetically related genus *Megasporia*, belongs to the Polyporaceae (and not the Cerrenaceae as previously claimed^3^).

### Structure Elucidation of Drimane-Type Sesquiterpenoids

In this study, we report the characteristics of eight hitherto undescribed metabolites, **1**–**8**. Their structures (Figure 1) were elucidated by using HRESIMS and NMR data. Previously, we had reported the isolation of isodrimeniol (**9**), the 4-aminobutyl derivative of ugandensolide (**10**), the glycinyl derivative of deacetylugandensolide (**11**), and cryptoporic acid H (**12**).^8,9^

**Figure 1:**
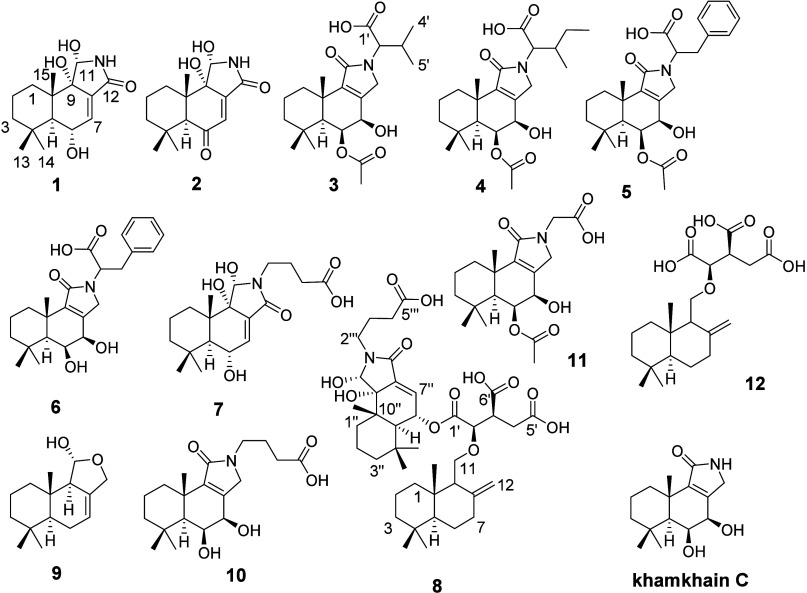
Structures of metabolites **1**–**12** isolated from fungal strain BCC 84628.

For metabolite **1** the quasimolecular ion peak at *m*/*z* 282.1698 in the HRESI spectrum indicated the molecular formula C_15_H_23_NO_4_. 1D and 2D NMR data revealed the drimane-type structure of **1** (see Figures S2–S6 for details). In comparison to isodrimeniol (**9**), a nitrogen atom replaced the oxygen in the five-membered ring system, and additional hydroxyl functions were present at C-6 and C-9. Furthermore, carboxyl C-12 replaced the methylene unit of isodrimeniol at this position. A strong ROESY correlation between methyl H_3_-15 and oxymethine H-6 indicated a pseudodiaxial orientation of these protons, and consequently a 6*R*′,10*S*′ configuration, while the large coupling constant *J*_H5,H6_ = 9.3 Hz afforded the 5*S′* configuration. The ROESY correlation between methyl H_3_-15 and oxymethine H-11 displayed an 11*S′* configuration. Finally, an X-ray analysis confirmed these assignments and established the absolute configuration of compound **1**. We suggest the trivial name khamkhain A for **1**. Since all of the congeners isolated are presumably biosynthesized by the same gene cluster, which is usually stereoselective, the X-ray data for **1** provided important information for interpretation of the absolute stereochemistry of the other compounds.

The molecular formula C_15_H_23_NO_4_ of **2** indicated the formal loss of 2*H* and thus an additional unsaturation compared to that of **1**. NMR data of compounds **1** and **2** were highly similar, with the replacement of oxymethine CH-6 in **1** by a keto function in **2** representing the difference. We suggest the name khamkhain B for **2**.

Molecular formulas of metabolites **3**–**5** were determined as C_22_H_33_NO_6_, C_23_H_35_NO_6_, and C_26_H_33_NO_6_, respectively, by HRESIMS data. Furthermore, 1D and 2D NMR data established core structures to be analogous to 6,7-dihydroxy-12-deoxy-dysidealactam, which we previously described.^3^ Differences from 6,7-dihydroxy-12-deoxy-dysidealactam are an additional acetyl group attached to C-6 and in the side chain attached to the nitrogen, being derived from valine, isoleucine, and phenylalanine, respectively. We suggest the name khamkhain C for 6,7-dihydroxy-12-deoxy-dysidealactam, and thus the names 6-acytyl-*N*-val-khamkhain C for **3**, 6-acytyl-*N*-isoleucyl-khamkhain C for **4**, and 6-acytyl-*N*-phe-khamkhain C for **5**.

*N*-Phe-khamkhain C (**6**) proved to be very similar to **5**, with the loss of the acetyl group attached to C-6 being the only difference.

NMR spectra of *N*-butanoyl-khamkhain A (**7**) were very similar to those of **1**, with the further signals of C-2′ to C-6′, indicating the presence of an additional butanoic acid residue.

The molecular formula of C_40_H_59_NO_12_ for compound **8** indicated a dimeric nature. The southern part of the molecule was identified as cryptoporic acid H (**12**), which had already been obtained in a previous study on the same strain.^3^ The northern part was established as matching compound **7**. Both hemispheres are connected by an ester bond linkage between C-6′′ and C-1′. We assigned the stereochemistry of **8** based on that of its building blocks.

Based on this naming system, **10** and **11** can be referred to as *N*-butanoyl-khamkhain C and *N*-Gly-6-acetyl-khamkhain C, respectively.

### Neurotrophic Activities

The potential of compounds **1**–**12** to induce rat pheochromocytoma (PC12) neuronal cell differentiation was analyzed. For PC12 and 1321N1 cell stimulation assays, nontoxic concentrations (10 μM) were used for this experiment. The chemically related 3β-hydroxy-9-dehydroxy-pereniporin A (**13**), 3β-hydroxy-6-*O*-acetylpereniporin A (**14**), pereniporin A (**15**), 6-*O*-acetylpereniporin A (**16**), 6α,9α,11α-trihydroxycinnamolide (**17**), and 11α-hydroxycinnamosmolide (**18**), which were isolated from *Perenniporia centrali-africana* and reported concurrently with compounds **13**–**15**,^3^ were tested in parallel.

PC12 cells did not exhibit cell differentiation upon compound addition (preliminary work; data not shown). However, using conditioned 1321N1 culture medium, PC12 cells could be stimulated to differentiate as compared to untreated controls, as shown in Figure 2. Compounds **1**, **5**, and **14** displayed the highest numbers of induced cell differentiation on PC12 cells compared to the negative control, with more than 50% at 68.0 ± 9.3%, 59.4 ± 3.3%, and 56.8 ± 6.1%, respectively. Interestingly, the compounds failed to stimulate differentiation of PC12 cells. A quantification of this assay is given in the Supporting Information (Table S3, Figure S53). These findings suggested that the three compounds induce higher levels of NGF expression compared to the other drimanes isolated in this study, which was further addressed by isolating mRNA from drimanes **1**-, **5**-, and **14**-treated 1321N1 astrocytes for quantitative RT-PCR. For control, expressed glyceraldehyde 3-phosphate dehydrogenase (GAPDH) served as reference for calibration. Coherent with the cell differentiation analysis, NGF expression was upregulated in 1321N1 astrocytes upon stimulation by compounds **1**, **5**, and **14** (Figure 3A), with **5** being the strongest NGF inducer (about 5-fold) followed by **14** resulting in an approximately 3-fold increase and **1** in a 2-fold increase in NGF-mRNA compared to DMSO-treated controls. In parallel, we performed quantitative RT-PCR analysis for mRNA expression of BDNF. Similarly, we observed an increase in BDNF mRNA levels for the compounds tested, as indicated in Figure 3B. The strongest BDNF inducer was **5**, then followed by **1** and last **13**. The endogenous mRNA level of BDNF in 1321N1 astrocytes seemed to be much higher than that of NGF for **1**, and the opposite was true for **5**, where a slight increase in NGF expression by about 3-fold and 6-fold was observed, respectively. In summary, three compounds from the polyporaceous Thai basidiomycete (i.e., **1**, **5**, and **13**) induced different patterns of neurotrophin expression in human astrocytes. For the first time, we observed a promoting effect of fungal drimane derivatives not just on NGF but also on BDNF expression. Thus, they represent interesting tools for the investigation of neurotrophic properties. Previously, the neurotrophic properties of triterpenes isolated from the basidiomycetes *Laetiporus sulphureus* and *Antrodia* sp. have been demonstrated through their potent stimulation of neurotrophic factor expression.^12^ Specifically, several compounds significantly enhanced the production of NGF, including sulphurenic acid, 15α-dehydroxytrametenolic acid, fomefficinic acid D, and 16α-hydroxyeburicoic acid. Additionally, both sulphurenic acid and 15α-dehydroxytrametenolic acid were found to promote BDNF expression. These findings highlight the potential of fungal-derived terpenes as bioactive compounds with neurotrophic effects, offering promising insights for neurodegenerative disease research.^12^ Another polyporoid basidiomycete, *Abundisporus violaceus*, yielded the drimanes abundisporins A–F, some of which also significantly enhanced neurite outgrowth when co-treated with 5 ng/mL NGF, highlighting the potential of this class for neurotrophic applications.^10^

**Figure 2:**
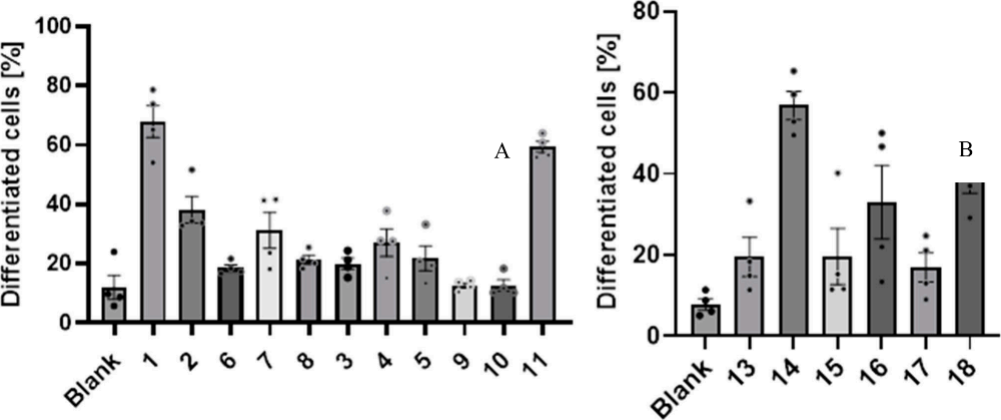
A quantification of analyses with the number of differentiated cells [%] of PC12 cells incubated with conditioned medium produced by 1321N1 cells treated with drimanes (A) **1**–**11** and (B) **13**–**18**; negative control (Blank). See also Table S3 for value numbers, Figure S53 for images of PC12 cells after incubation, and Figure S54 for structures of compounds **13**–**18**. Experiments were conducted using the same experimental setup as reported previously.^10,11^

**Figure 3:**
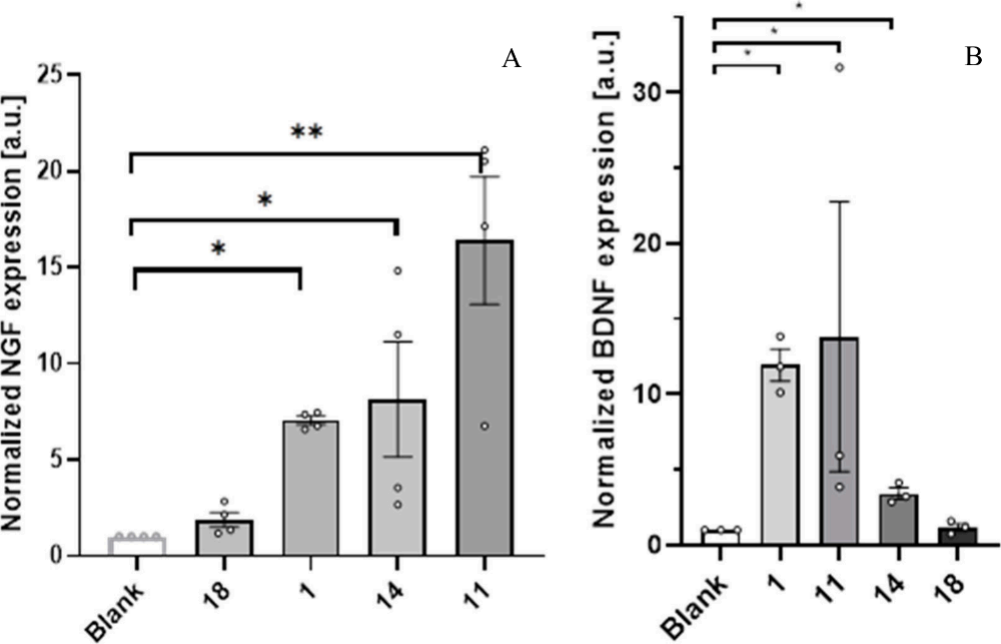
RT-PCR analysis for mRNA of (A) nerve growth factor (NGF) and (B) brain-derived neurotrophic factor (BDNF). (A) A significant increase of NGF was observed for all compounds tested except **18**. Similar results were observed in (B). Compounds **1** and **5** show a significantly higher BDNF mRNA amount when compared to the blank (±SEM; a, *p* < 0.05). See also Table S5. Experiments were conducted using the same experimental setup as reported previously.^10,11^

## Conclusion

The current study has identified several unprecedented neurotrophic terpenoids from cultures of Basidiomycota. A number of other studies on similar neutrotrophic effects were previously published in other genera of these fungi and their secondary metabolites. Examples include the cyathane terpenoids that are characteristic of the genera *Cyathus*, *Hericium*, and *Sarcodon*.^13–17^ Other drimane sesquiterpenoids have also been found to enhance nerve growth factor-mediated neurite outgrowth.^17^ The molecular targets of these compounds and the underlying biochemical phenomena still remain widely unknown. Based on the limited data available, it is not yet possible to infer structure–activity relations, which should best eventually be done by using semisynthesis, starting from one or more of the active molecules. However, for such a task, a scale-up of production would need to be carried out, which is beyond the scope of the current study. We plan to scale up and optimize fermentation of the fungus as a prerequisite for further work.

In any case, the current study once again has revealed that fungi and especially hitherto unexplored species can be a very rich source of unprecedented secondary metabolites. It is important to protect hitherto unexplored natural habitats and conduct some systematic forays there in order to isolate and preserve cultures of such organisms, because once the cultures are available, they can be examined and exploited in a straightforward manner as recently outlined by Schrey et al.^18^

## Experimental Section

### General Experimental Procedures

HPLC-DAD/MS measurements were performed using an amaZon speed ETD (electron transfer dissociation) ion trap mass spectrometer (Bruker Daltonics) and measured in positive and negative ion modes simultaneously, with the HPLC system (column C18 Acquity UPLC BEH (Waters), solvent A: water (H_2_O); solvent B: acetonitrile (ACN) supplemented with 0.1% formic acid (FA), gradient conditions: 5% B for 0.5 min, increasing to 100% B in 20 min, maintaining isocratic conditions at 100% B for 10 min, flow rate 0.6 mL/min). UV/vis detection (200–600 nm) was used.

HR-ESIMS (high-resolution electrospray ionization mass spectrometry) data were recorded on a MaXis ESI-TOF (electrospray ionization-time-of-flight) mass spectrometer (Bruker Daltonics, Bremen, Germany) coupled to an Agilent 1260 series HPLC-UV system and equipped with a C18 Acquity UPLC BEH (ethylene-bridged hybrid) (Waters) column; DAD-UV detection at 200–600 nm; solvent A (H_2_O) and solvent B (ACN) supplemented with 0.1% FA as a modifier; flow rate 0.6 mL/min, 40 C, gradient elution system with the initial condition 5% B for 0.5 min, increasing to 100% B in 19.5 min and holding at 100% B for 5 min. Bruker Compass DataAnalysis 4.4 SR1 was used to analyze the data, including determining the molecular formula using the Smart Formula algorithm (Bruker Daltonics). See Table S4 for retention times and other chromatography parameters.

1D and 2D NMR spectra were measured on a Bruker 700 MHz Avance III spectrometer equipped with a 5 mm TCI cryoprobe (^1^H: 700 MHz, ^13^C: 175 MHz) or a Bruker Avance III 500 (^1^H 500 MHz, ^13^C 125 MHz) spectrometer. NMR data were referenced to selected chemical shifts of acetone-*d*_6_ (^1^H: 2.05 ppm, ^13^C: 29.32 ppm) and DMSO-*d*_6_ (^1^H: 3.31 ppm, ^13^C: 49.15 ppm), respectively. Optical rotations were measured using an Anton Paar MCP-150 polarimeter (Graz, Austria) with a 100 mm path length and sodium D line at 589 nm. The spectral data are combined in the Supporting Information (Figures S3–S72).

### Fungal Material and Cultivation

The basidiomes of the producer strain were collected in Dong Yai Community Forest in the Plant Genetics Conservation Project, under the Royal Initiative of Her Royal Highness Maha Chakri Sirindhorn (RSPG), in Amnat Charoen Province, located in the northeastern part of Thailand, in March 2017. A voucher specimen is deposited in the BIOTEC Bangkok Herbarium & Fungarium, Pathum Thani, Thailand, with the designation BBH 41077, and the culture was deposited in the BIOTEC Culture Collection with the designation BCC 84628. The cultivation of the fungus on YM and BAF media was done as described in our previous paper, and the compounds described in the current study were obtained from the same crude extracts that had been prepared there.^3^

### Isolation of the Compounds from Strain BCC 84628

The isolation of the previously described drimanes **9**–**12** has been described in detail.^3^ Isolation of the eight further new derivatives **1**–**8** was performed analogously with the same instrumentation and HPLC programs. Utilized gradients, retention times, and yields for the individual compounds are listed in Table S4.

#### Spectral Data (Figures S1–S52)

##### Khamkhain A (**1**):

colorless oil, [α]_25_^D^ = −19 (*c* = 0.4, methanol); ^1^H NMR (500 MHz, DMSO-*d*_6_): see Table 1; ^13^C NMR (125 MHz, DMSO-*d*_6_): see Table 2; HRESIMS: 282.1698 [M + H]^+^ (calcd for C_15_H_24_NO_4_ 282.1700).

**Table 1:** ^13^C NMR Data (125 MHz) of New Metabolites **1**–**7**

	**1** ^a^	**2** ^b^	**3** ^b^	**4** ^b^	**5** ^b^	**6** ^b^	**7** ^b^
1	31.6, CH_2_	31.1, CH_2_	37.7, CH_2_	37.1, CH_2_	37.1, CH_2_	37.1, CH_2_	32.4, CH_2_
2	18.0, CH_2_	17.5, CH_2_	19.4, CH_2_	18.8, CH_2_	18.8, CH_2_	18.9, CH_2_	18.7, CH_2_
3	43.1, CH_2_	43.2, CH_2_	44.2, CH_2_	43.6, CH_2_	43.5, CH_2_	43.7, CH_2_	43.7, CH_2_
4	32.8, C	32.4, C	34.1, C	33.5, C	33.4, C	33.6, C	33.4, C
5	48.7, CH	56.4, CH	49.7, CH	49.1, CH	49.0, CH	50.1, CH	49.8, CH
6	66.8, CH	200.4, C	73.8, CH	73.3, CH	73.2, CH	71.9, CH	68.0, CH
7	133.2, CH	125.8, CH	67.1, CH	67.2, CH	66.5, CH	70.1, CH	134.3, CH
8	134.6, C	149.6, C	147.2, C	146.7, C	146.4, C	147.2, C	134.5, C
9	74.8, C	75.5, C	142.3, C	141.7, C	141.9, C	142.0, C	74.1, C
10	41.7, C	45.6, C	36.8, C	36.2, C	36.2, C	36.0, C	42.6, C
11	76.2, CH	77.0, CH	170.8, C	170.1, C	170.1, C	170.6, C	80.5, CH
12	166.7, C	165.4, C	49.2, CH_2_	48.5, CH_2_	48.5, CH_2_	48.7, CH_2_	165.3, C
13	22.4, CH_3_	21.2, CH_3_	23.5, CH_3_	22.9, CH_3_	22.8, CH_3_	23.4, CH_3_	22.4, CH_3_
14	36.1, CH_3_	33.5, CH_3_	33.7, CH_3_	33.1, CH_3_	33.1, CH_3_	33.4, CH_3_	36.1, CH_3_
15	17.8, CH_3_	18.8, CH_3_	21.4, CH_3_	20.8, CH_3_	20.6, CH_3_	20.6, CH_3_	18.0, CH_3_
Ac1			170.6, C	169.0, C	169.9, C		
Ac2			21.5, CH_3_	20.9, CH_3_	20.8, CH_3_		
1′			172.4, C	172.0, C	172.0, C	172.2, C	
2′			60.8, CH	59.7, CH	55.0, CH	55.0, CH	39.2, CH_2_
3′			29.7, CH	35.2, CH	35.9, CH_2_	35.9, CH_2_	23.5, CH_2_
4′			19.6, CH_3_	25.6, CH_2_	137.9, C	138.0, C	31.2, CH_2_
5′			19.9, CH_3_	10.6, CH_3_	129.2, CH	129.1, CH	173.7, C
6′				15.5, CH_3_	128.7, CH	128.7, CH	
7′					126.8, CH	126.8, CH	

a Measured in DMSO-*d*_6_.

b In acetone-*d*_6_.

**Table 2:** ^1^H NMR Data (500 MHz) of New Metabolites **1**–**7**

	**1** ^a^	**2** ^b^	**3** ^b^	**4** ^b^	**5** ^b^	**6** ^b^	**7** ^b^
1	1.63, m	2.00, m	2.70, m	2.70, m	2.63, m	2.59, m	1.82, m
	1.18, m	1.42, m	1.12, td (13.2, 3.7)	1.12, m	1.10, m	1.04, m	1.34, m
2	1.51, m	1.65, m	1.80, m	1.80, m	1.77, m	1.78, m	1.62, m
	1.38, m	1.49, m	1.52, m	1.51, m	1.50, m	1.46, m	1.46, m
3	1.32, m	1.39, m	1.47, m	1.47, m	1.46, m	1.41, m	1.40, m
	1.17, m	1.23, m	1.29, m	1.29, m	1.28, m	1.25, m	1.29, m
5	1.95, d (9.3)	3.07, s	1.72, br s	1.71, d (1.1)	1.69, d (0.8)	1.46, m	2.14, d (9.5)
6	4.15, m		5.45, br s	5.45, m	5.40. br s	4.32. br s	4.34. dd (9.5,3.4)
	OH: 4.55, d (7.5)						
7	6.19, d (3.4)	6.09, s	4.15, br s	4.15, d (0.9)	4.04, d (1.4)	4.05, d (1.7)	6.39, d (3.4)
9	4.90, s						
11	5.07, d (8.4)	5.48, br s					5.17, s
	OH: 8.31, s	OH: 7.94, br s					
	NH: 5.79, d (8.4)						
12			4.32, d (19.1)	4.34, d (19.0)	4.16, d (18.6)	4.17, d (18.6)	
			3.90, d (19.1)	3.87, d (19.0)	3.83, d (18.6)	3.78, d (18.6)	
13	1.01, s	1.15, s	1.04, s	1.04, s	1.01, s	1.23, s	1.09, s
14	1.11, s	1.17, s	1.01, s	1.00, s	0.98, s	0.98, s	1.20, s
15	0.79, s	1.05, s	1.50, s	1.49, s	1.35, s	1.40, s	0.92, s
Ac2			2.00, s	2.01, s	1.98, s		
1′							
2′			4.46, d (9.8)	4.57, d (9.7)	5.09, dd (10.5,5.5)	5.07, dd (10.4,5.5)	3.35, m
3′			2.28, m	2.05, m	3.38, dd (14.5,5.5)	3.37, dd (14.5,5.5)	1.87, m
					3.16, dd (14.5,10.5)	3.16, m	
4′			0.87, d (6.7)	1.39, m			2.30, br t (7.5)
				1.10, m			
5′			1.03, d (6.7)	0.89, t (7.5)	7.26, m	7.26, m	
6′				1.00, m	7.26, m	7.25, m	
7′					7.19, m	7.17, m	

a Measured in DMSO-*d*_6_.

b In acetone-*d*_6_.

##### Khamkhain B (**2**):

colorless oil, [α]_25_^D^ = – 21 (*c* = 0.94, methanol); ^1^H NMR (500 MHz, aceton-*d*_6_): see Table 1; ^13^C NMR (125 MHz, acetone-*d*_6_): see Table 2; HRESIMS: 280.1543 [M + H]^+^ (calcd for C_15_H_22_NO_4_ 280.1543).

##### 6-Acytyl-*N*-val-khamkhain C (**3**):

[α]_25_^D^ = +1 (*c* = 0.54, methanol); ^1^H NMR (500 MHz, aceton-*d*_6_): see Table 1; ^13^C NMR (125 MHz, acetone-*d*_6_): see Table 2; HRESIMS: 408.2377 [M + H]^+^ (calcd for C_22_H_34_NO_6_ 408.2381).

##### 6-Acytyl-*N*-isoleucyl-khamkhain C (**4**):

[α]_25_^D^ = +13 (*c* = 0.17, methanol); ^1^H NMR (500 MHz, aceton-*d*_6_): see Table 1; ^13^C NMR (125 MHz, acetone-*d*_6_): see Table 2; HRESIMS: 422.2540 [M + H]^+^ (calcd for C_23_H_36_NO_6_ 422.2537).

##### 6-Acytyl-*N*-phe-khamkhain C (**5**):

[α]_25_^D^ = +21 (*c* = 0.34, methanol); ^1^H NMR (500 MHz, aceton-*d*_6_): see Table 1; ^13^C NMR (125 MHz, acetone-*d*_6_): see Table 2; HRESIMS: 478.2193 [M + Na]^+^ (calcd for C_26_H_33_NO_6_N 478.2200), 456.2379 [M + H]^+^ (calcd for C_26_H_34_NO_6_ 456.2381).

##### *N*-Phe-khamkhain C (**6**):

[α]_25_^D^ = +15 (*c* = 0.17, methanol); ^1^H NMR (500 MHz, aceton-*d*_6_): see Table 1; ^13^C NMR (125 MHz, acetone-*d*_6_): see Table 2; HRESIMS: 436.2090 [M + Na]^+^ (calcd for C_24_H_31_NO_5_Na 436.2094), 414.2275 [M + H]^+^ (calcd for C_24_H_32_NO_5_ 414.2275).

##### *N*-butanoyl-khamkhain A (**7**):

[α]_25_^D^ = +7.5 (*c* = 0.3, methanol); ^1^H NMR (500 MHz, acetone-*d*_6_): see Table 1; ^13^C NMR (125 MHz, aceton-*d*_6_): see Table 2; HRESIMS: 368.2069 [M + H]^+^ (calcd for C_19_H_30_NO_6_ 369.2068).

##### Metabolite **8**:

[α]_25_^D^ = +31 (*c* = 0.17, methanol); ^1^H NMR (700 MHz, acetone-*d*_6_): δ_H_ 6.41 (d, *J* = 3.5 Hz, H-7″), 5.74 (dd, *J* = 9.6, 3.4 Hz, H-6″), 5.23 (s, H-11′′), 4.90 (br s, H-12a), 4.85 (m, H-12b), 4.19 (d, *J* = 3.7 Hz, H-2′), 4.01 (dd, *J* = 10.0, 8.4 Hz, H-11a), 3.62 (dd, *J* = 10.0, 3.6 Hz, H-11b),3.36 – 3.40 (m, H-1a, H-3′,H-2‴), 2.83 (dd, *J* = 17.2, 8.8 Hz, H-4′a), 2.60 (dd, *J* = 17.2, 5.2 Hz, H-4′b), 2.52 (br d, *J* = 9.8 Hz, H-5″), 2.38 (m, H-7a), 2.30 (br t, *J* = 7.5 Hz, H_2_-4), 2.05 (m, H-7b), 2.00 (m, H-9), 1.88 (m, H-1″a), 1.88 (m, H_2_-3‴), 1.76 (m, H-1a), 1.72 (m, H-6a), 1.66 (m, H-2a), 1.59 (m, H-2″a), 1.51 (m, H-2b), 1.46 (m, H-2″b), 1.37–1.44 (m, H-3a, H-3″a, H-1″b), 1.32 (m, H-3″b)), 1.33 (m, H-6b), 1.22 (m, H-3b), 1.21 (m, H-1b), 1.16 (m, H-5), 1.09 (s, H_3_-13″), 1.01 (s, H_3_-14″), 0.99 (s, H_3_-15″), 0.89 (s, H_3_-13), 0.83 (s, H_3_-14), 0.75 (s, H_3_-15) ppm; ^13^C NMR (175 MHz, acetone-*d*_6_): δ_C_ 174.5 (C, C-5‴), 173.5 (C, C-5′), 172.3 (C, C-6′), 171.0 (C, C-1′), 165.3 (C, C-12″), 147.8 (C, C-8), 138.3 (C-8″), 128.5 (CH-7″), 108.8 (CH_2_, C-12), 81.2 (CH, C-11″), 79.6 (CH, C-2′), 74.3 (C, C-9″), 71.9 (CH, C-6″), 68.7 (CH_2_, C-11), 56.3 (CH, C-9), 55.8 (CH, C-5), 47.5 (CH, C-5″), 44.9 (CH, C-3′), 43.6 (CH_2_, C-3″), 43.5 (C, C-10″), 42.8 (CH_2_, C-3), 39.95 (CH_2_, C-1), 39.93 (CH_2_, C-2‴), 39.4 (C, C-10), 38.3 (CH_2_, C-7), 35.8 (CH_3_, C-14″), 34.1 (C, C-4),34.0 (CH_3_, C-14) 33.8 (C, C-4″), 32.8 (CH_2_, C-4′), 32.6 (CH_2_, C-1″), 31.9 (CH_2_. C-4‴), 24.6 (CH_2_, C-6), 24.1 (CH_2_, C-3‴), 22.9 (CH_3_, C-13″), 22.1 (CH_3_, C-13), 19.9 (CH_2_, C-2″), 19.0 (CH_2_, C-2), 18.5 (CH_3_, C-15″), 15.7 (CH_3_, C-15) ppm; HRESIMS: 746.4109 [M + H]^+^ (calcd for C_40_H_60_NO_12_ 746.4110).

### X-ray Analysis of Khamkain A (**1**)

The X-ray experimental data for **1** were acquired on a Bruker D8 VENTURE Kappa Duo PHOTON III instrument by an IμS microfocus sealed tube with CuKα (λ = 1.54178 Å) radiation at the temperature of 120 K. The structures were solved by direct methods (XT) and refined by full matrix least-squares based on *F* ^2^ (SHELXL2019).^19^ The hydrogen atoms on carbon were fixed into idealized positions (riding model) and assigned temperature factors of either H_iso_(H) = 1.2 *U*_eq_(pivot atom) or H_iso_(H) = 1.5 *U*_eq_ (pivot atom) for the methyl moieties. The hydrogens on N and O atoms were found on difference Fourier maps and refined under a rigid-body assumption. The unit cell contains a disordered water molecule, where the oxygen atom is split into two positions, whereas the hydrogens are involved in a three-dimensional network of hydrogen bonds formed by the −O–H and −N–H moieties of **1** (Tables S1, S2). The determination of the absolute configuration was based on anomalous scattering of oxygen and nitrogen atoms.^20^

Crystal data for **1**: C_15_H_23_NO_4_)·0.5­(H_2_O), *M*_r_ = 290.35, monoclinic, *P*21 (No. 4), *a* = 8.1520(3) Å, *b* = 6.4219(3) Å, *c* = 14.4022(5) Å, β = 102.014(3)°, *V* = 737.46(5) Å^3^, *Z* = 2, *D*_x_ = 1.308 Mg m^–3^, colorless bar of dimensions 0.51 × 0.05 × 0.03 mm, temperature of the crystal 120 K, multiscan absorption correction (μ = 0.79 mm^–1^) *T*_min_ = 0.688, *T*_max_ = 0.979; a total of 8572 measured reflections (θ_max_ = 72.2°), from which 2731 were unique (*R*_int_ = 0.034) and 2568 observed according to the *I* > 2σ­(*I*) criterion. The refinement converged (Δ/σ_max_ ≤ 0.001) to *R* = 0.035 for observed reflections and *wR*(*F* ^2^) = 0.089, GOF = 1.03 for 197 parameters and all 2731 reflections. The final difference map displayed no peaks of chemical significance (Δρ_max_ = 0.23, Δρ_min_ −0.17 e·Å^–3^). Absolute structure parameter: −0.06(13).

X-ray crystallographic data have been deposited with the Cambridge Crystallographic Data Centre (CCDC) under deposition number 2414590 for **1** and can be obtained free of charge from the Centre via its Web site https://www.ccdc.cam.ac.uk/structures/.

### Cytotoxicity Assay

*In vitro* cytotoxicity (IC_50_) assessments were carried out on the isolated compounds based on the MTT (3-(4,5-dimethylthiayol-2-yl)-2,5-diphenyltetrazolium bromide) test in 96-well plates, using the cell lines KB3.1 (human endocervical adenocarcinoma) and L929 (mouse fibroblasts), in accordance with our previously established methods.^10,12^ Epothilone B was used as a positive control. These experiments preceded the assays described below to ensure that the compounds were devoid of significant cytotoxic effects.

### Cell Culture for Neurotrophic Assays

These experiments were conducted using exactly the same methodology as recently reported.^11^ All compounds were first tested before for cytotoxicity as described above but were found to be devoid of significant effects up to 66.6 μg/mL. Briefly, Astrocytoma (1321N1, Sigma-Aldrich, acc. no. 86030402) cells were cultured in Gibco DMEM medium (Fisher Scientific, Inc., Waltham, MA, USA) containing 10% heat-inactivated FBS (Capricorn Scientific GmbH, Ebsdorfergrund, Germany). Rat pheochromocytoma cells (PC-12) purchased from the European Collection of Authenticated Cell Cultures (ECACC) general collection were grown in Gibco RPMI-1640 (Fisher Scientific) medium containing 10% horse serum (Capricorn) and 5% heat-inactivated fetal bovine serum-FBS (Capricorn). The media were supplemented with penicillin (0.15 mM), streptomycin (86 μM), and glutamine (2 mM). The cells were incubated at 37 °C in a humidified environment of 7.5% CO_2_ and 95% air and routinely passaged every 3–4 days. Collagen type IV (Sigma C5533) was coated on 96-well plates and left for 6 h or more before using the plates whenever seeding PC-12 cells.

### Neurite Outgrowth Assay

The course of screening was conducted using a neurite outgrowth assay as previously described.^11^ PC-12 cells were seeded at a density of 1 × 10^3^ cells per well in growth medium in 96-well culture plates and incubated overnight. The supernatant of treatment between compounds and 1321N1 cells was transferred to treat PC12 cells. Cells treated with nerve growth factor (50 ng/mL) were used as a positive control. After 3 days, cell differentiation and neurite outgrowth were examined using an IncuCyte S3 live-cell analysis system (Sartorius, Göttingen, Germany). Six random fields were examined in each well. Neurite length was measured using the IncuCyte NeuroTrack Software Module for 6 days. The number of cells differentiated, i.e., axon-like protusions in the cells, defined as extensions longer than twice the cell body diameter, was recorded. Three independent experiments were conducted for each compound.

### cDNA Synthesis and Real-Time Quantitative RT-PCR

The induction of neurotrophin expression in 1321N1 cells was tested as previously described, as PC12 cells do not produce NGF by their own.^11^ For real-time quantitative reverse transcriptase PCR, the total RNA was extracted from treatment of 1321N1 cells (2 × 10^5^ cells) with selected compounds. For treatment, culture media was replaced by serum-reduced medium (Gibco RPMI with 1% FBS (Capricorn)), and cells were incubated for 24 h. Then, media was replaced with media containing the drimanes dissolved in 0.5% DMSO. As control, serum-reduced medium supplemented with 0.5% DMSO was used. Cells were incubated for 48 h. Total RNA was extracted using the NucleoSpinR RNA Plus kit (Macherey-Nagel GmbH& Co KG, Düren, Germany) followed by further purification (NucleoSpinR RNA Clean-up kit) according to the manufacturer’s protocol. To determine the concentration of purified RNA, the corresponding samples were measured using a DS-11+ Nanodrop spectrophotometer (DeNovix Inc., Wilmington, DE, USA). First strand cDNA synthesis and subsequent real-time PCR were performed using SensiFast SYBR No-Rox one-step kit (Cat. No. BIO-72005 (Bioline)). The following PCR primers were used for amplifying specific cDNA fragments^11^ as shown in Table S5. The PCR reactions were performed in a 10 μL volume containing cDNA template (2 μL), SensiFast SYBR No-Rox one-step mix (5 μL), primers (400 nM; 0.4 μL), reverse transcriptase (0.1 μL), RiboSafe RNase inhibitor (0.2 μL), and Rnase free water (1.9 μL). The amplified cDNAs were analyzed and quantified using Qiagen (Corbett) Rotor-Gene 3000 and LightCyclerR 96 (Roche Diagnostics International Ltd., version 1.1.0.1320) real-time PCR instruments. Amounts of gapdh amplicons were used as reference and set as 1.

### Statistical Analysis

The data obtained from neurite outgrowth assays were analyzed on Prism V8 software (Graph Pad Software Inc.) by employing the Student *t*-test statistical method. Data are displayed as the mean ± SEM.

## Supplementary Material





## Notes

Figures S1–S52: Spectral data for compounds **1–12**; Table S1. Hydrogen-bond geometry for **1**; Table S2. Geometric parameters for **1**; Table S3. Result of a quantification assay; Figure S53. The image of PC12 cells after incubation with supernatant from astrocyte cells with various supplements at 5 days; Table S4. The gradients, retention times, and yields for the individual compounds; Table S5. PCR primers used for amplifying specific cDNA fragments; GenBank BLAST search for “*Cerrena* sp.” BCC 84628 performed on Jan 29, 2025 (PDF)
NMR raw data of compounds **1**–**8** (ZIP)
