# Supplementary material for: The Khamkhains, Neurotrophic Drimane-Type Sesquiterpenoids Derived from a Polyporaceous Basidiomycete Originating from Thailand
Source: J Nat Prod. 2025 Jul 16;88(7):1840–6. doi: 10.1021/acs.jnatprod.5c00669 (PMC12305638; doi:10.1021/acs.jnatprod.5c00669)
Supplement: Supplementary file 1 [file np5c00669_si_001.pdf]

The Khamkhains, Neurotrophic Drimane-type Sesquiterpenoids Derived From a Polyporaceous Basidiomycete Originating from Thailand

Pathompong Paomephan[†], Khadija Hassan[‡,§], Marco Kirchenwitz[‡,§], Sebastian Pfütze[‡,§], Frank Surup[‡,§], Ivana Císařová[‖], Chuenchit Boonchird*[,†] and Marc Stadler*[,‡,§]

† These authors contributed equally to this work

† Department of Biotechnology, Faculty of Science, Mahidol University, Bangkok 10400, Thailand

‡ Department of Microbial Drugs, Helmholtz Centre for Infection Research (HZI), Inhoffenstrasse 7, 38124 Braunschweig, Germany

§ Institute of Microbiology, Technische Universität Braunschweig, Spielmannstraße 7, 38106 Braunschweig, Germany

⊥ Department of Cell Biology, Helmholtz Centre for Infection Research, Inhoffenstrasse 7, 38124 Braunschweig, Germany

‖ Department of Inorganic Chemistry, Charles University, Hlavova 2030/8, CZ-128 00 Prague 2, Czech Republic

*** Corresponding Authors**

marc.Stadler@helmholtz-hzi.de (M.S.) Tel.: +49-531-6181-4240; and chuenchit.boo@mahidol.ac.th (C.B.); Tel.: +66-2-201-5304 (C.B)

(M.S.) Tel.: +49-531-6181-4240; and chuenchit.boo@mahidol.ac.th (C.B.); Tel.: +66-2-201-5304 (C.B)

Supplementary Information

Contents

Figure S1 HPLC-HRESIMS data of **1** 4

Figure S2 1H NMR spectrum (500 MHz, DMSO-d_6) of **1**. 5

Figure S3 13C NMR spectrum (125 MHz, DMSO-d_6) of **1**. 6

Figure S4 HSQC NMR spectrum (500 MHz, DMSO-d_6) of **1**. 7

Figure S5 COSY NMR spectrum (500 MHz, DMSO-d_6) of **1**. 8

Figure S6 HMBC NMR spectrum (500 MHz, DMSO-d_6) of **1**. 9

Figure S7 HPLC-HRESIMS data of **2**. 10

Figure S8 1H NMR spectrum (500 MHz, aceton-d_6) of **2**. 11

Figure S9 13C NMR spectrum (125 MHz, aceton-d_6) of **2**. 12

Figure S10 HSQC NMR spectrum (500 MHz, aceton-d_6) of **2**. 13

Figure S11 COSY NMR spectrum (500 MHz, aceton-d_6) of **2**. 14

Figure S12 HMBC NMR spectrum (500 MHz, aceton-d_6) of **2**. 15

Figure S13 ROESY NMR spectrum (500 MHz, aceton-d_6) of **2**. 16

Figure S14 HPLC-HRESIMS data of **3** 17

Figure S15 1H NMR spectrum (500 MHz, aceton-d_6) of **3**. 18

Figure S16 13C NMR spectrum (125 MHz, aceton-d_6) of **3**. 19

Figure S17 HSQC NMR spectrum (500 MHz, aceton-d_6) of **3**. 20

Figure S18 COSY NMR spectrum (500 MHz, aceton-d_6) of **3**. 21

Figure S19 HMBC NMR spectrum (500 MHz, aceton-d_6) of **3**. 22

Figure S20 HPLC-HRESIMS data of **4** 23

Figure S21 1H NMR spectrum (500 MHz, acetone-d_6) of **4** 24

Figure S22 13C NMR spectrum (125 MHz, acetone-d_6) of **4**........ 25

Figure S23 HSQC NMR spectrum (500 MHz, acetone-d_6) of **4** 26

Figure S24 COSY NMR spectrum (500 MHz, acetone-d_6) of **4** 27

Figure S25 HMBC NMR spectrum (500 MHz, acetone-d_6) of **4** 28

Figure S26 HPLC-HRESIMS data of **5**. 29

Figure S27 1H NMR spectrum (500 MHz, acetone-d_6) of **5** 30

Figure S28 13C NMR spectrum (125 MHz, acetone- d_6) of **5** 31

Figure S29 HSQC NMR spectrum (500 MHz, acetone-d_6) of **5** 32

Figure S30 COSY NMR spectrum (500 MHz, acetone-d_6) of **5** 33

Figure S31 HMBC NMR spectrum (500 MHz, acetone-d_6) of **5** 34

Figure S32 HPLC-HRESIMS data of **6**. 35

Figure S33 1H NMR spectrum (500 MHz, acetone-d_6) of **6** 36

Figure S34 13C NMR spectrum (125 MHz, acetone-d_6) of **6** 37

Figure S35 HSQC NMR spectrum (500 MHz, acetone-d_6) of **6** 38

Figure S36 COSY NMR spectrum (500 MHz, acetone-d_6) of **6**) 39

Figure S37 HMBC NMR spectrum (500 MHz, acetone-d_6) of **6** 40

Figure S38 HPLC-HRESIMS data of **7** 41

Figure S39 1H NMR spectrum (500 MHz, acetone-d_6) of **7** 42

Figure S40 13C NMR spectrum (125 MHz, acetone-d_6) of **7** 43

Figure S41 HSQC NMR spectrum (500 MHz, acetone-d_6) of **7** 44

Figure S42 COSY NMR spectrum (500 MHz, acetone-d_6) of **7** 45

Figure S43 HMBC NMR spectrum (500 MHz, acetone-d_6) of **7** 46

Figure S44 1H NMR spectrum (700 MHz, acetone-d_6) of **8** 47

Figure S45 13C NMR spectrum (175 MHz, acetone-d_6) of **8** 48

Figure S46 HSQC NMR spectrum (700 MHz, acetone-d_6) of **8**........ 49

Figure S47 COSY NMR spectrum (700 MHz, acetone-d_6) of **8**........ 50

Figure S48 HMBC NMR spectrum (700 MHz, acetone-d_6) of **8**........ 51

Figure S49 1H NMR spectrum (500 MHz, acetone-d6) of cryptoporic acid H (**12**). 52

Figure S50 13C NMR spectrum (125 MHz, acetone-d6) of cryptoporic acid H (**12**)........ 53

Figure S51 COSY (blue arrows), HMBC (green arrows) and ROESY (purple arrows) correlations for **1** and **2**. 54

Figure S52 COSY (blue arrows), HMBC (green arrows) and ROESY (purple arrows) correlations for **3**–**11** & **12**. 55

Table S1. Hydrogen-bond geometry (Å, º) for **1**. 56

Table S2. Geometric parameters (Å, º) for **1**. 56

Table S3 Result of a quantification assay 57

Figure S53 The image of PC12 cells after incubation with supernatant from astrocyte cells with various supplements at 5 days........ 58

Table S4 The gradients, retention times and yields for the individual compounds 59

Table S5 The following PCR primers were used for amplifying specific cDNA fragments........ 61

Method descriptions........ 62

GenBank BLAST search for “Cerrena sp.” BCC 84628 (with GenBank acc no. MW512503) performed on Nov 29,2024........ 63

Figure S54. Structures of metabolites **13** – **18** isolated from *Perenniporia centrali-africana* MUCL 56028.. 63

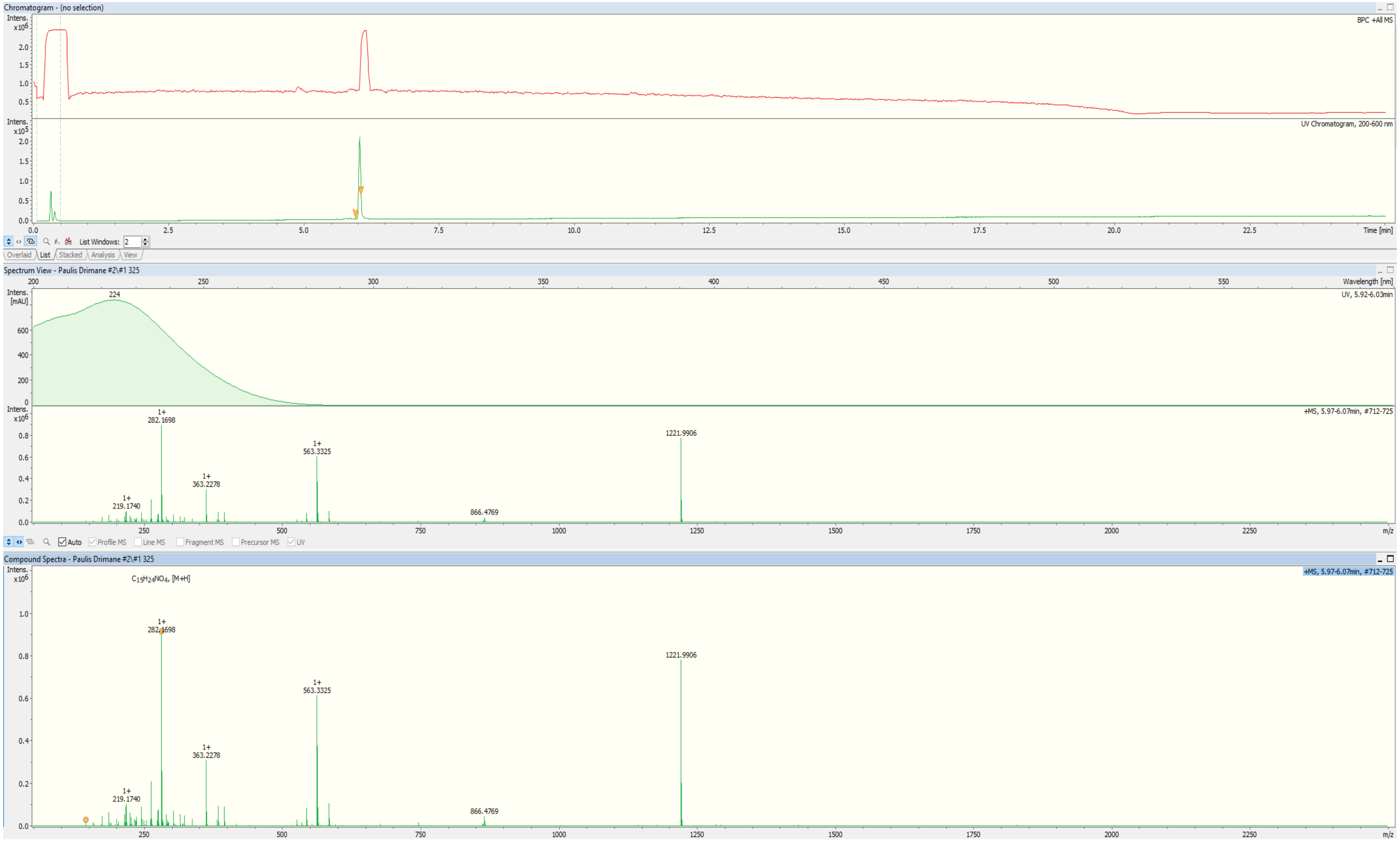

Figure S1 HPLC-HRESIMS data of **1**.

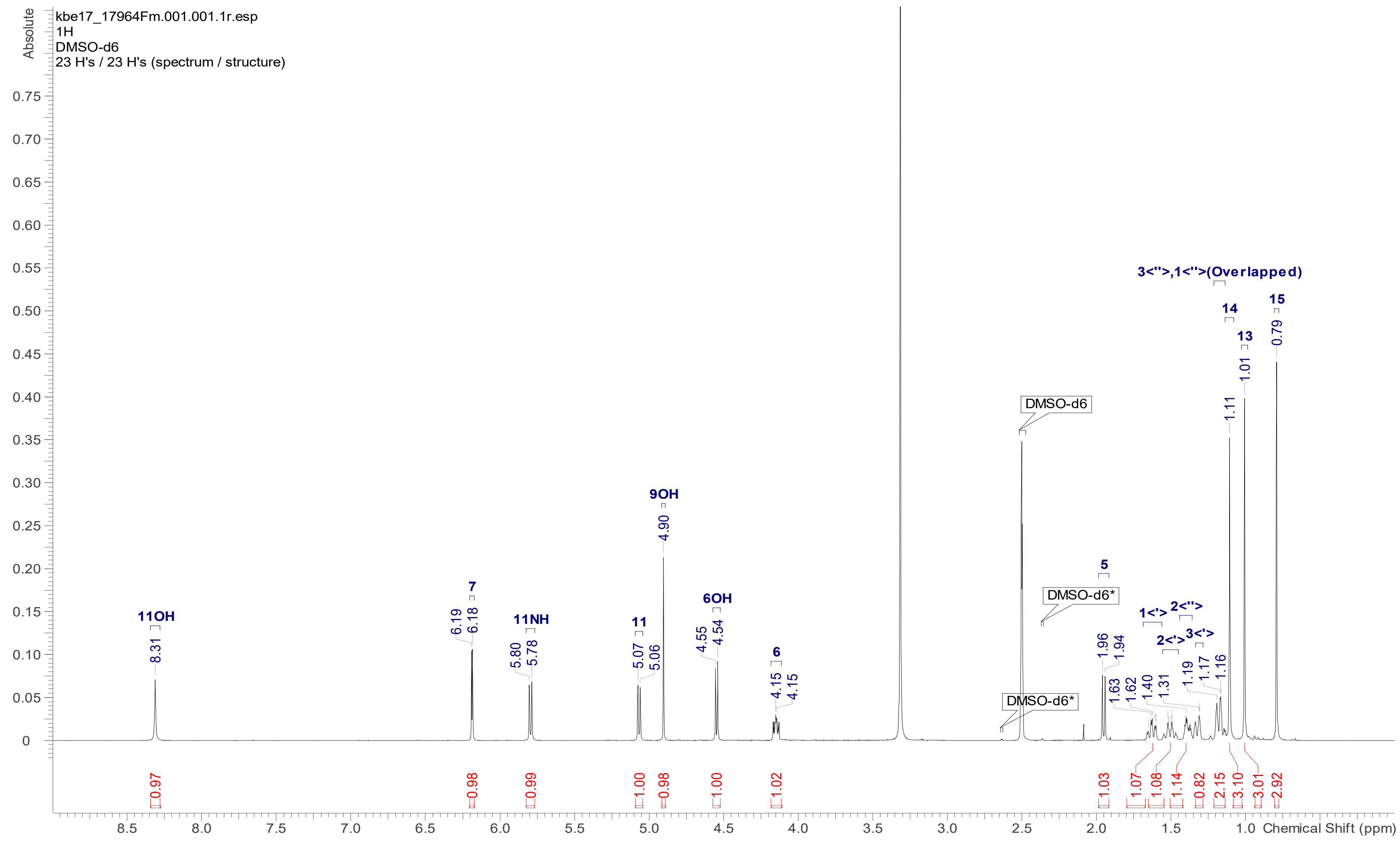

Figure S2 ^{1}H NMR spectrum (500 MHz, DMSO-d_6) of **1**.

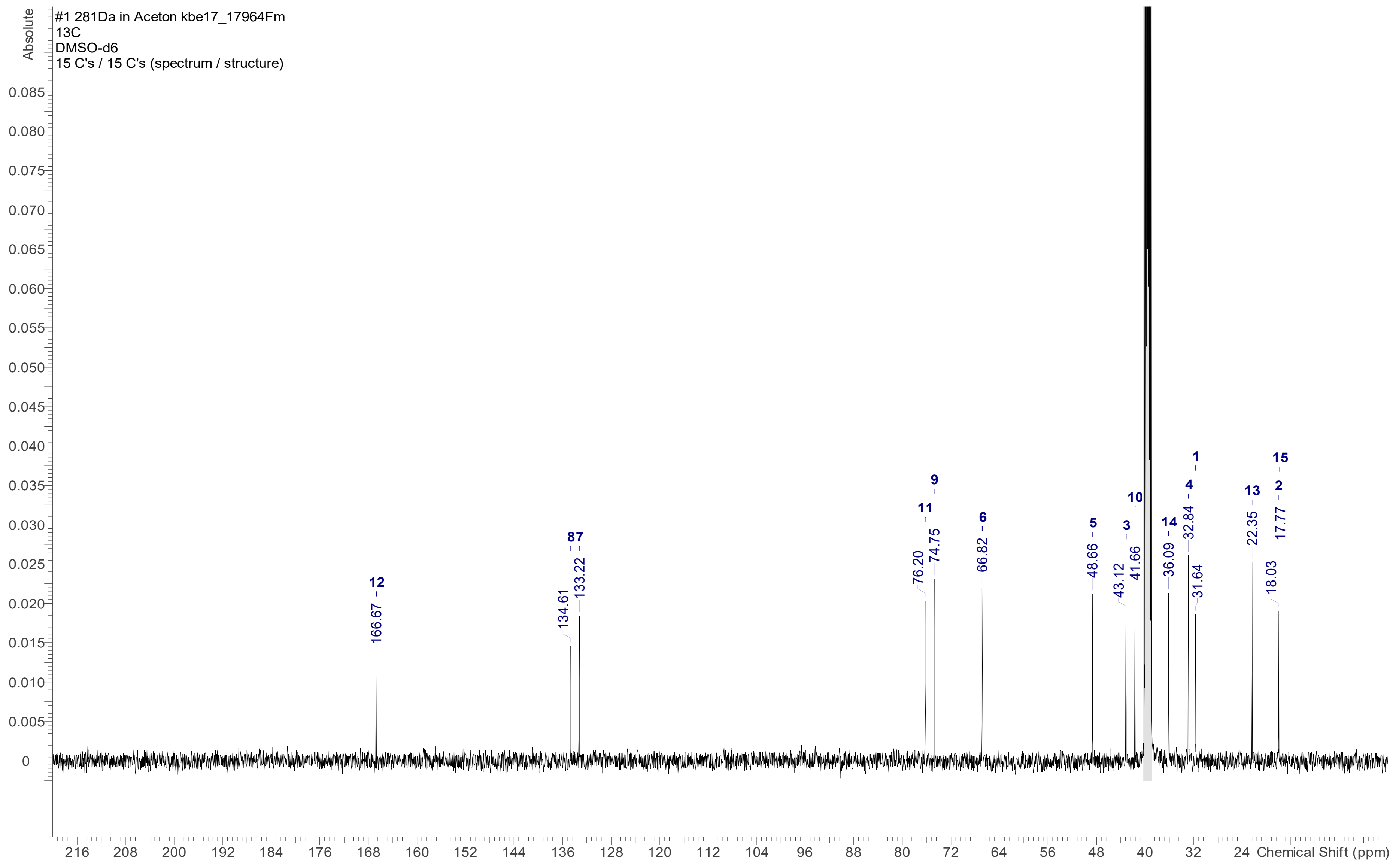

Figure S3 ^{13}C NMR spectrum (125 MHz, DMSO-d_6) of **1**.

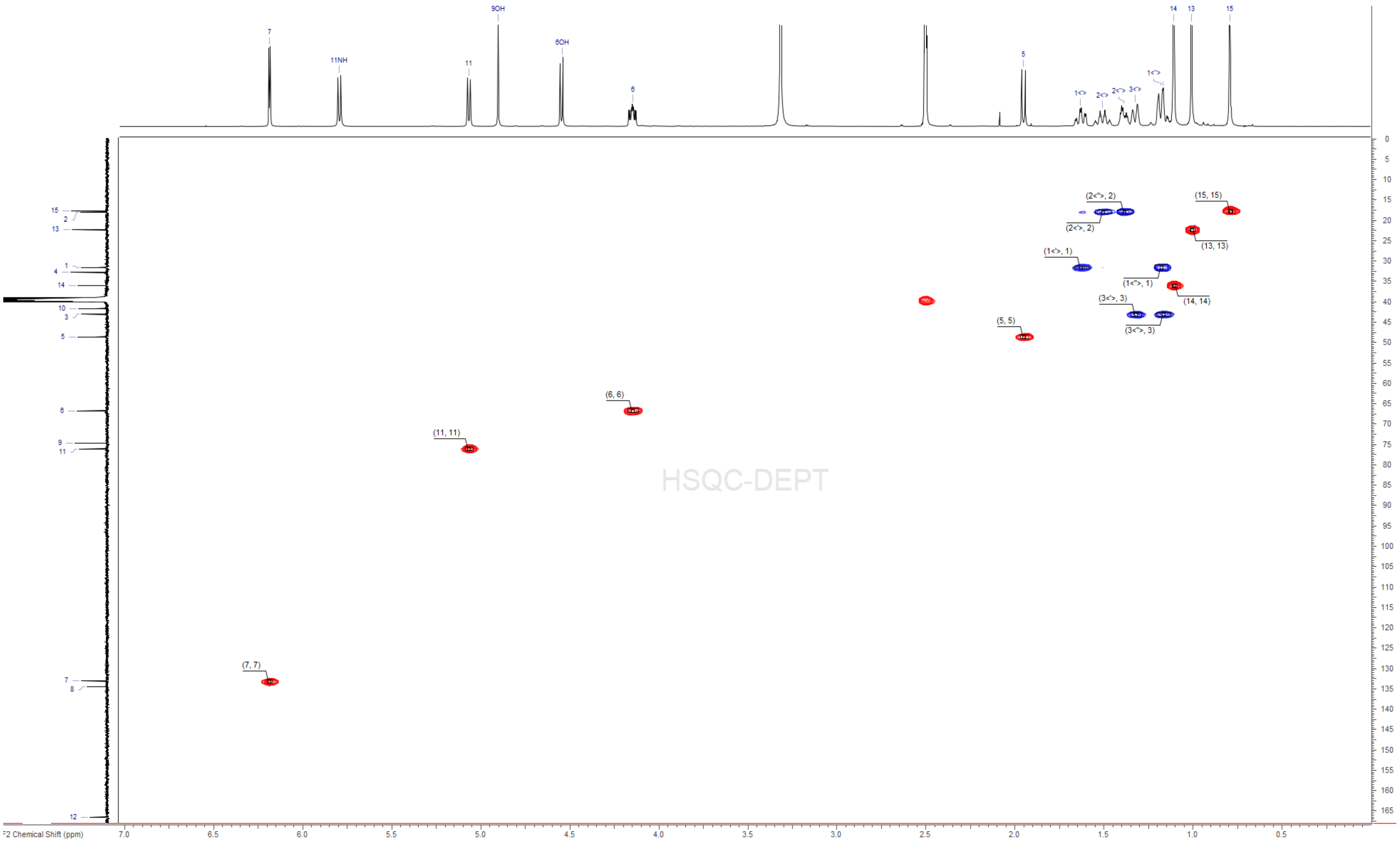

Figure S4 HSQC NMR spectrum (500 MHz, DMSO-d_6) of **1**.

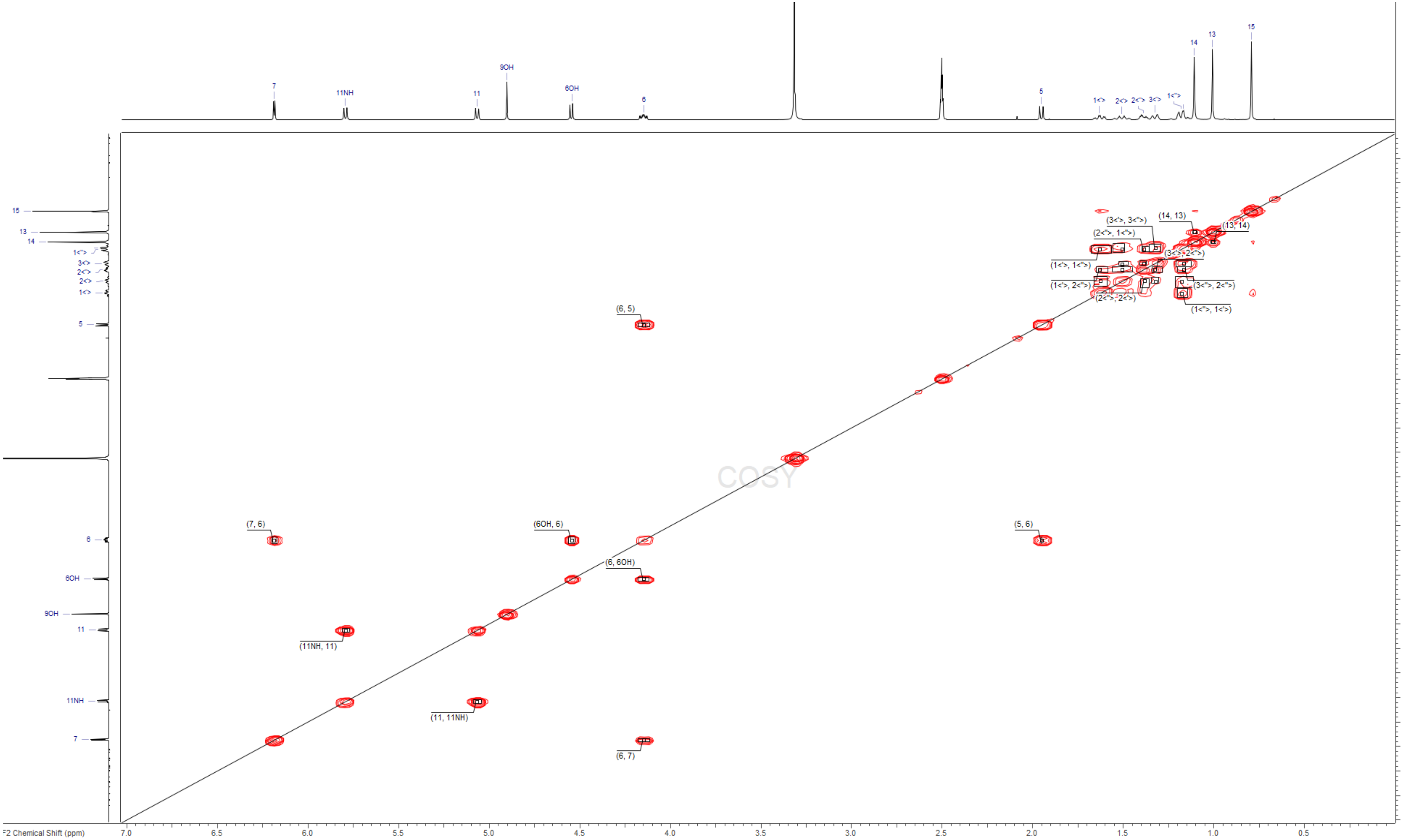

Figure S5 COSY NMR spectrum (500 MHz, DMSO-d_6) of **1**.

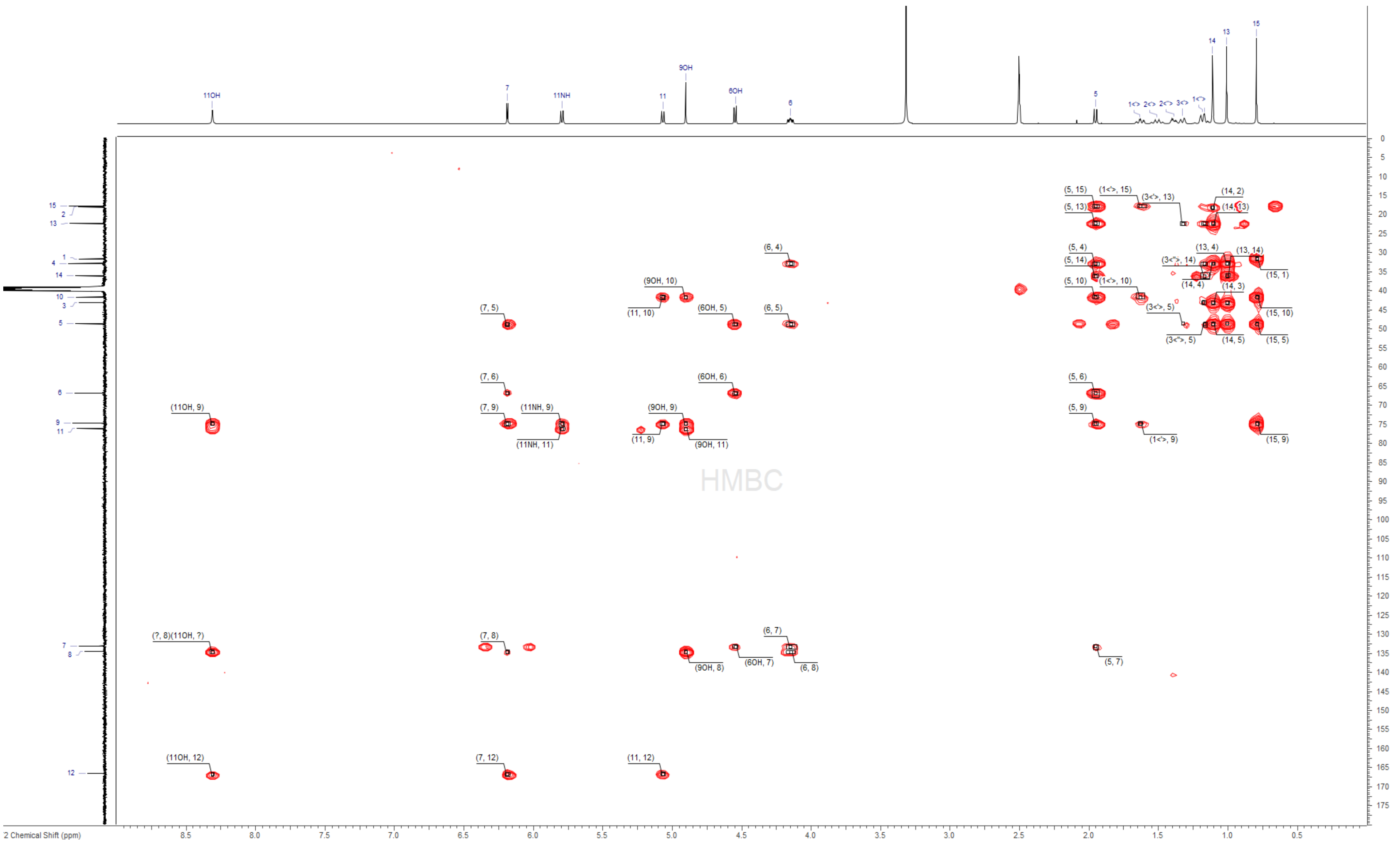

Figure S6 HMBC NMR spectrum (500 MHz, DMSO-d_6) of **1**.

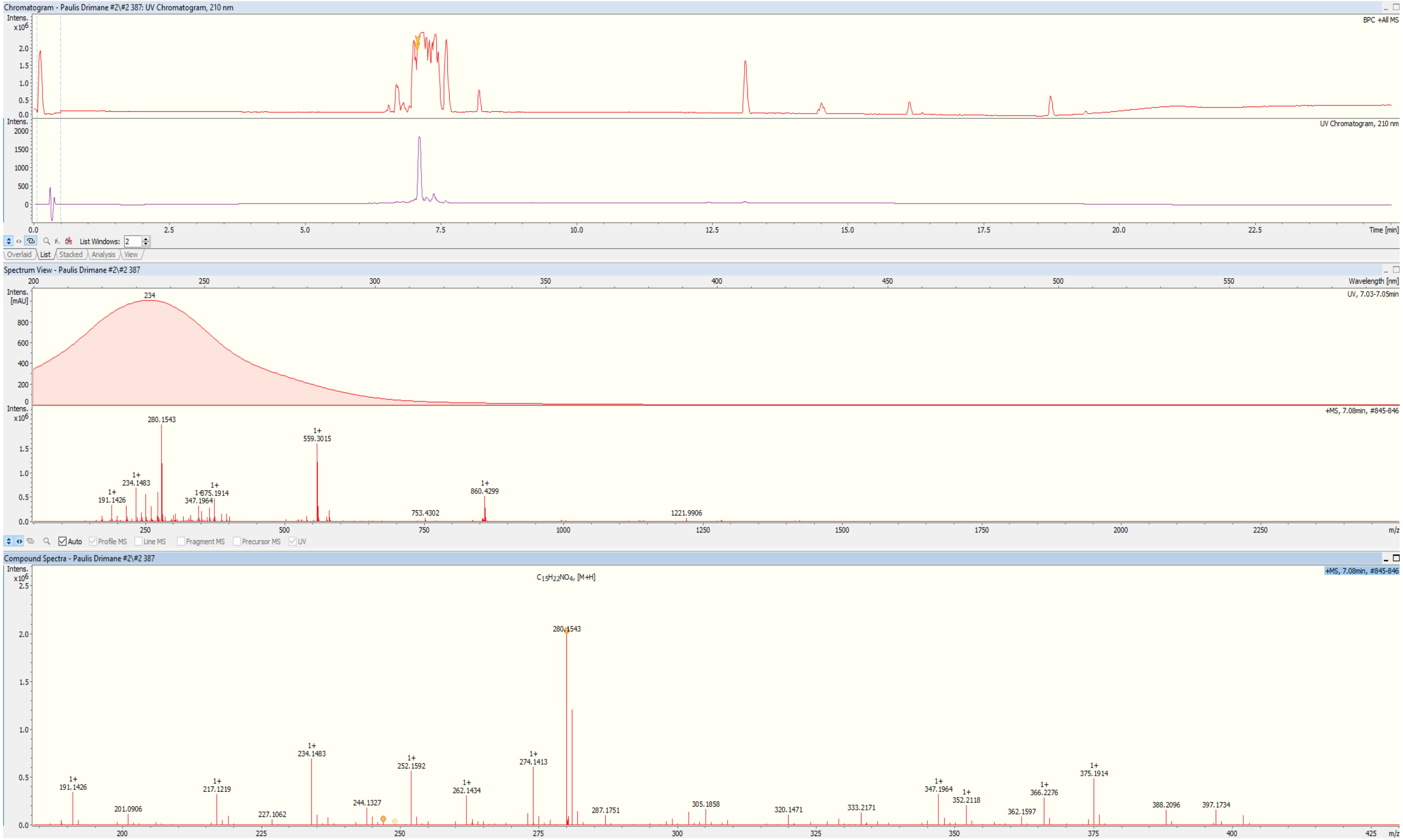

Figure S7 HPLC-HRESIMS data of **2**.

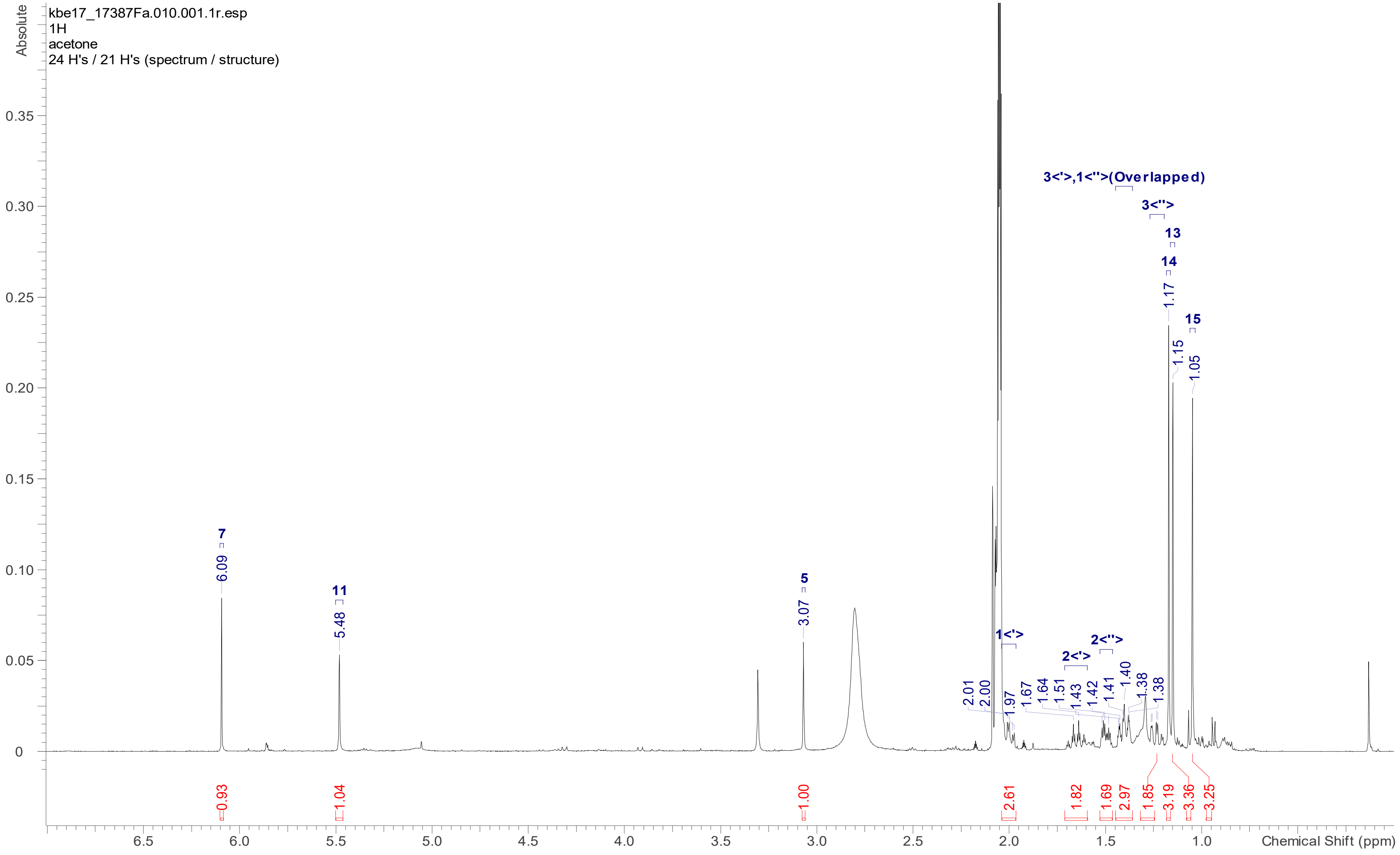

Figure S8 ^{1}H NMR spectrum (500 MHz, aceton-d_6) of **2**.

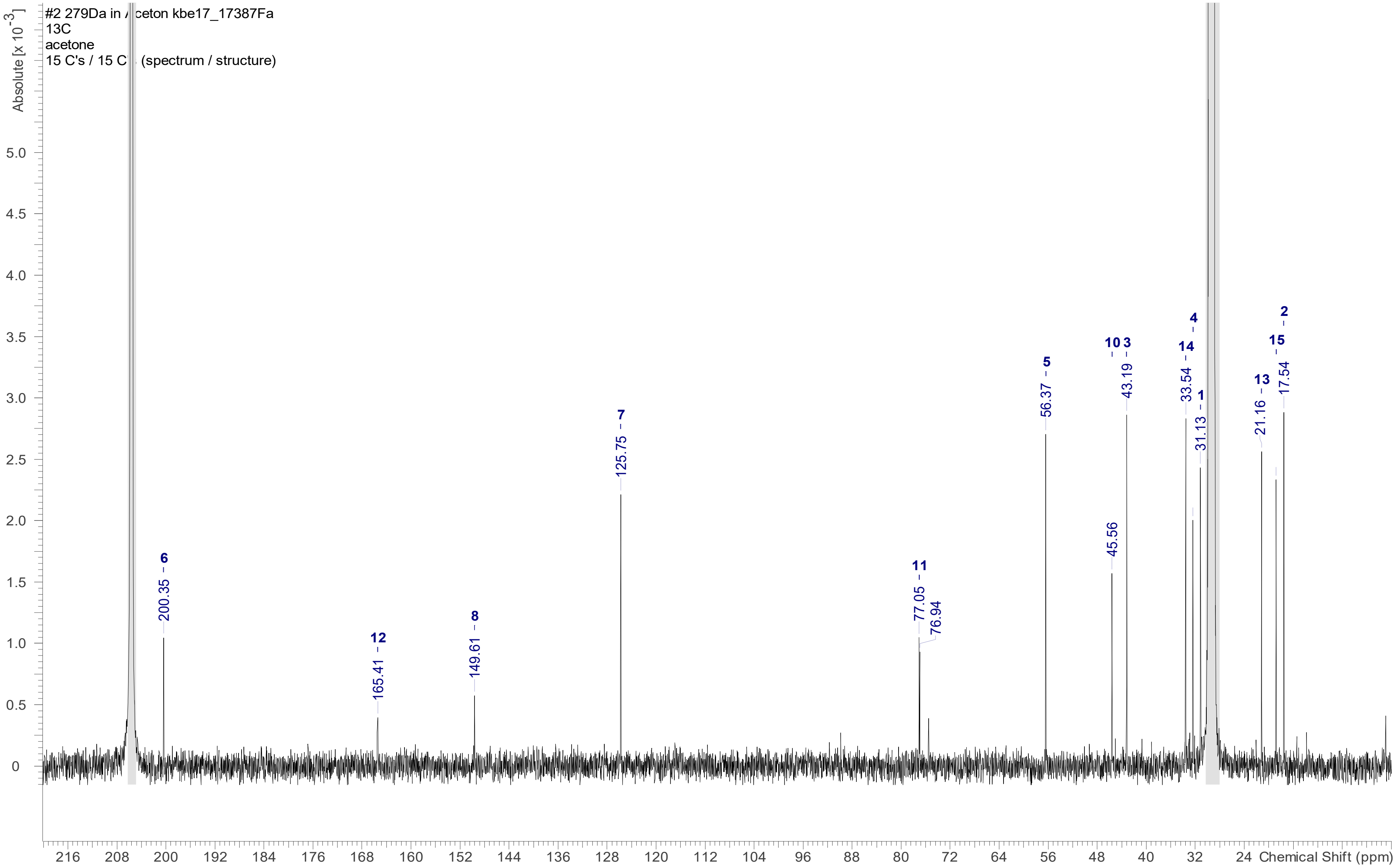

Figure S9 ^{13}C NMR spectrum (125 MHz, aceton-d_6) of **2**.

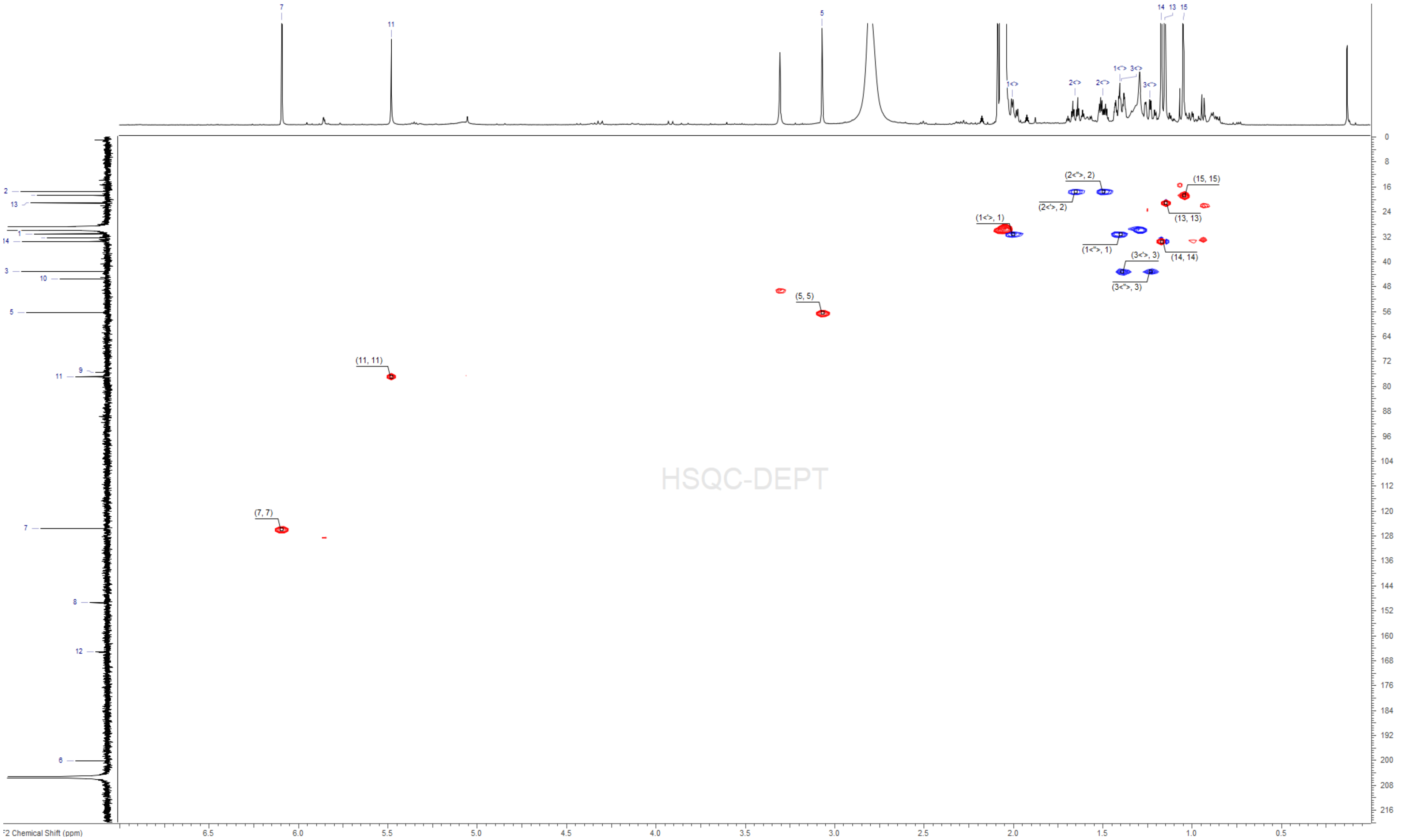

Figure S10 HSQC NMR spectrum (500 MHz, aceton-d_6) of **2**.

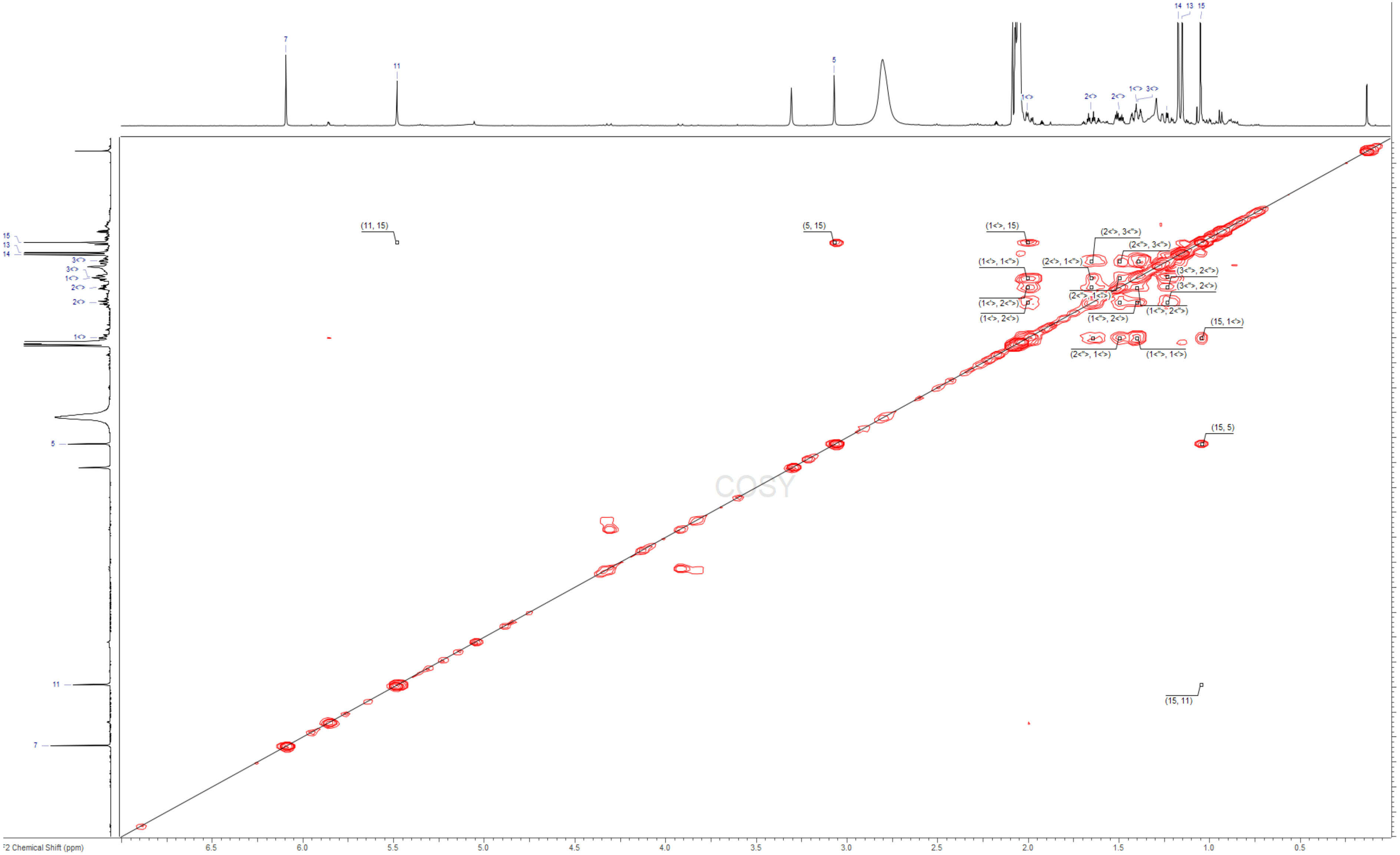

Figure S11 COSY NMR spectrum (500 MHz, aceton-d_6) of **2**.

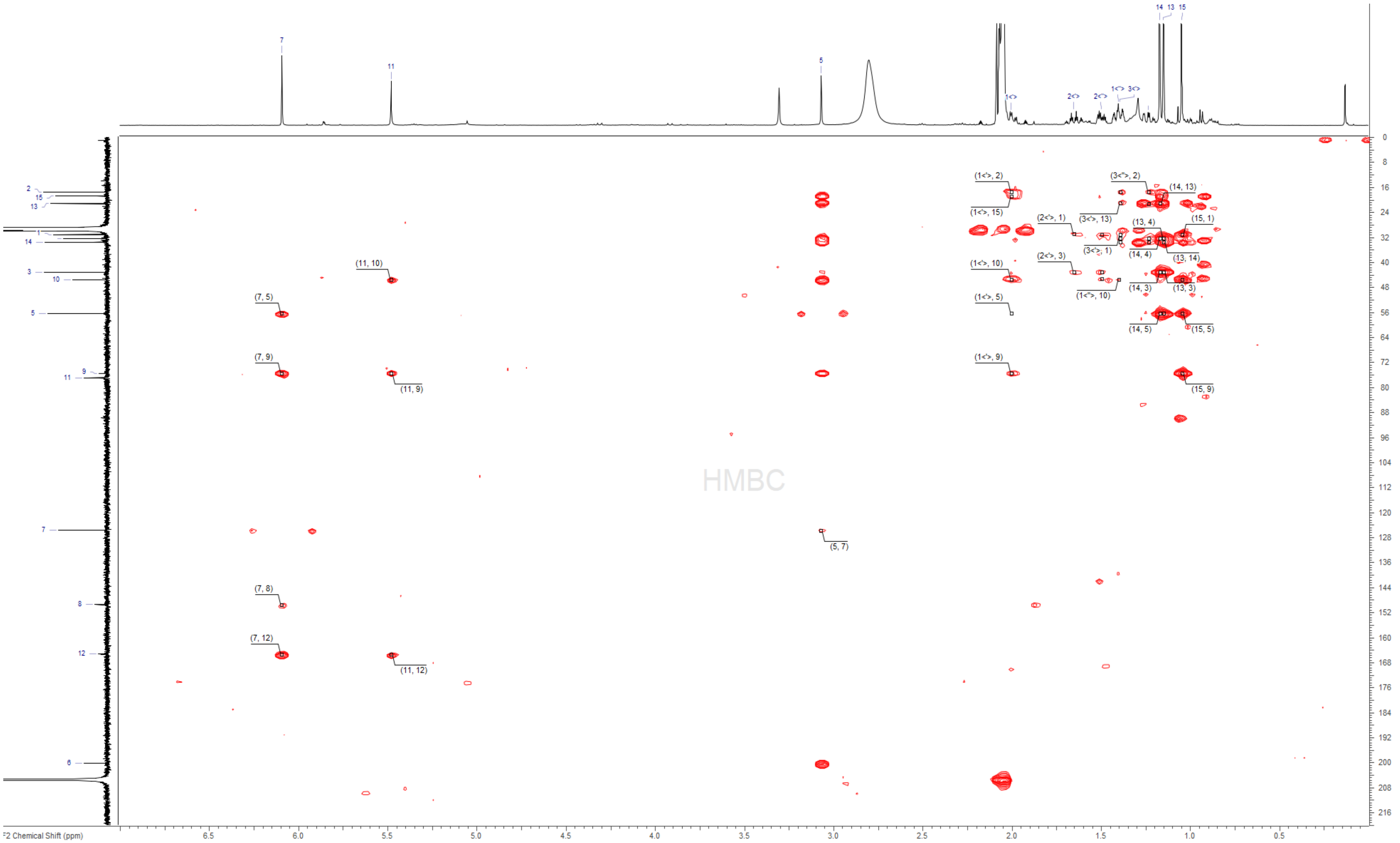

Figure S12 HMBC NMR spectrum (500 MHz, aceton-d_6) of **2**.

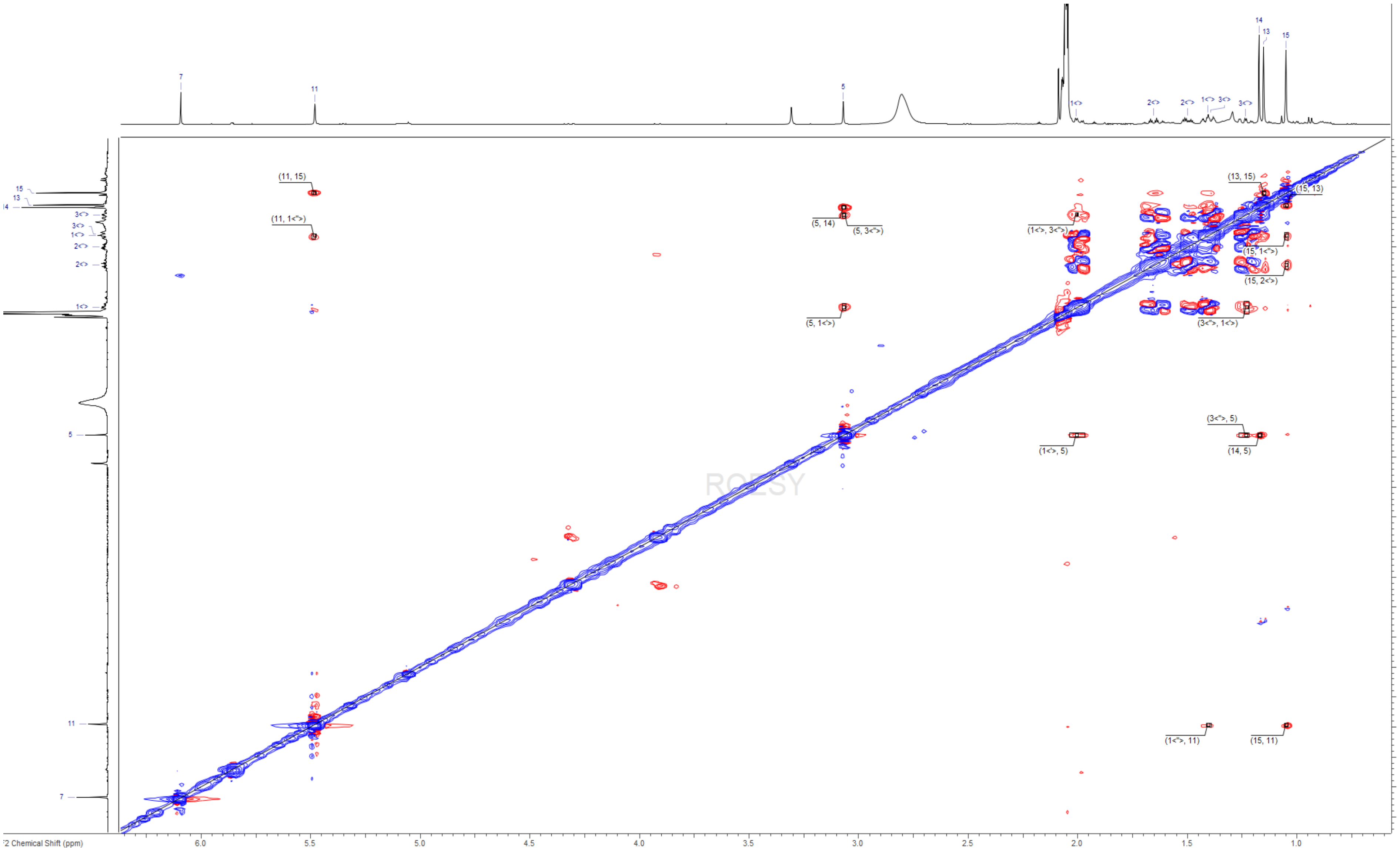

Figure S13 ROESY NMR spectrum (500 MHz, aceton-d_6) of **2**.

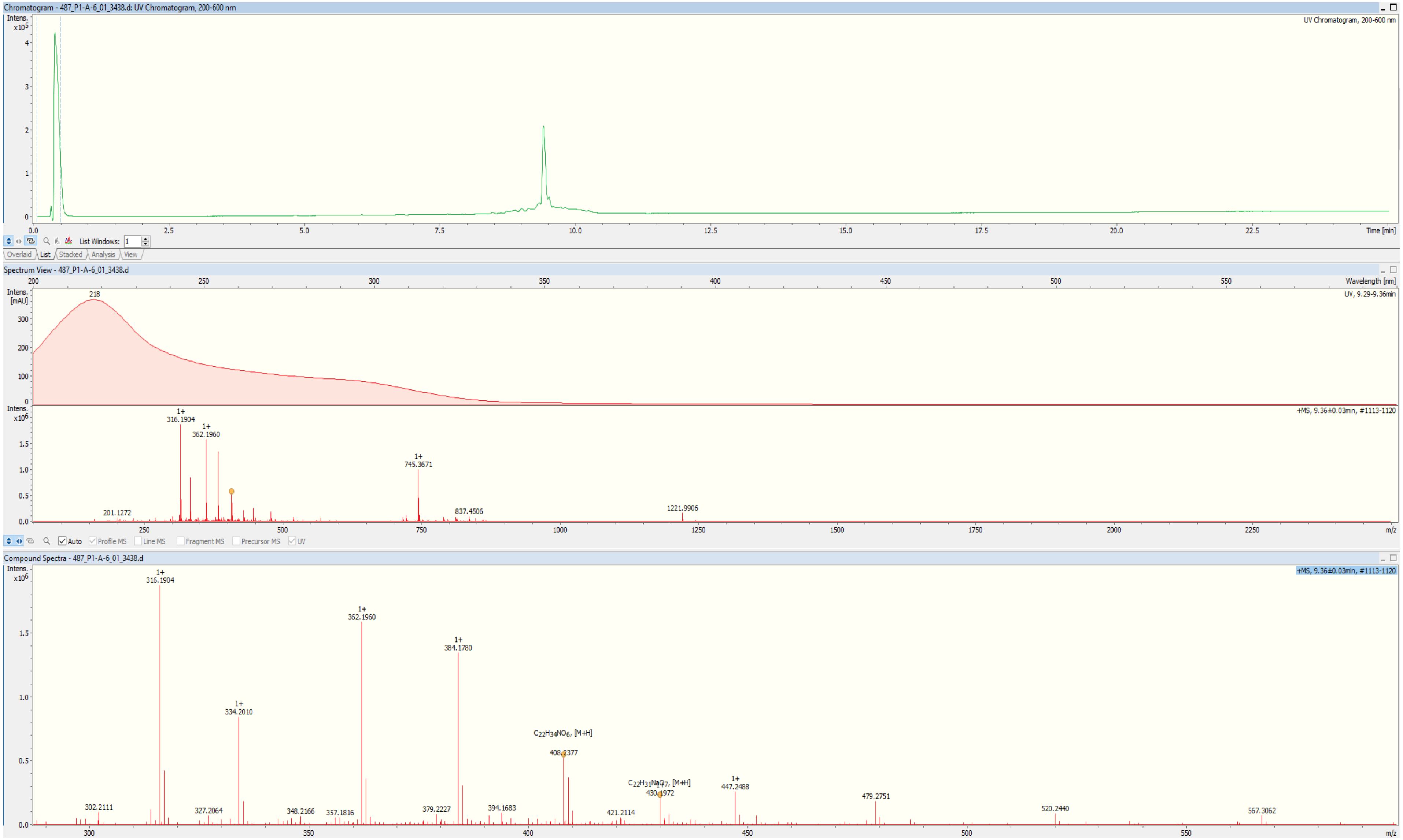

Figure S14 HPLC-HRESIMS data of **3**.

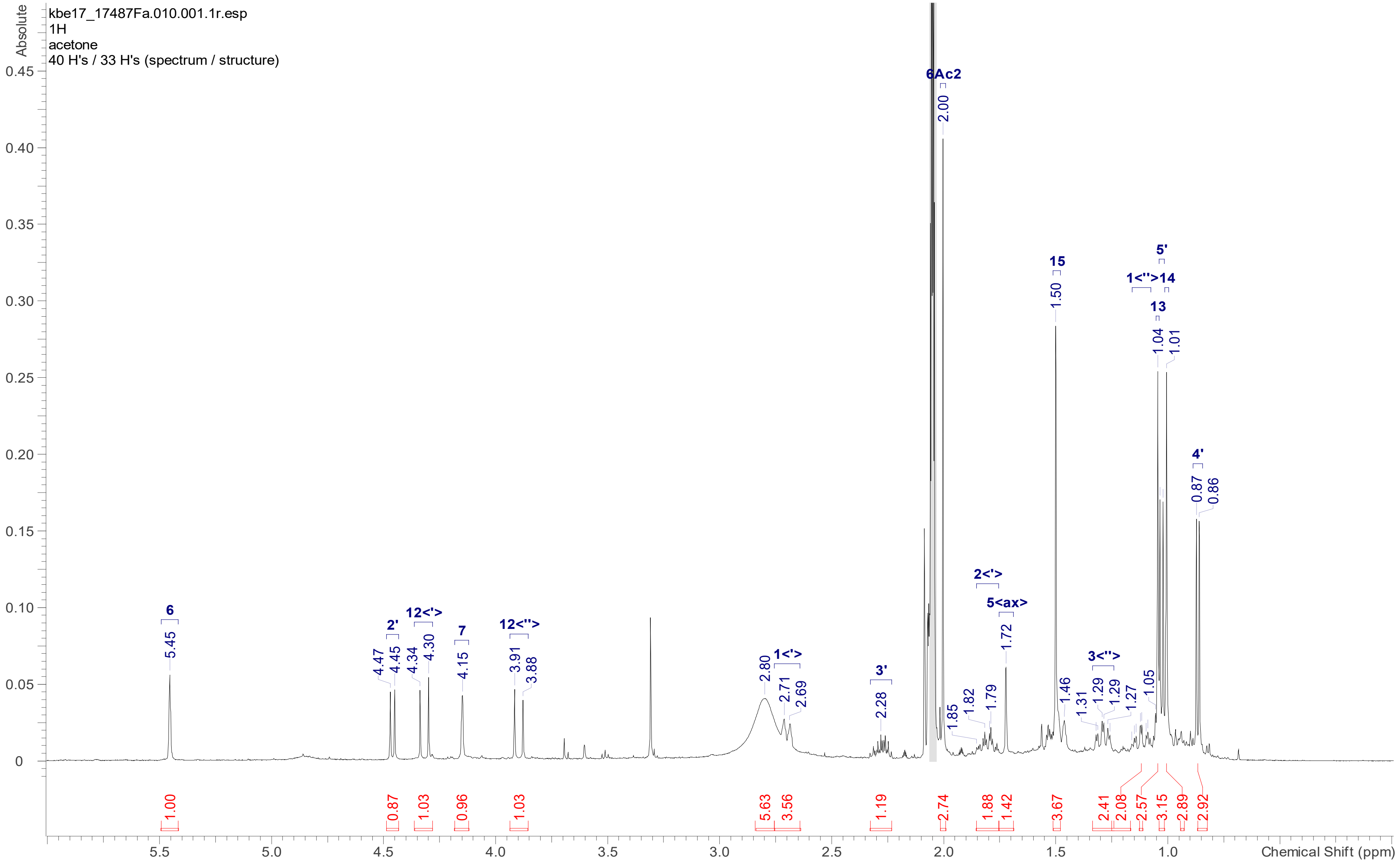

Figure S15 ^{1}H NMR spectrum (500 MHz, acetone-d_6) of **3**.

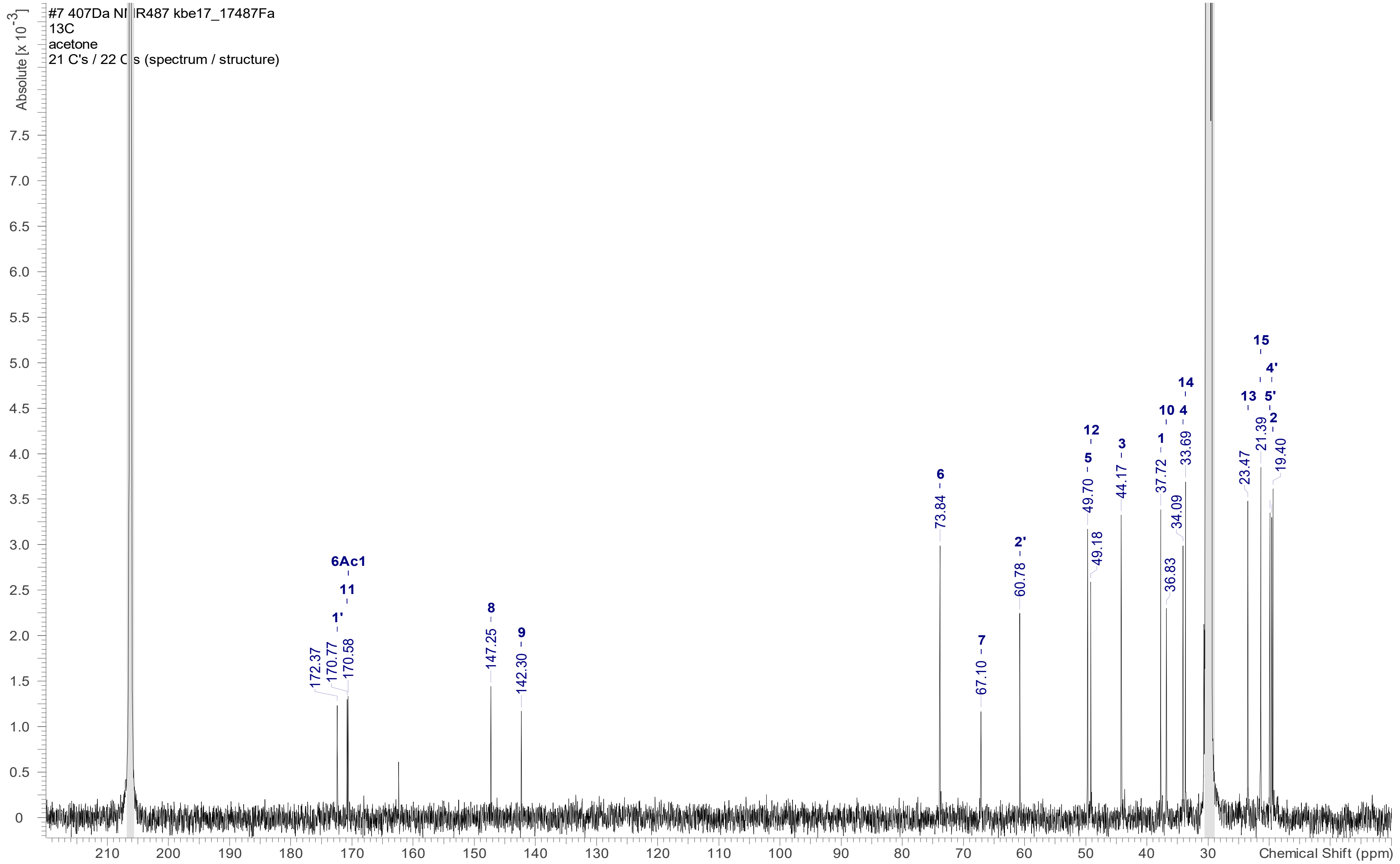

Figure S16 ^{13}C NMR spectrum (125 MHz, acetone-d_6) of **3**.

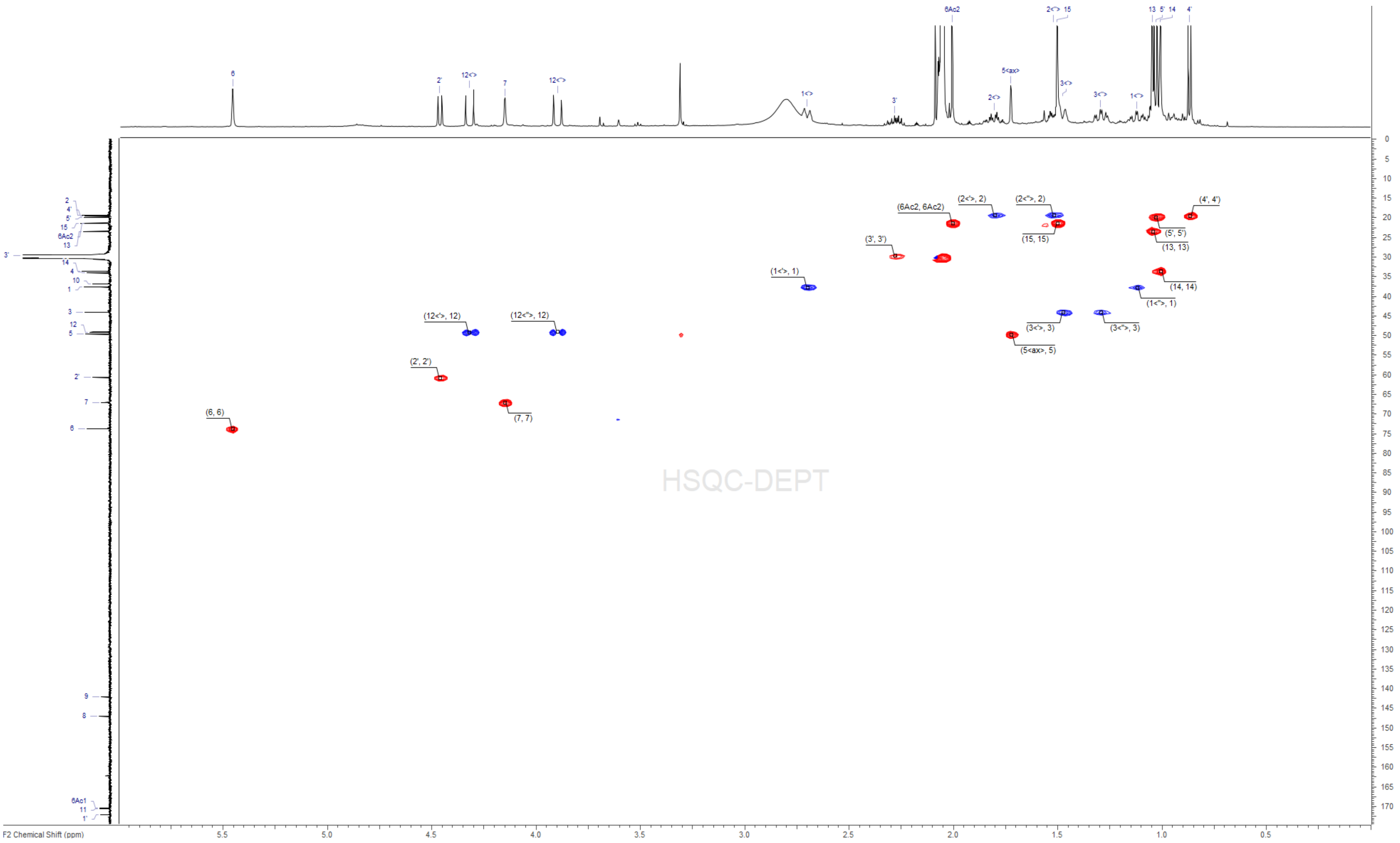

Figure S17 HSQC NMR spectrum (500 MHz, acetone-d_6) of **3**.

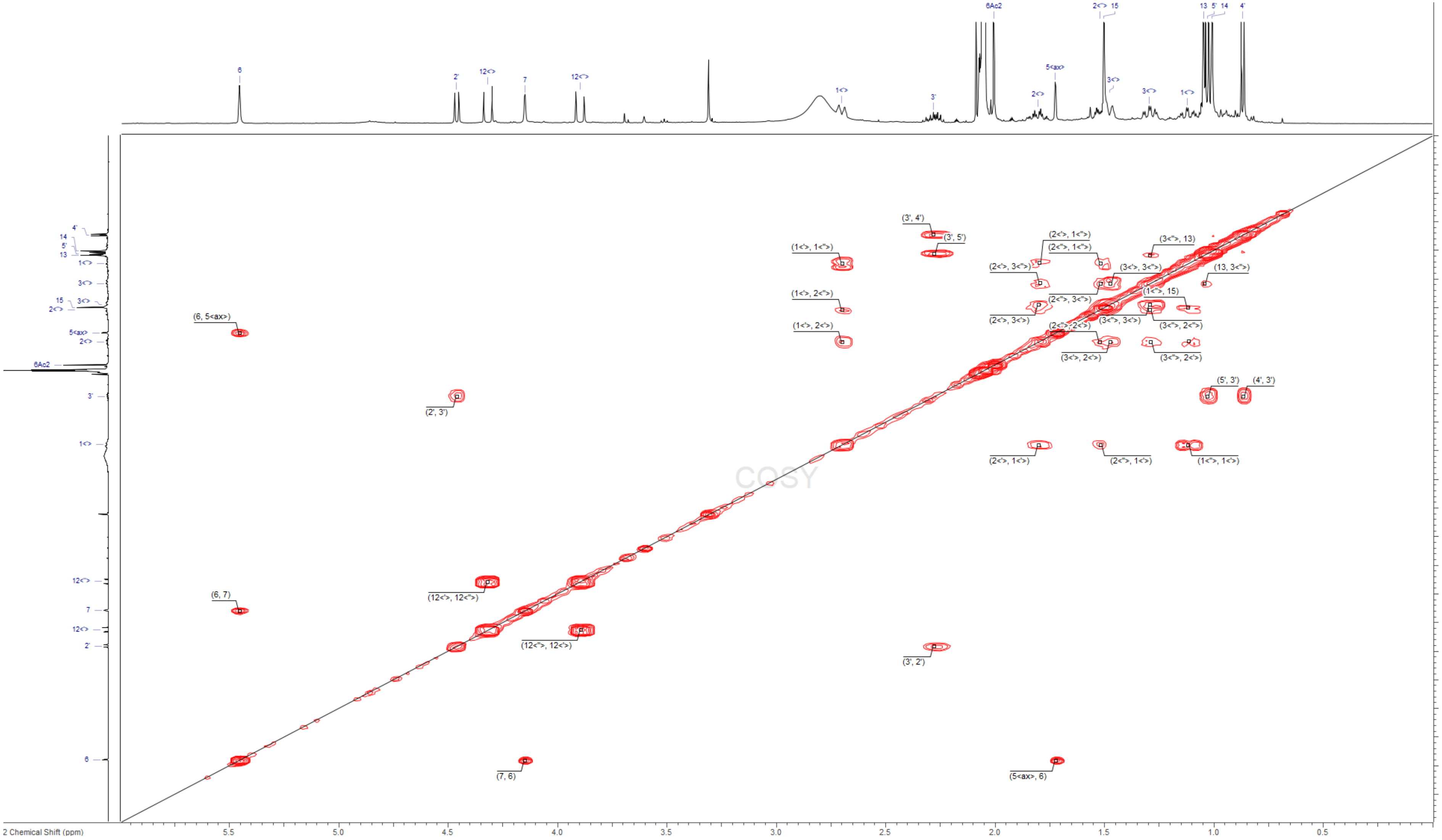

Figure S18 COSY NMR spectrum (500 MHz, acetone-d_6) of **3**.

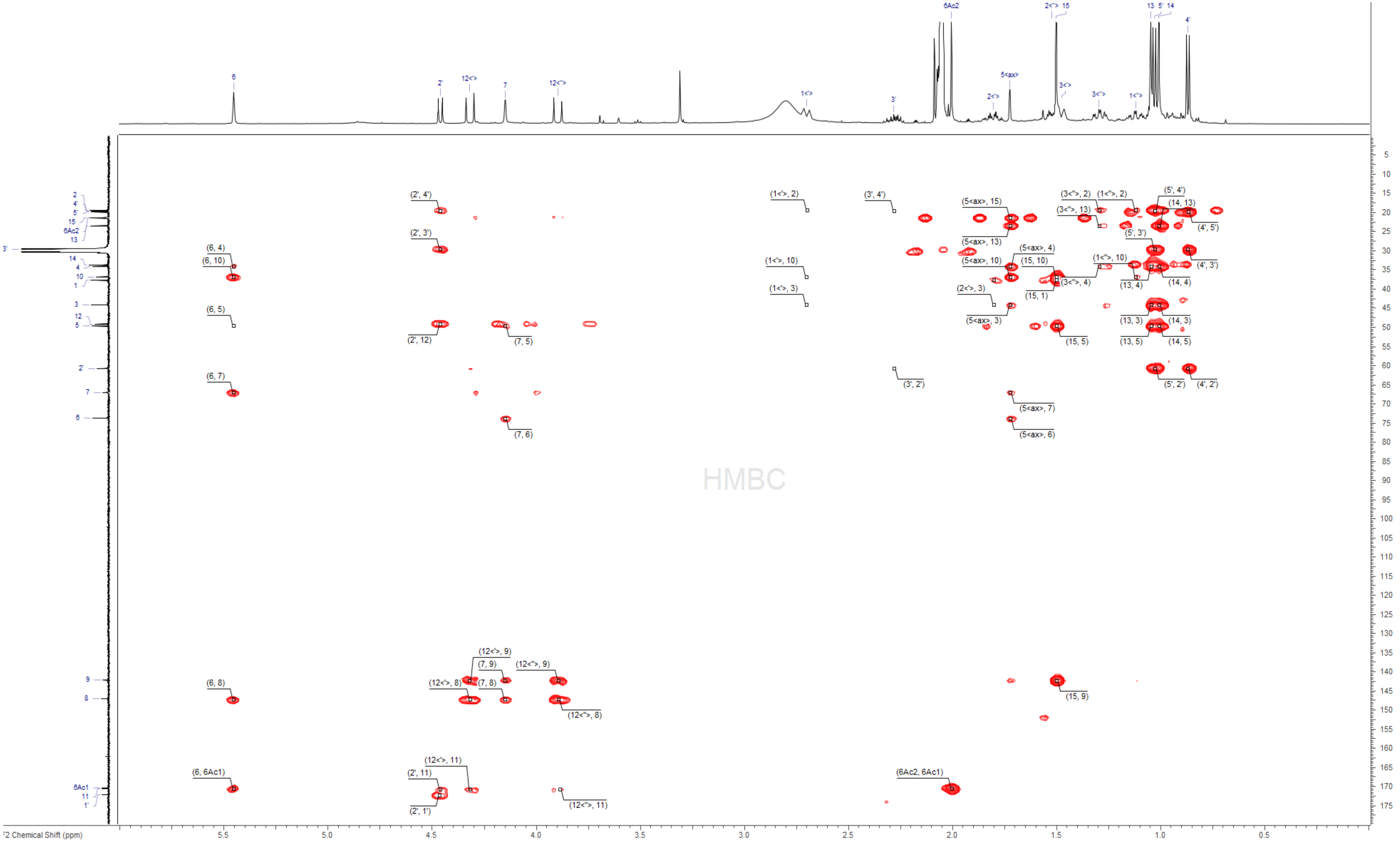

Figure S19 HMBC NMR spectrum (500 MHz, acetone-d_6) of **3**.

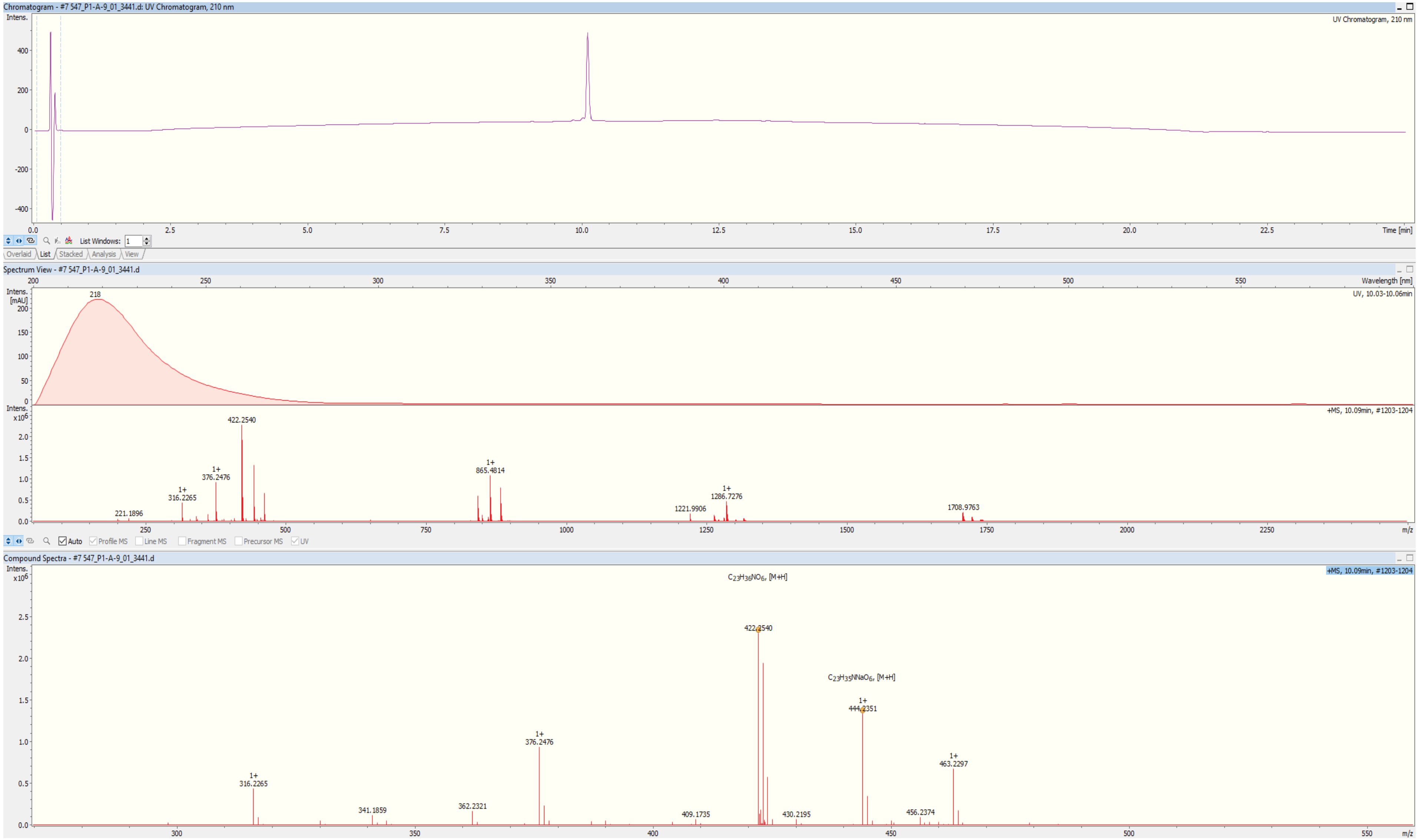

Figure S20 HPLC-HRESIMS data of **4**.

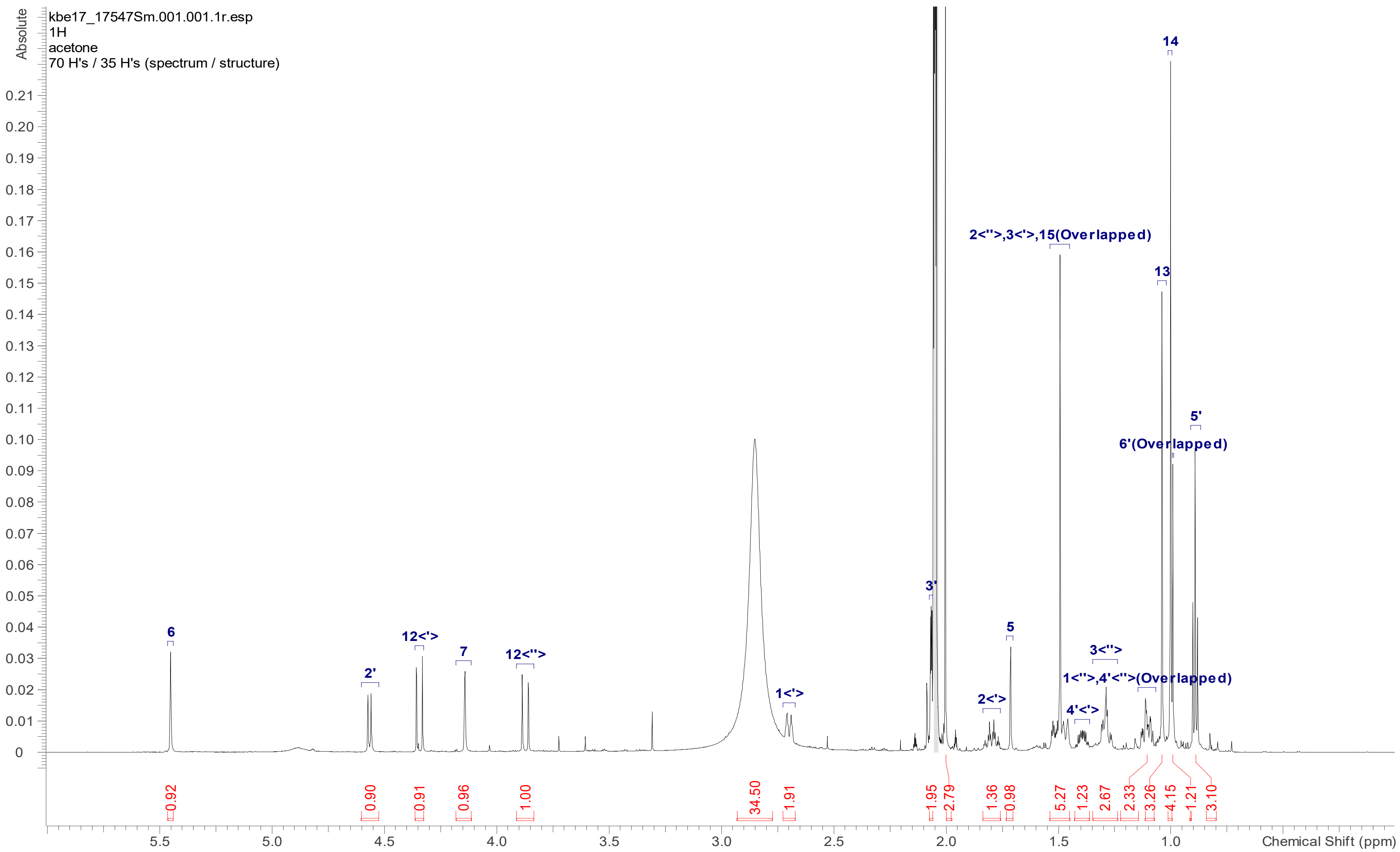

Figure S21 ^{1}H NMR spectrum (500 MHz, acetone-d_6) of **4**.

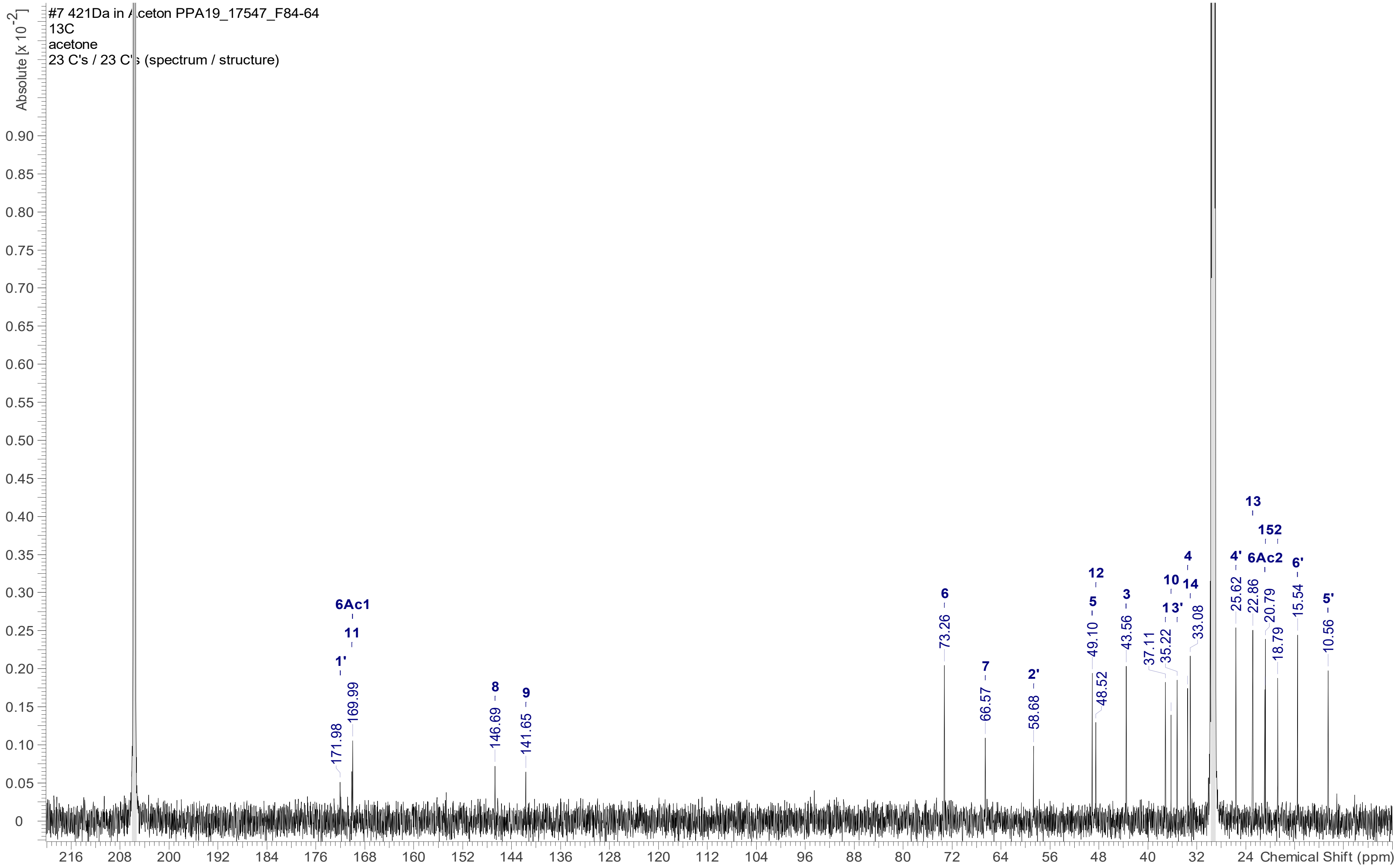

Figure S22 ^{13}C NMR spectrum (125 MHz, acetone-d_6) of **4**.

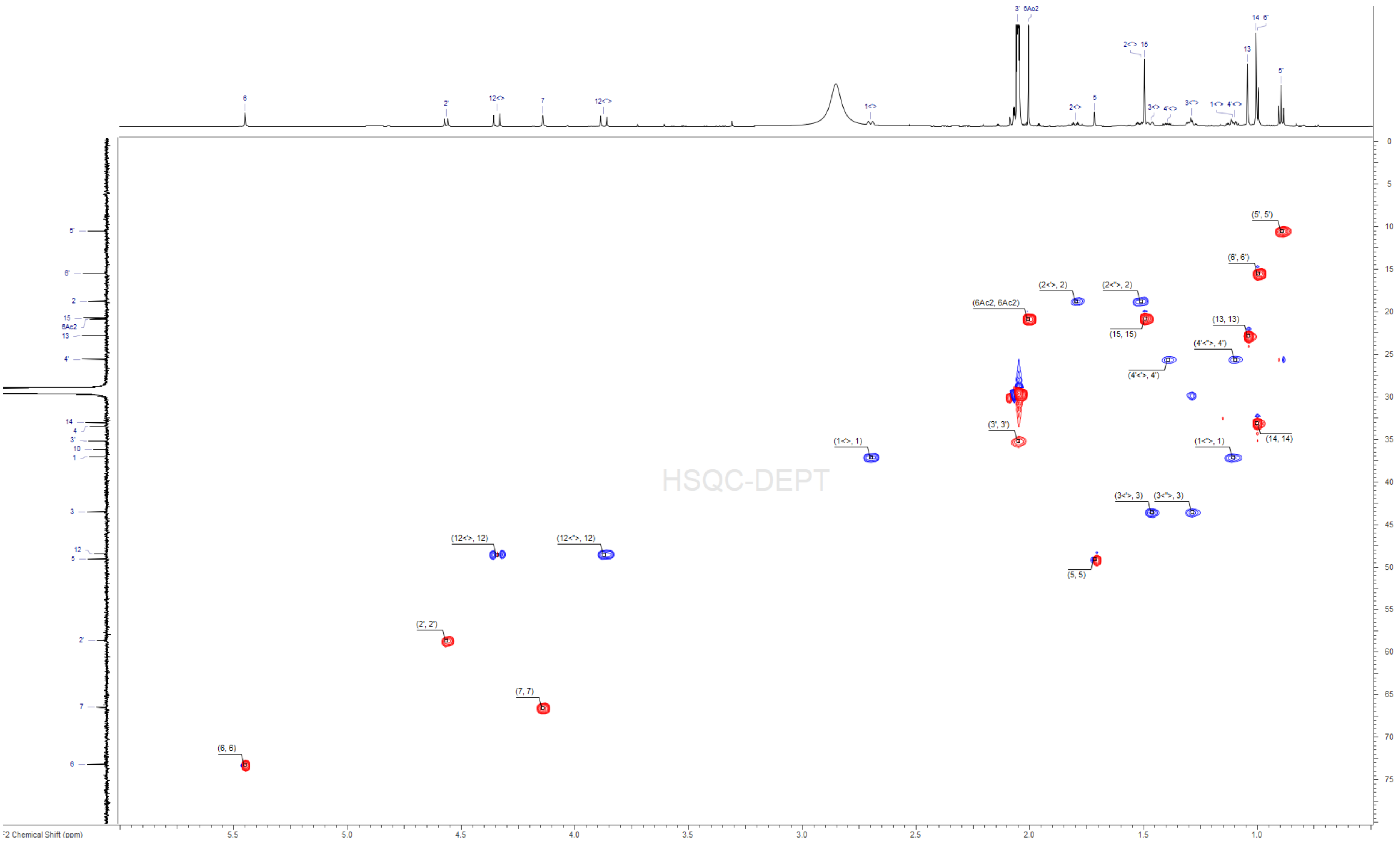

Figure S23 HSQC NMR spectrum (500 MHz, acetone-d_6) of **4**.

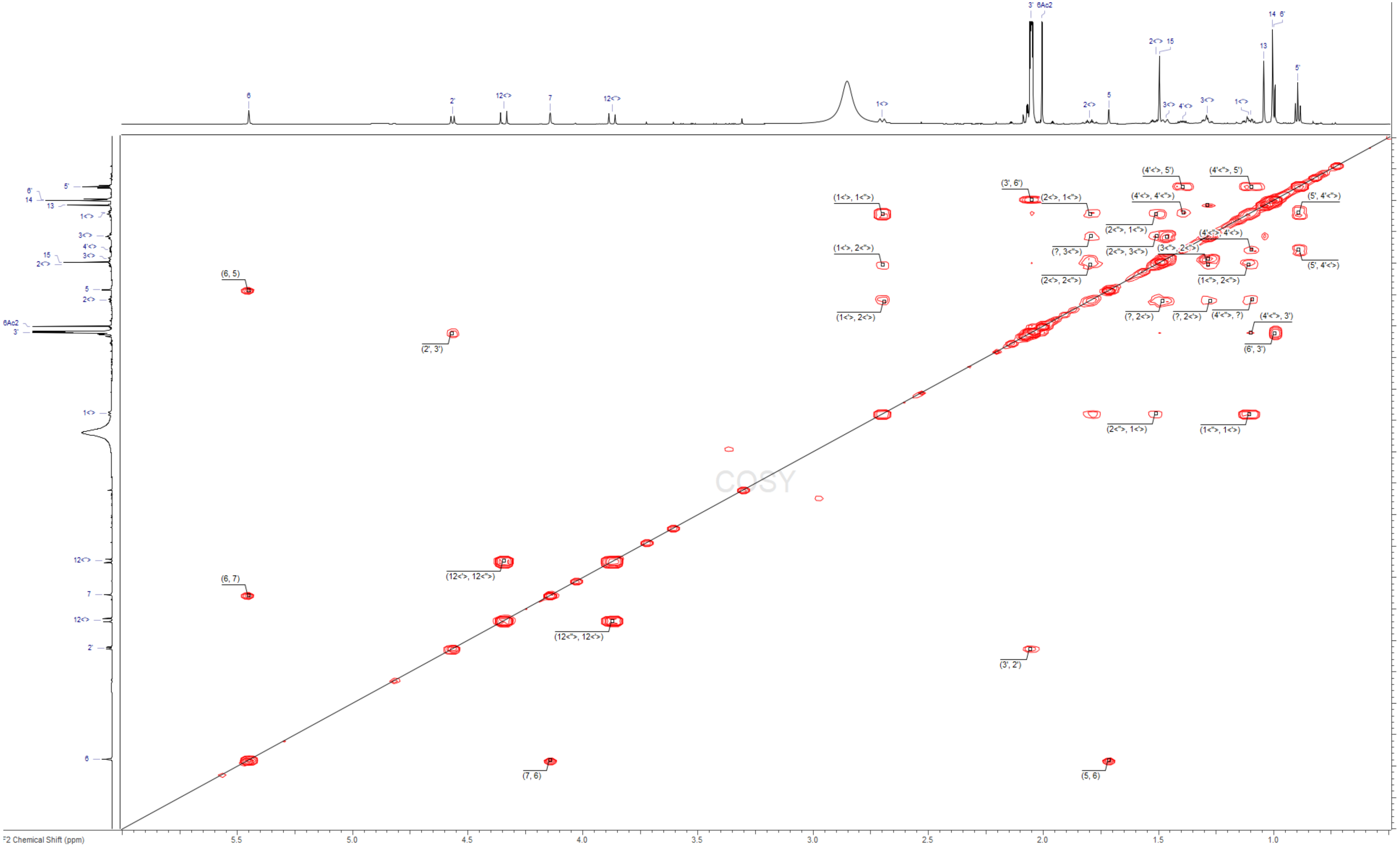

Figure S24 COSY NMR spectrum (500 MHz, acetone-d_6) of **4**.

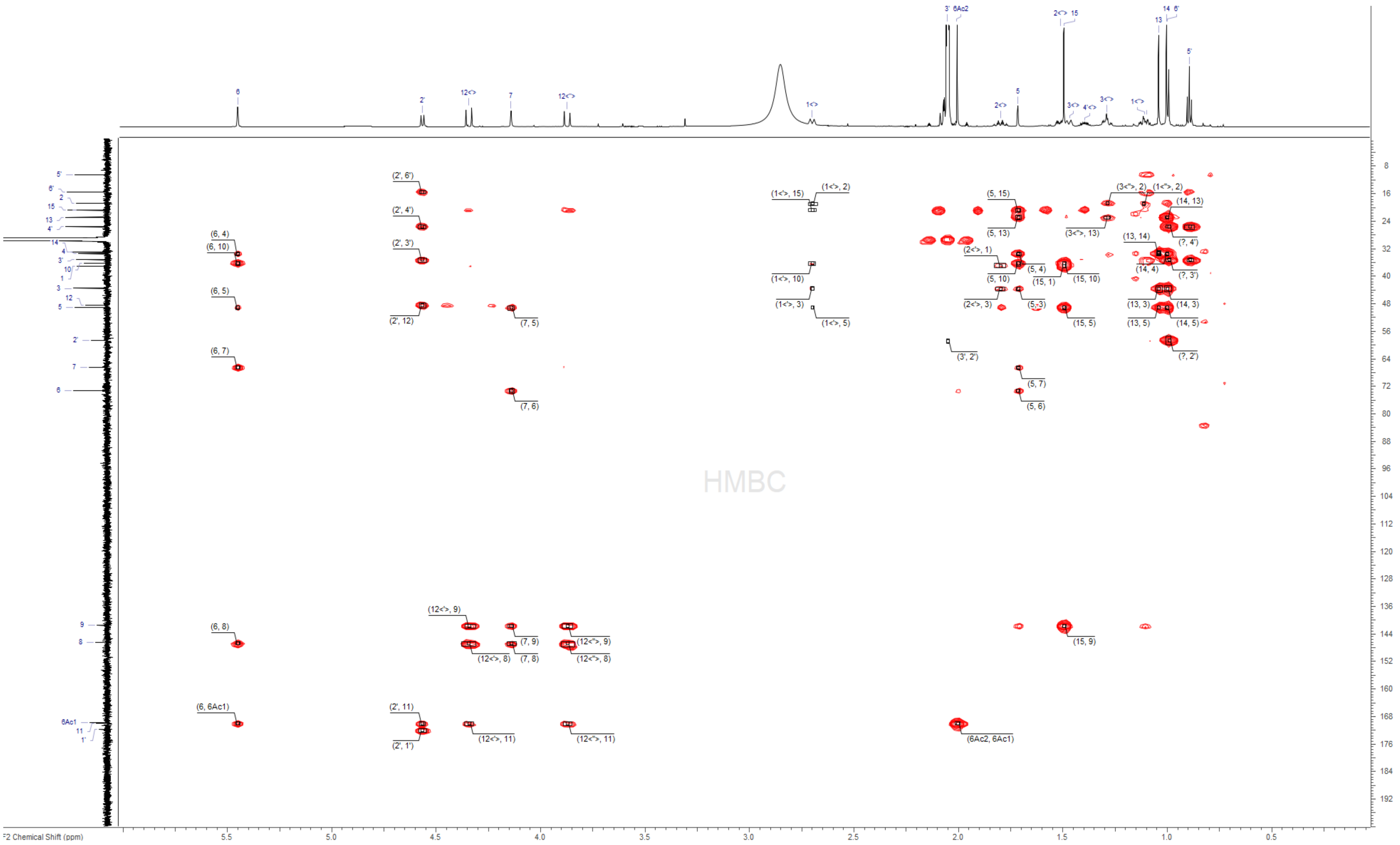

Figure S25 HMBC NMR spectrum (500 MHz, acetone-d_6) of **4**.

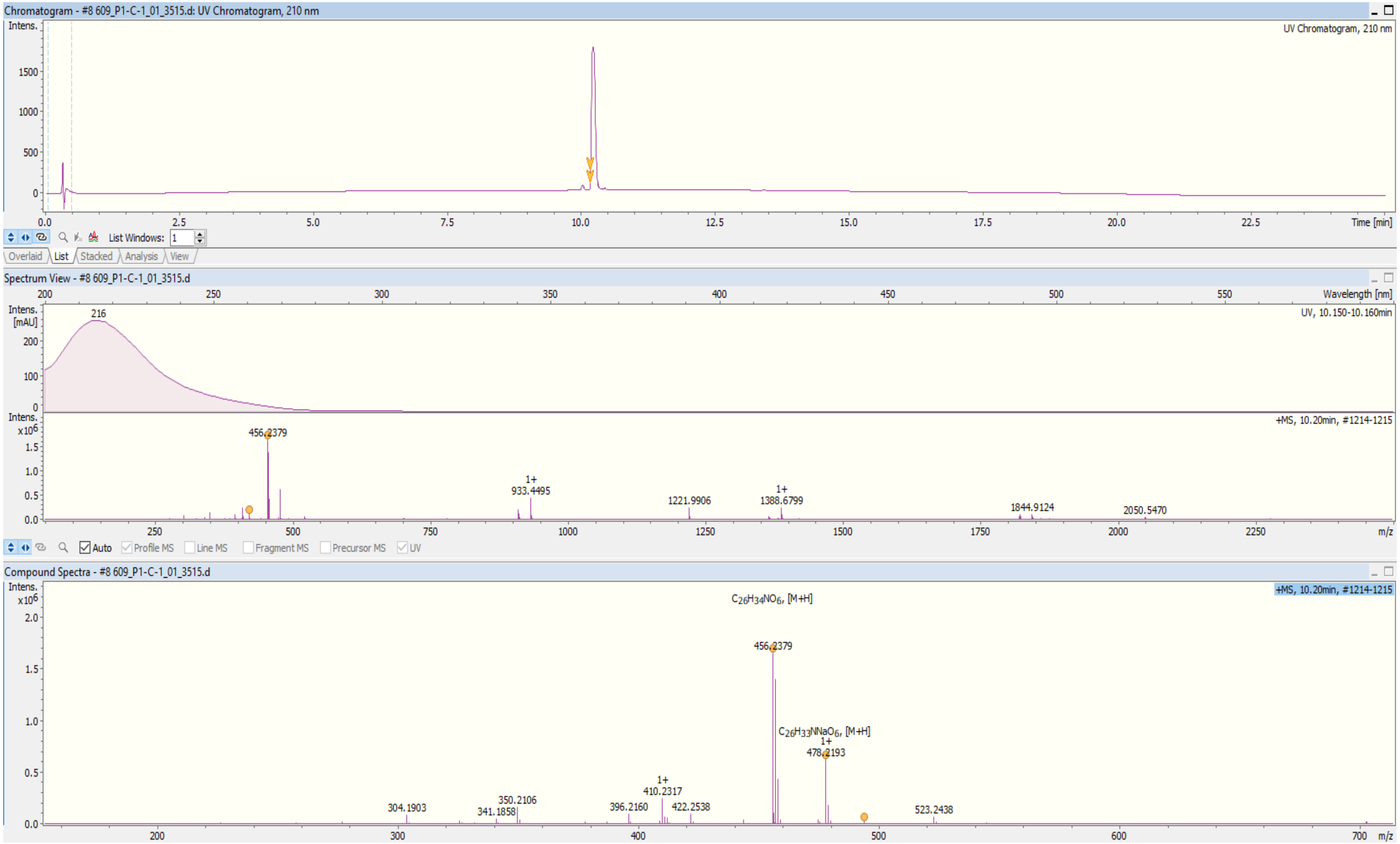

Figure S26 HPLC-HRESIMS data of **5**.

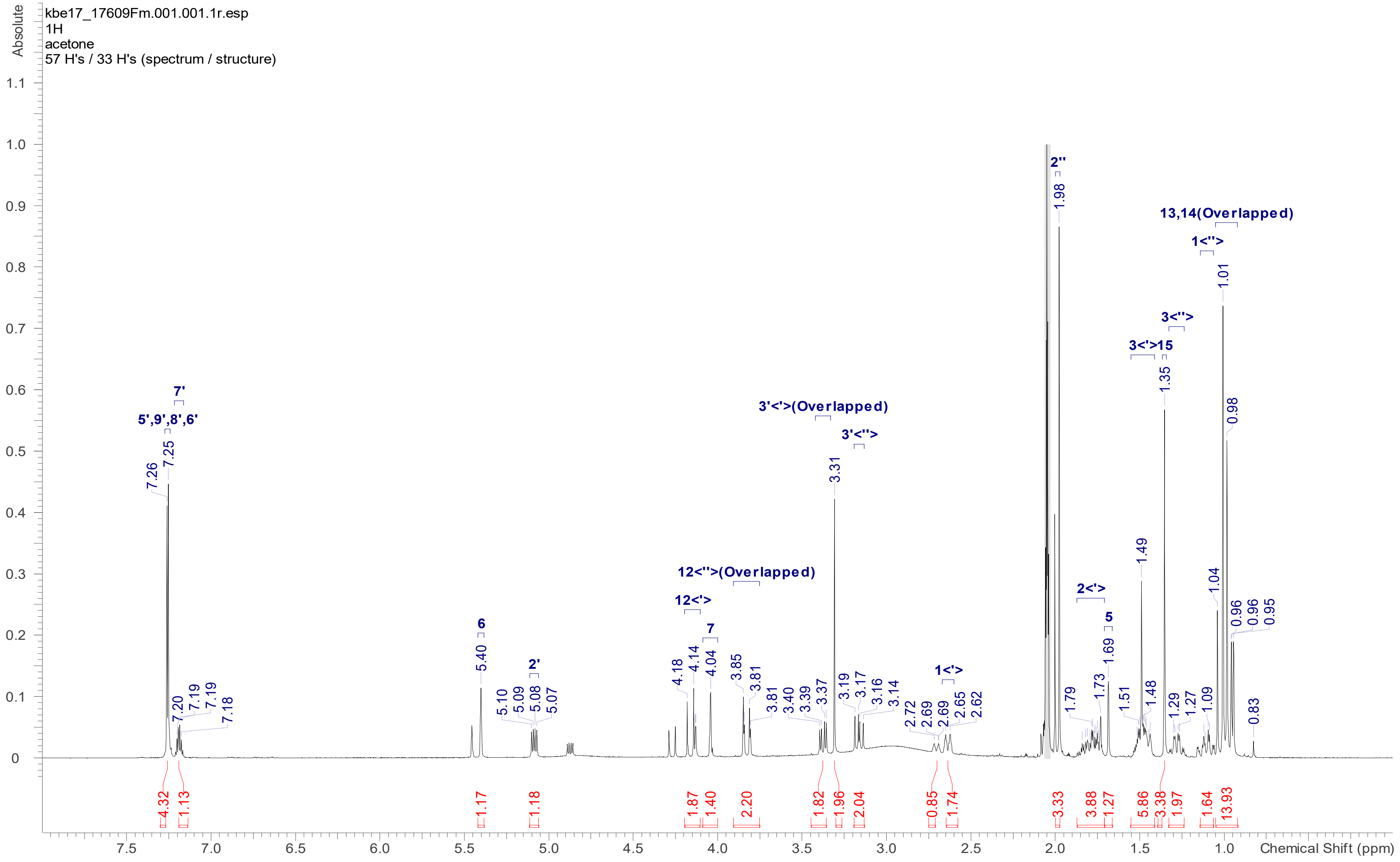

Figure S27 ^{1}H NMR spectrum (500 MHz, acetone-d_6) of **5**.

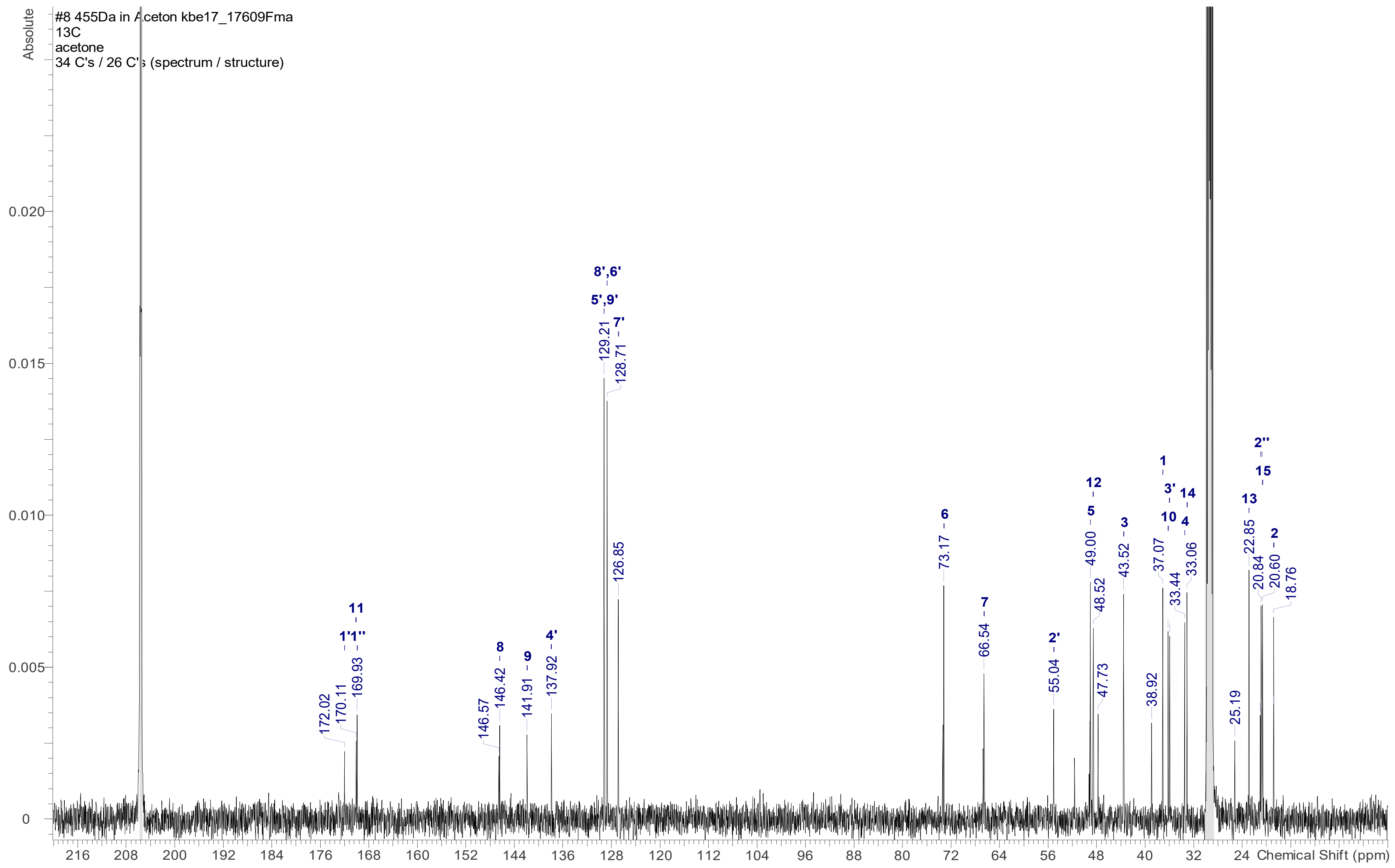

Figure S28 ^{13}C NMR spectrum (125 MHz, acetone-d_6) of **5**.

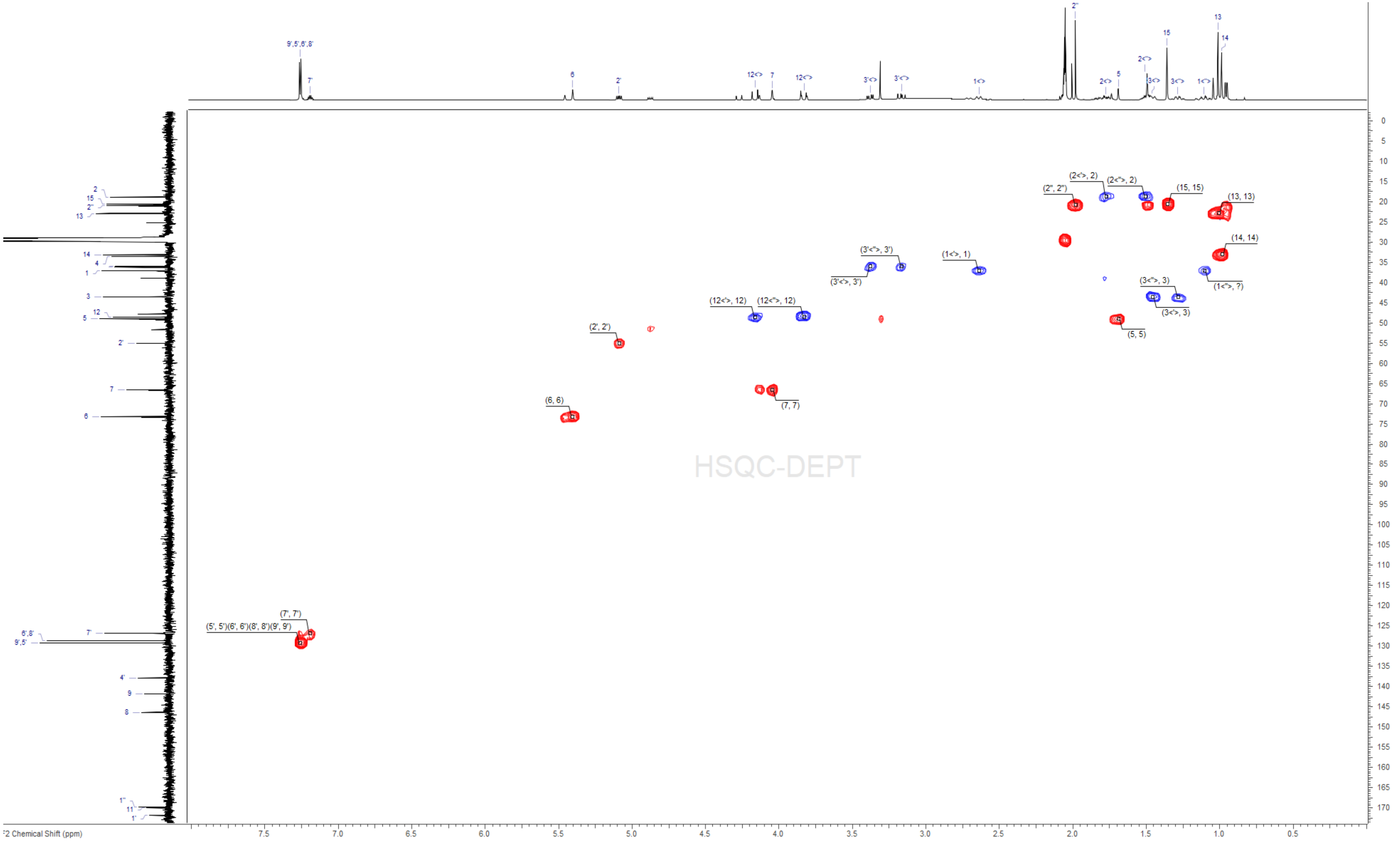

Figure S29 HSQC NMR spectrum (500 MHz, acetone-d_6) of **5**.

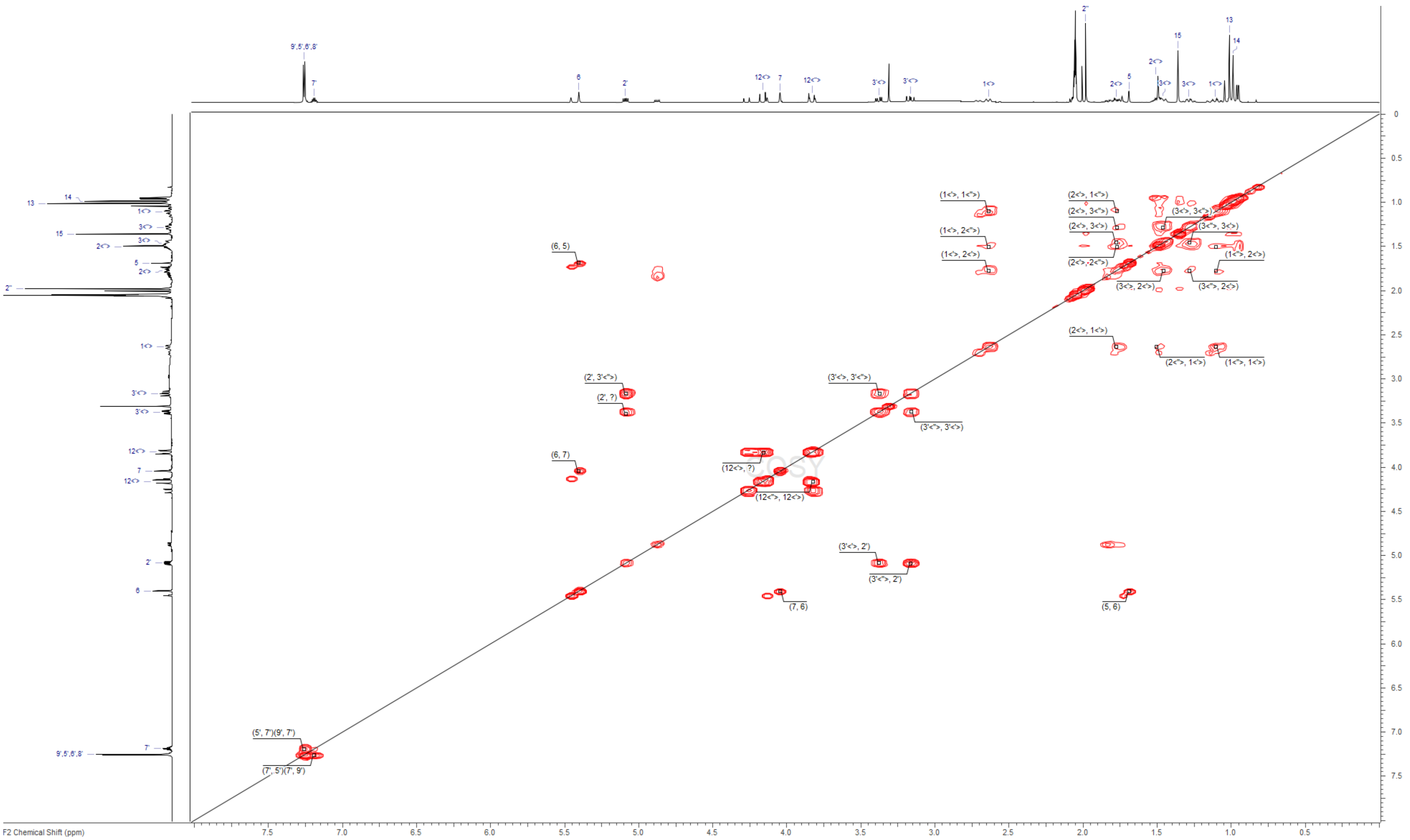

Figure S30 COSY NMR spectrum (500 MHz, acetone-d_6) of **5**.

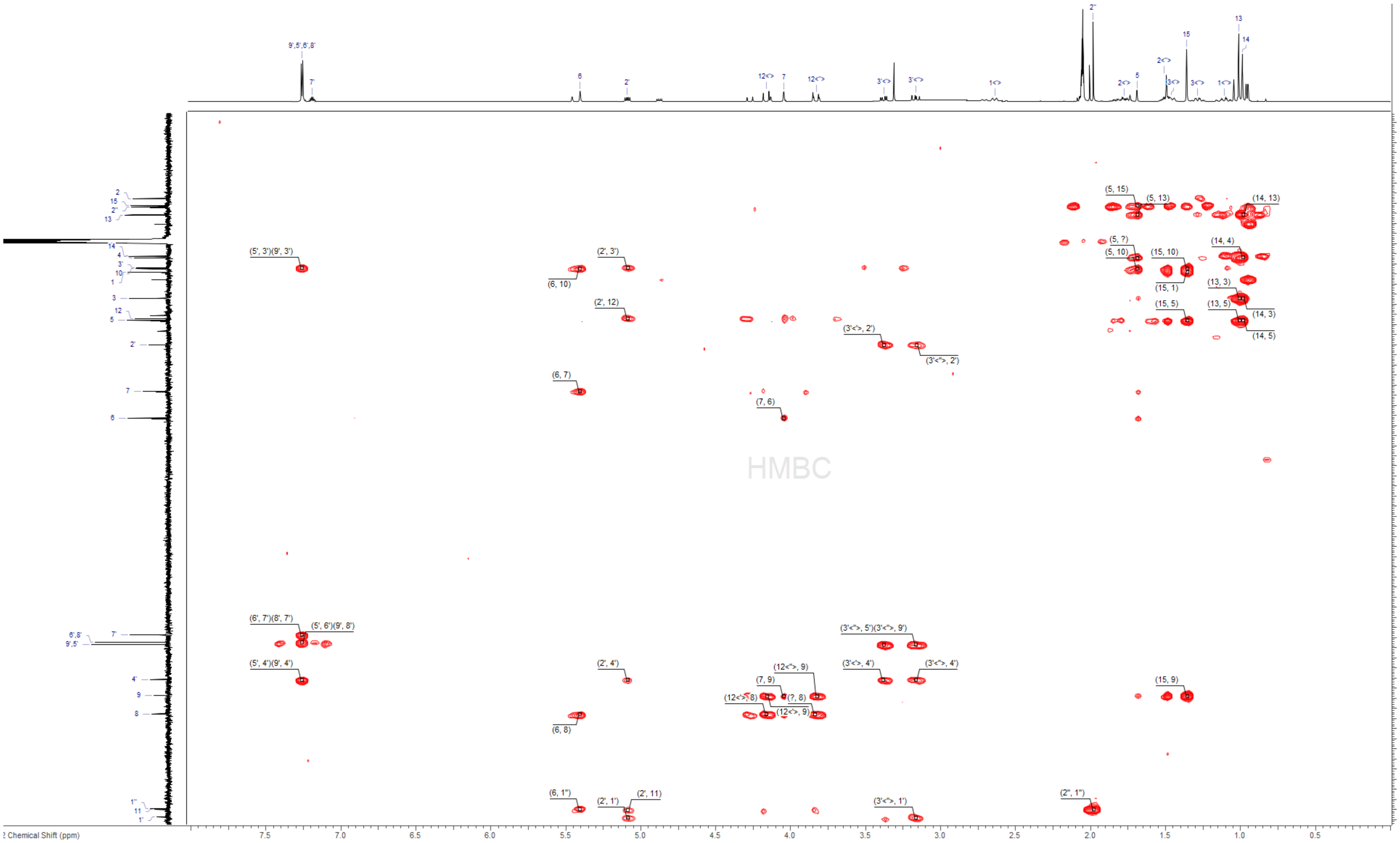

Figure S31 HMBC NMR spectrum (500 MHz, acetone-d_6) of **5**.

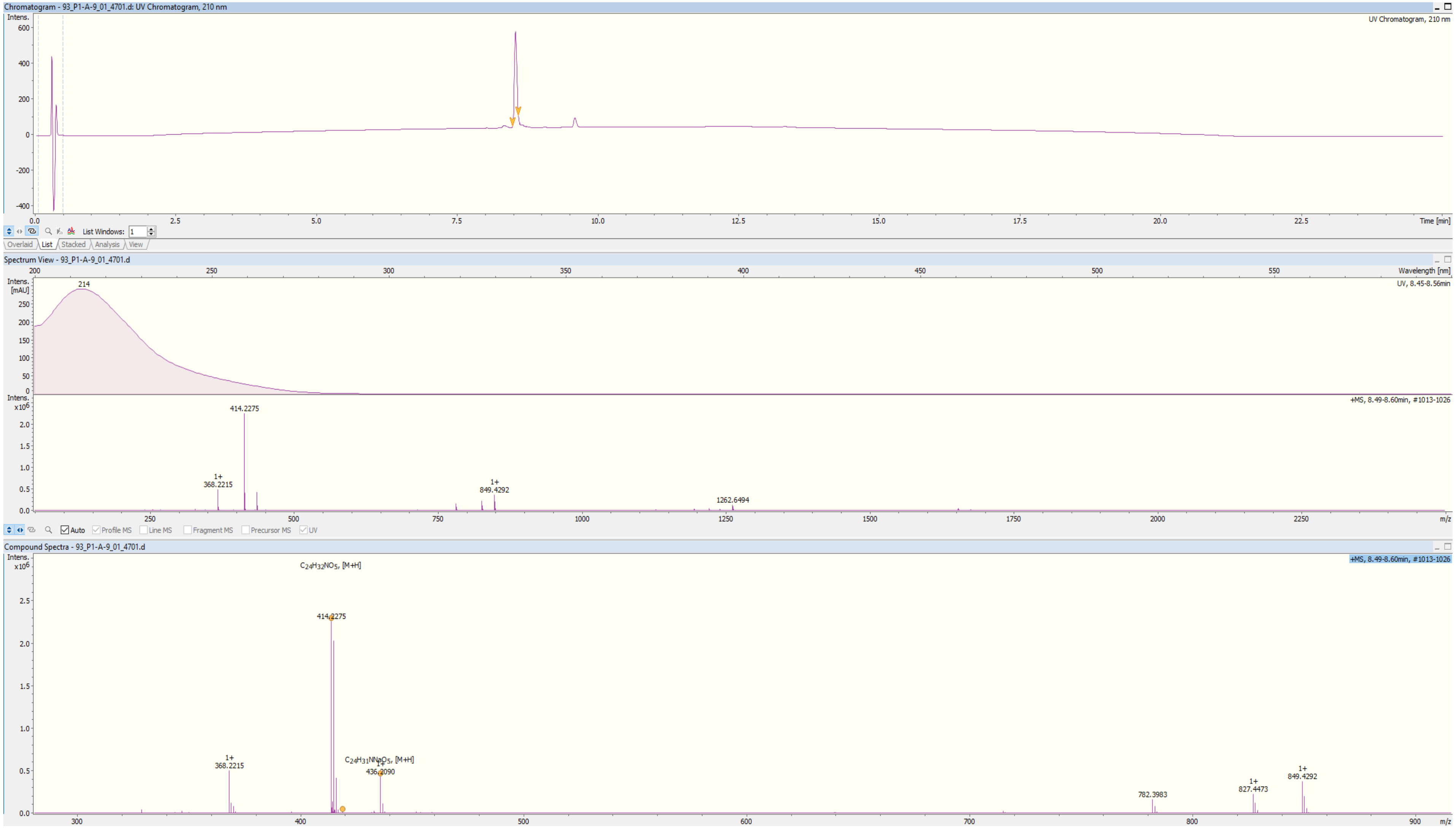

Figure S32 HPLC-HRESIMS data of **6**.

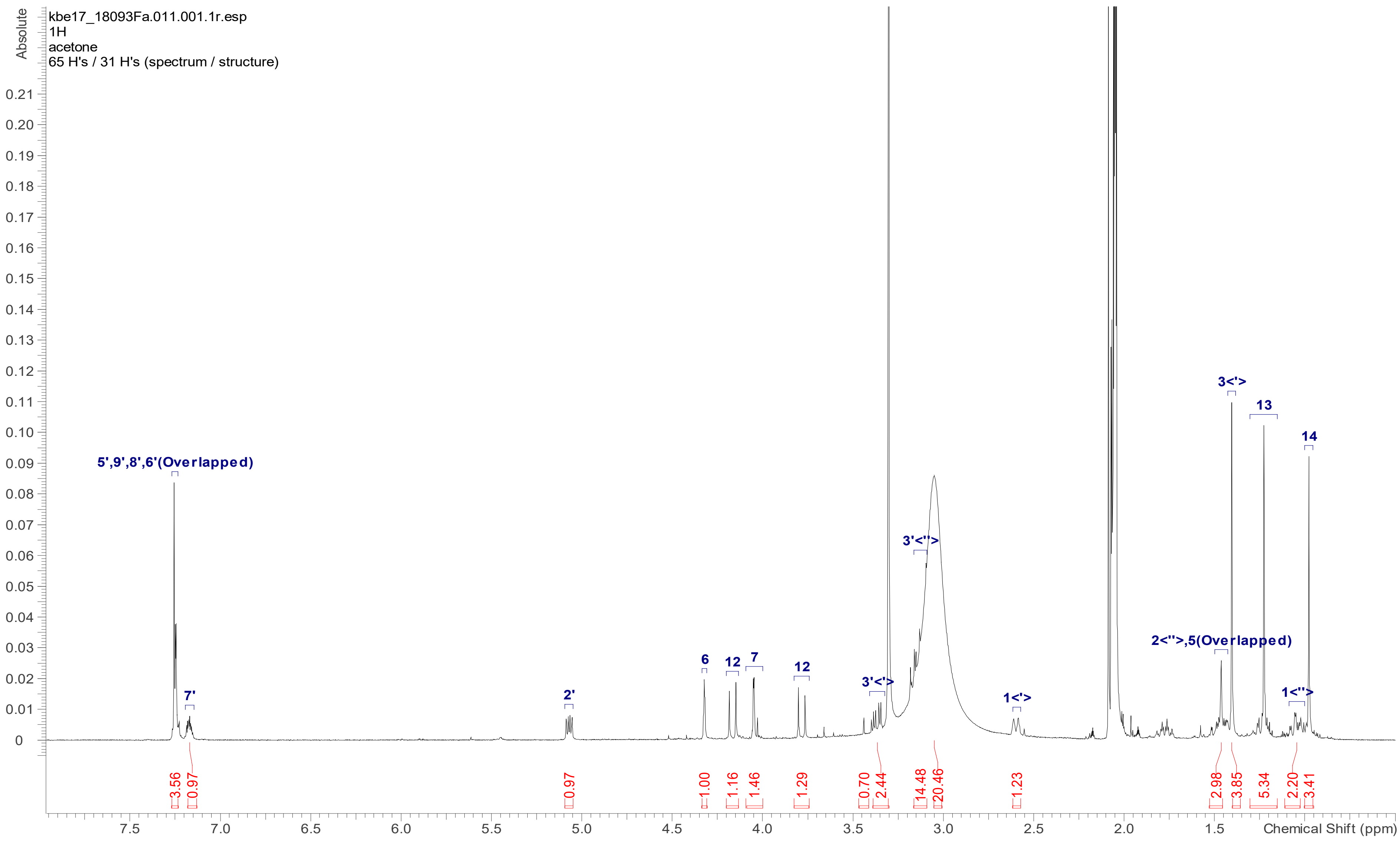

Figure S33 ^{1}H NMR spectrum (500 MHz, acetone-d_6) of **6**.

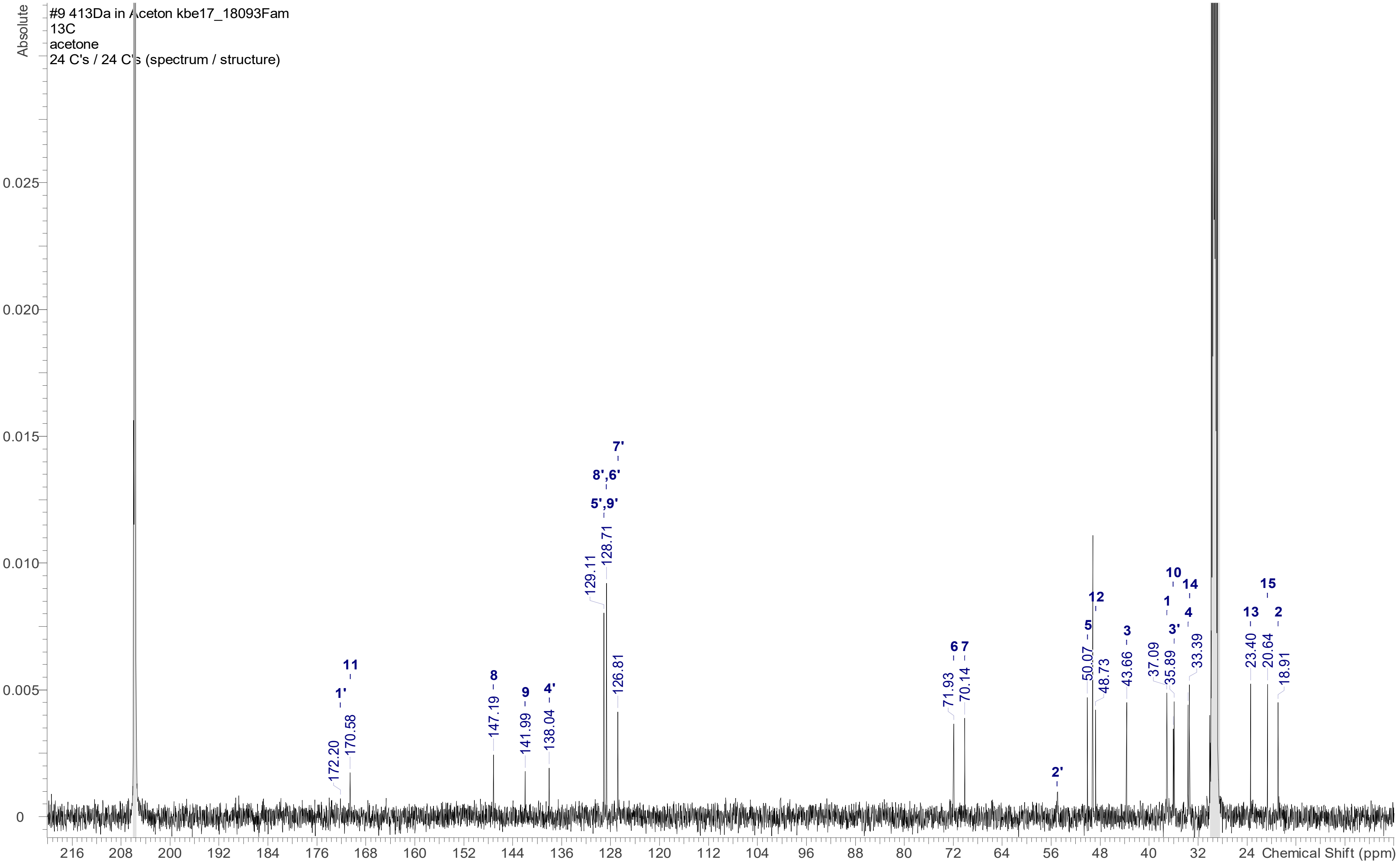

Figure S34 ^{13}C NMR spectrum (125 MHz, acetone-d_6) of **6**.

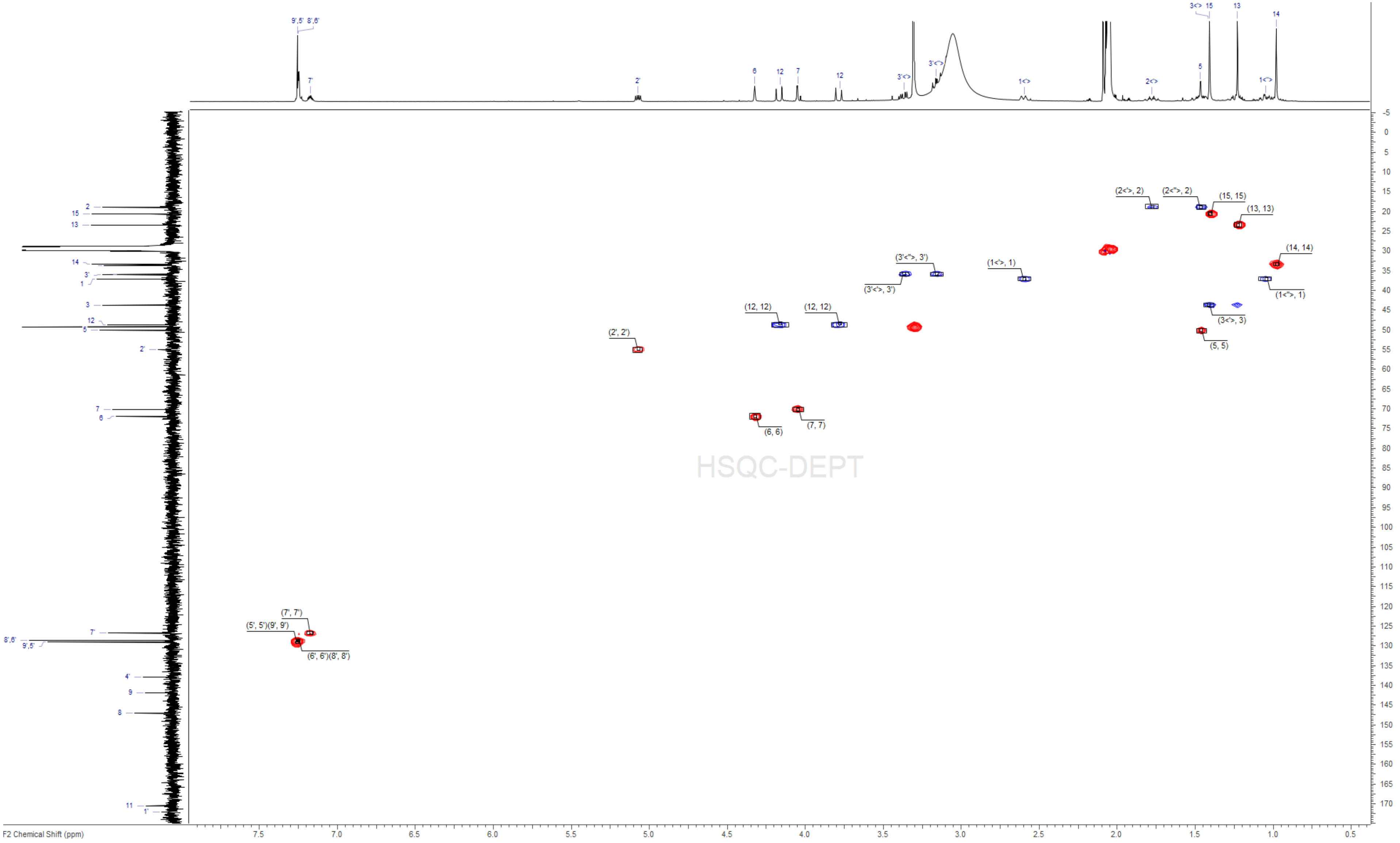

Figure S35 HSQC NMR spectrum (500 MHz, acetone-d_6) of **6**.

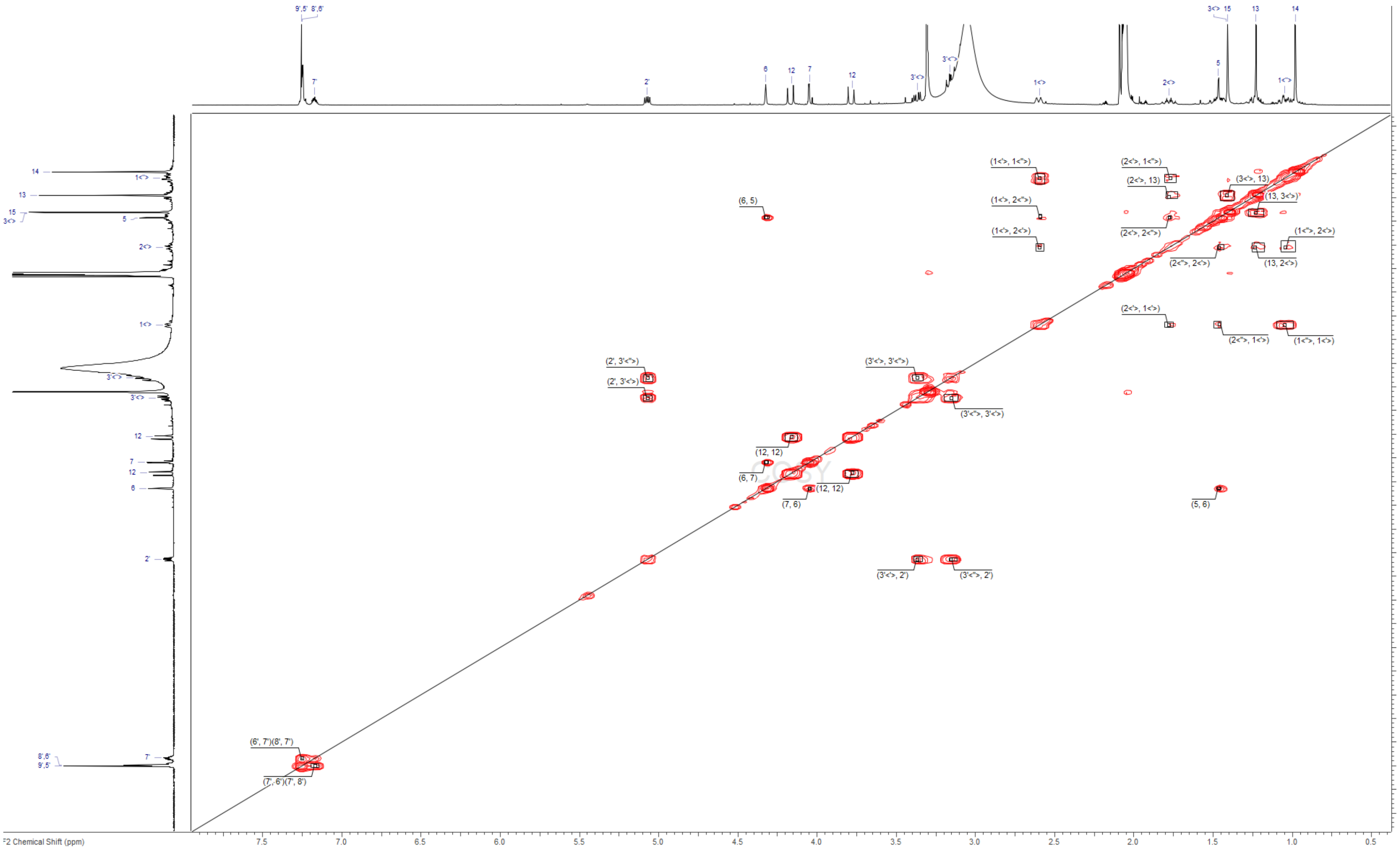

Figure S36 COSY NMR spectrum (500 MHz, acetone-d_6) of **6**.

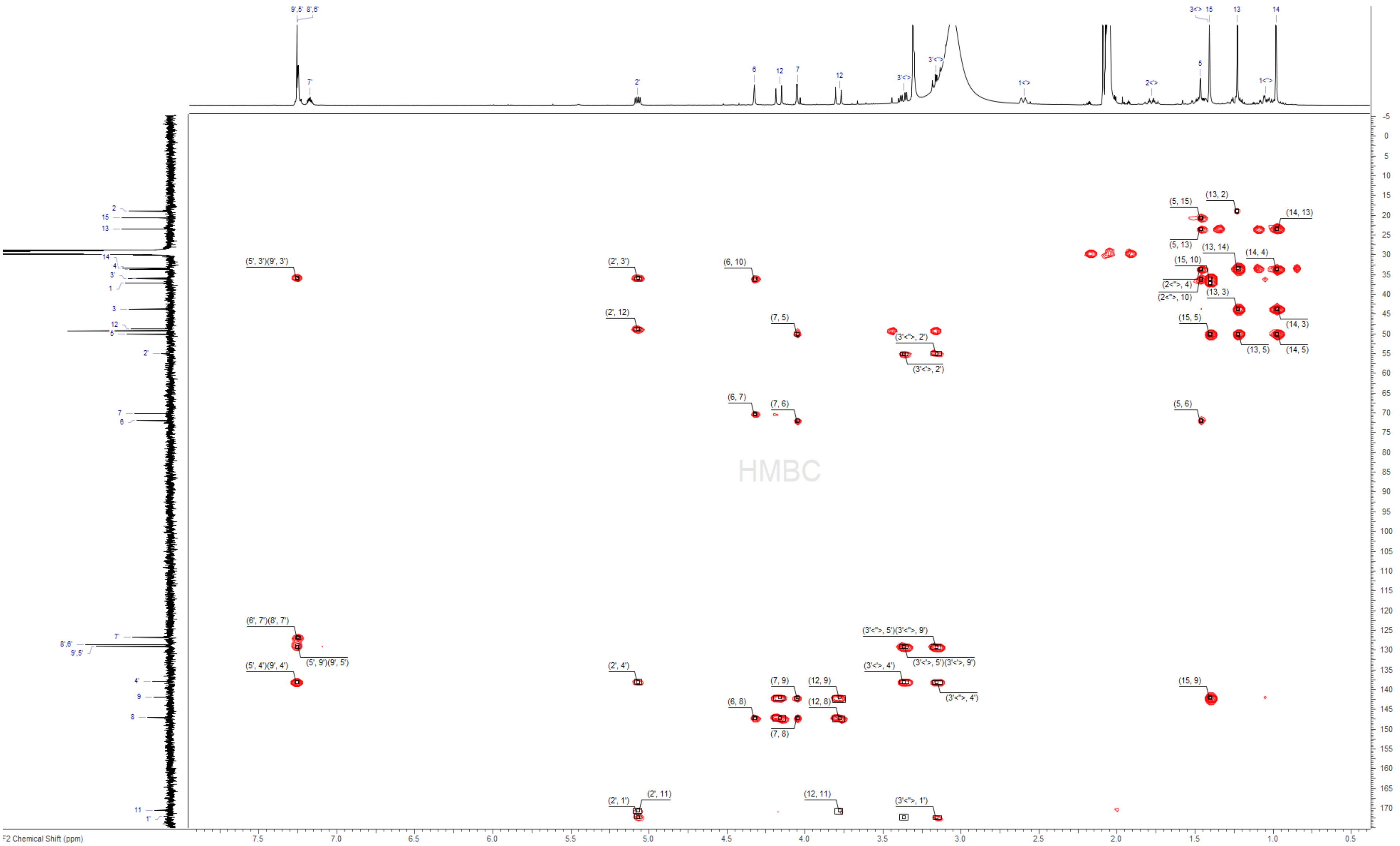

Figure S37 HMBC NMR spectrum (500 MHz, acetone-d_6) of **6**.

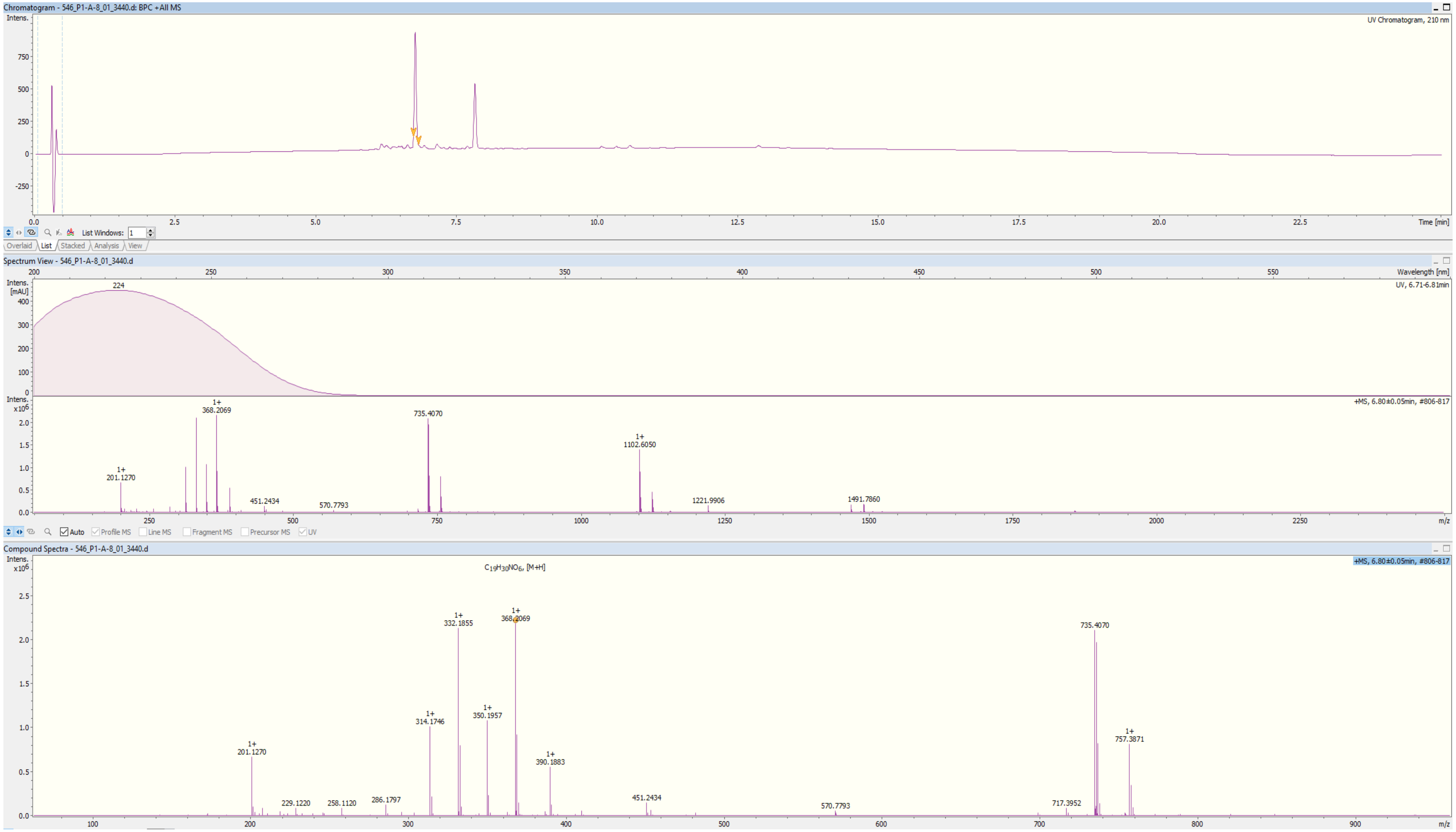

Figure S38 HPLC-HRESIMS data of **7**.

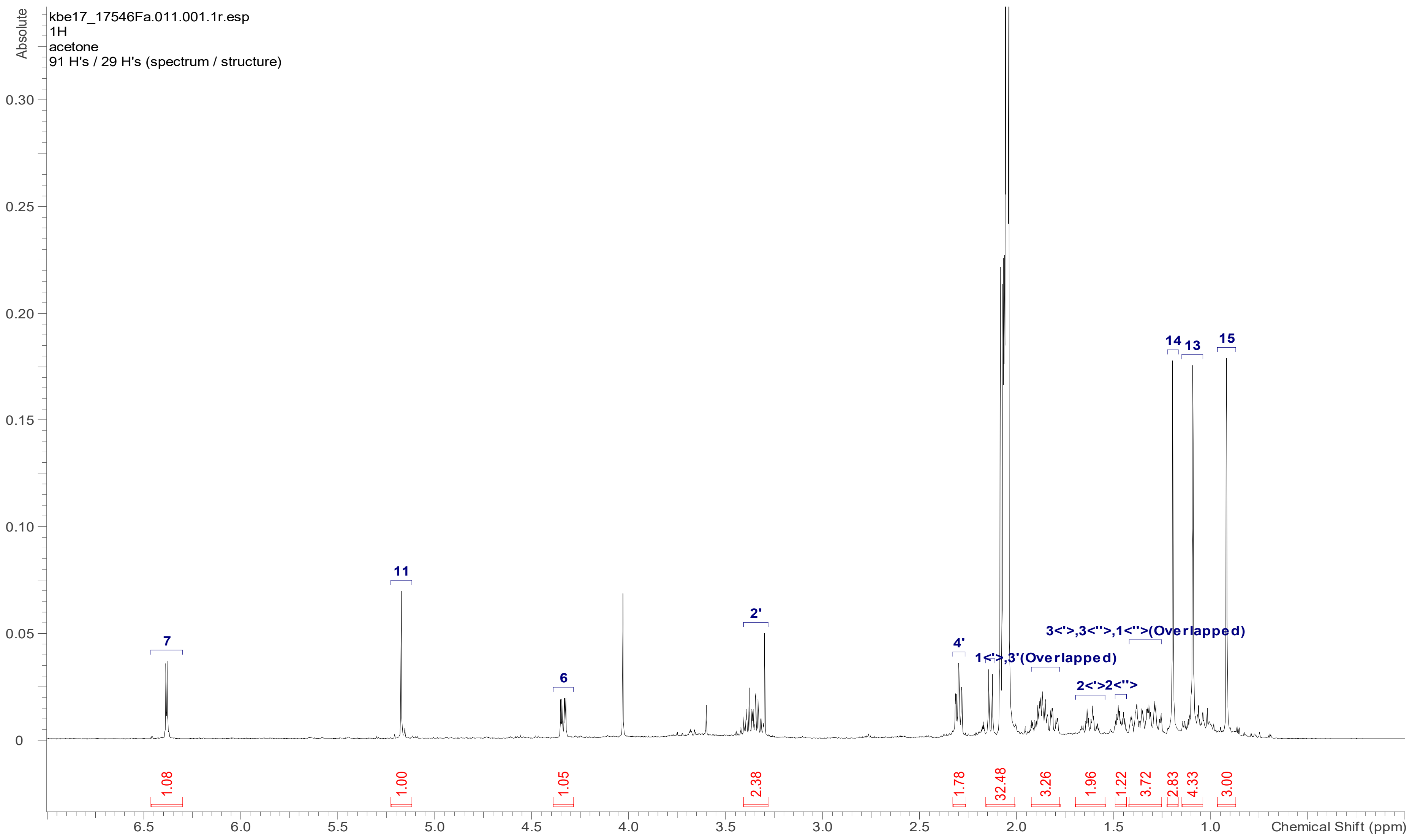

Figure S39 ^{1}H NMR spectrum (500 MHz, acetone-d_6) of **7**.

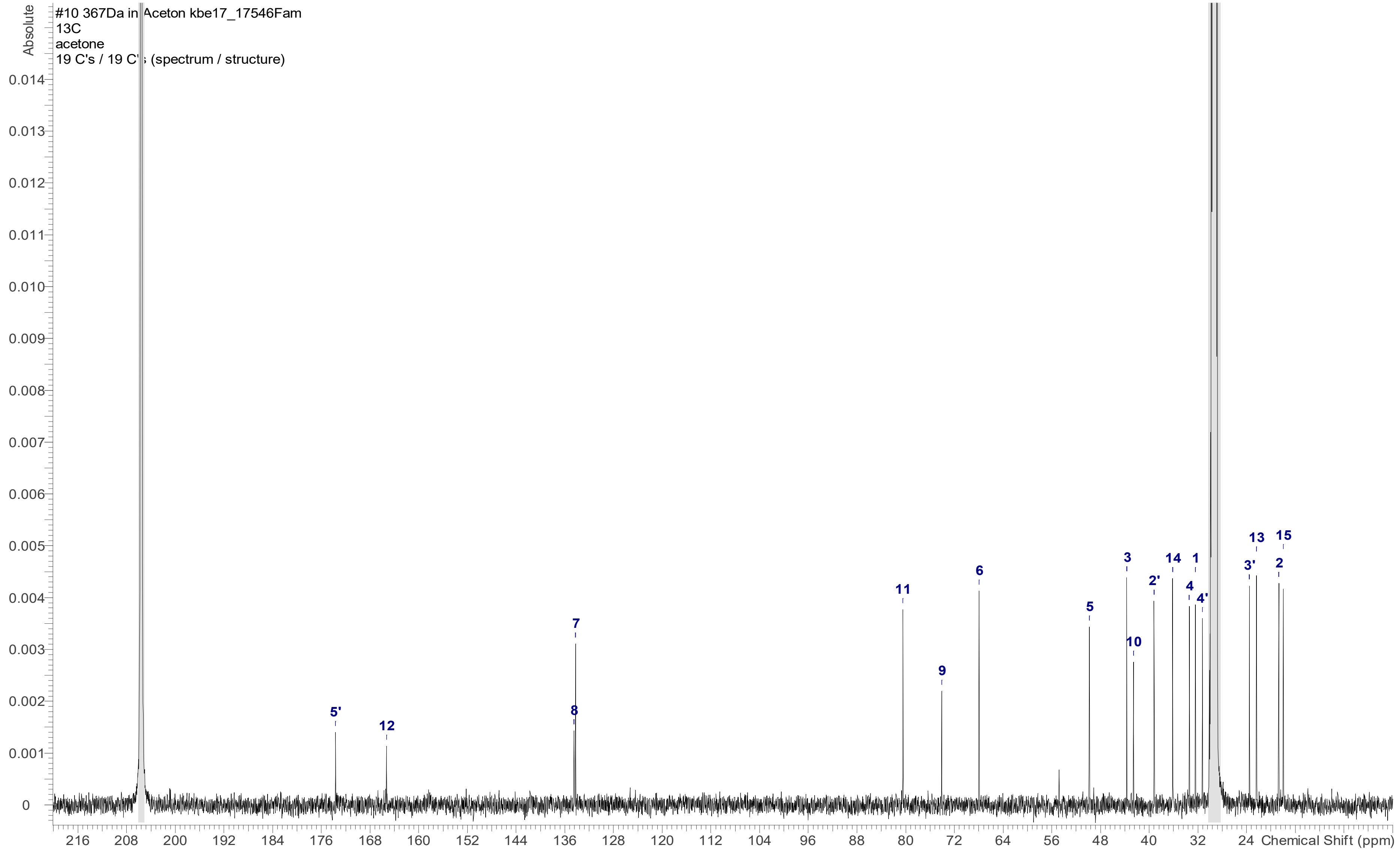

Figure S40 ^{13}C NMR spectrum (125 MHz, acetone-d_6) of **7**.

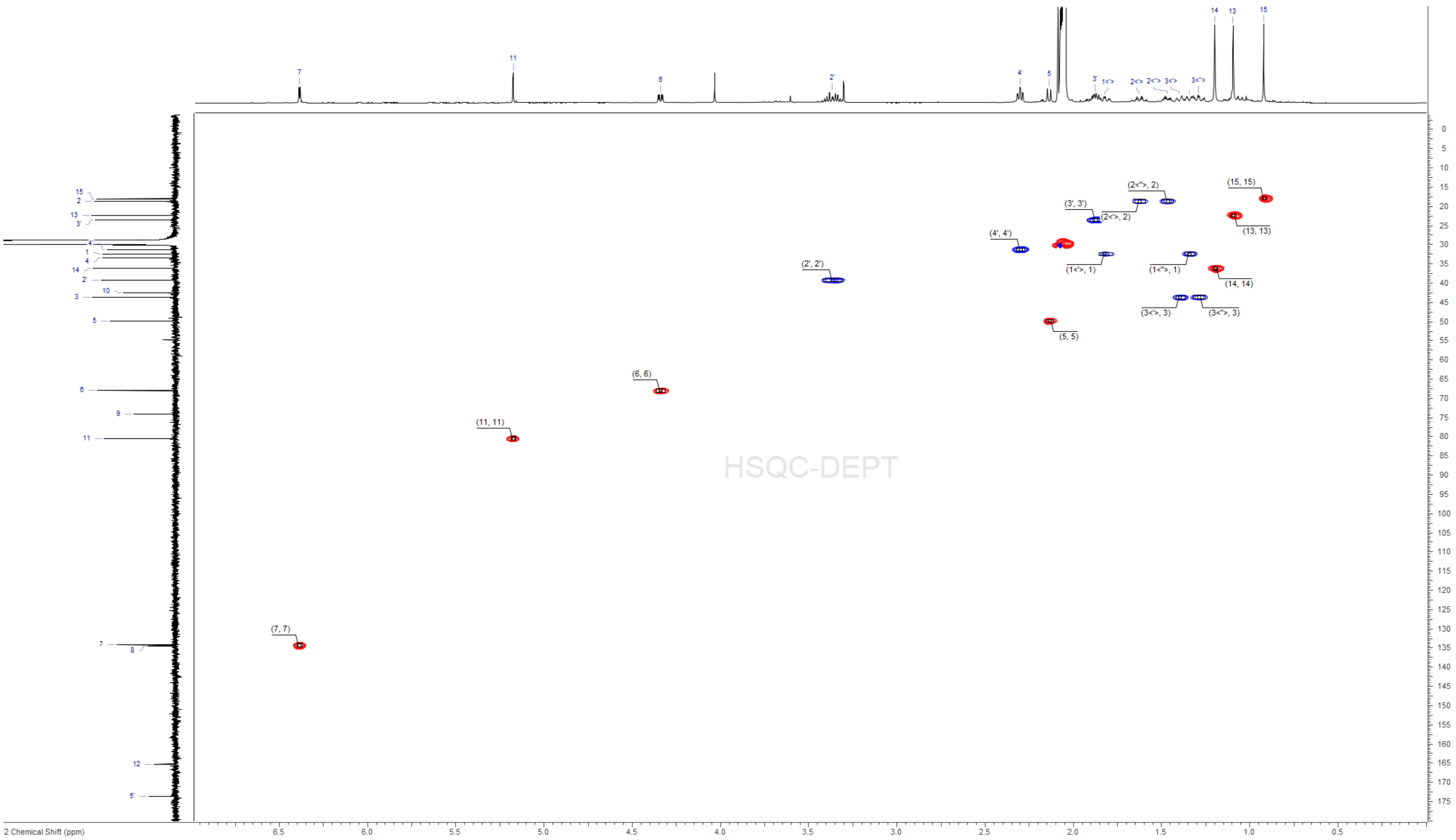

Figure S41 HSQC NMR spectrum (500 MHz, acetone-d_6) of **7**.

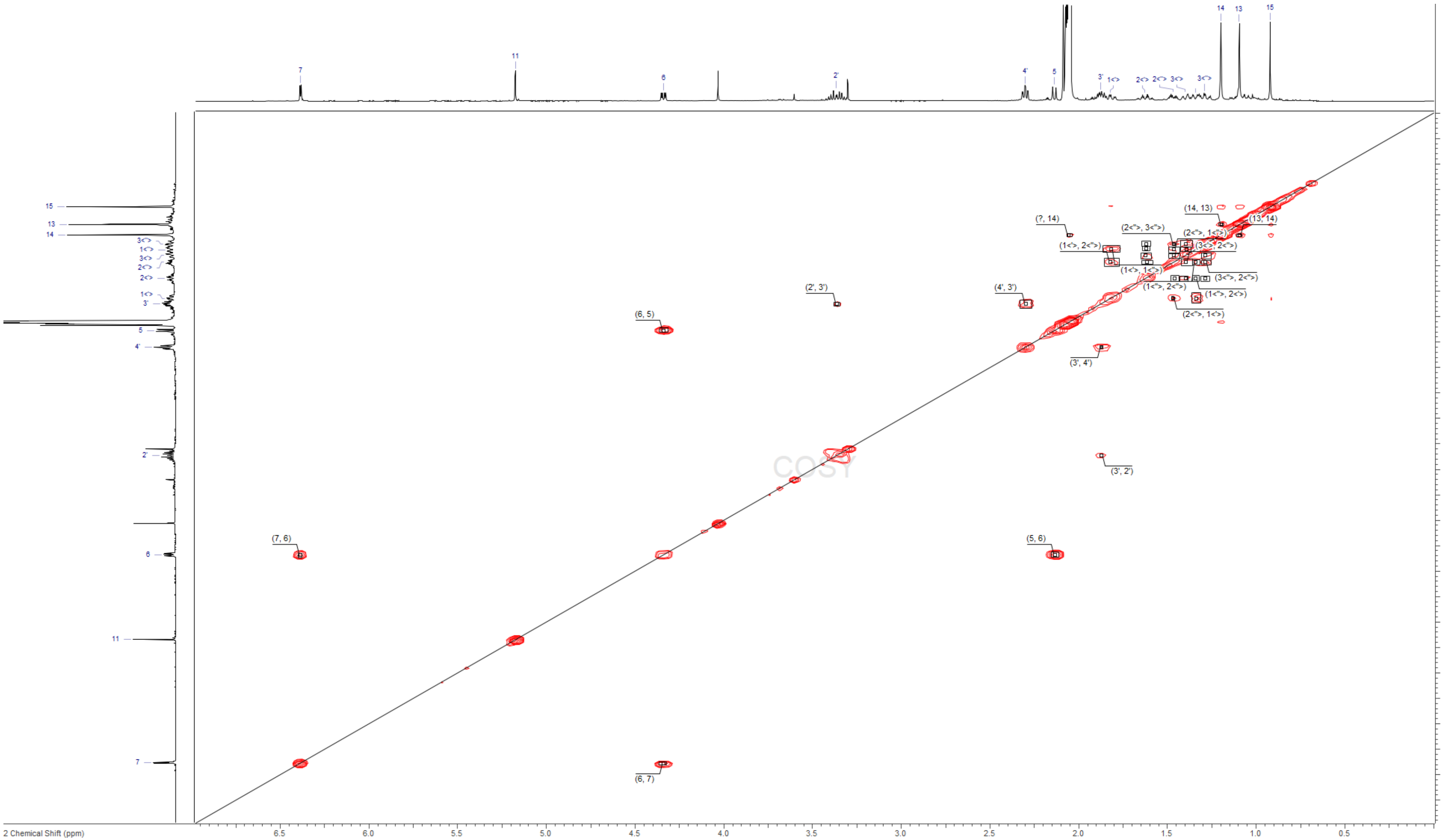

Figure S42 COSY NMR spectrum (500 MHz, acetone-d_6) of **7**.

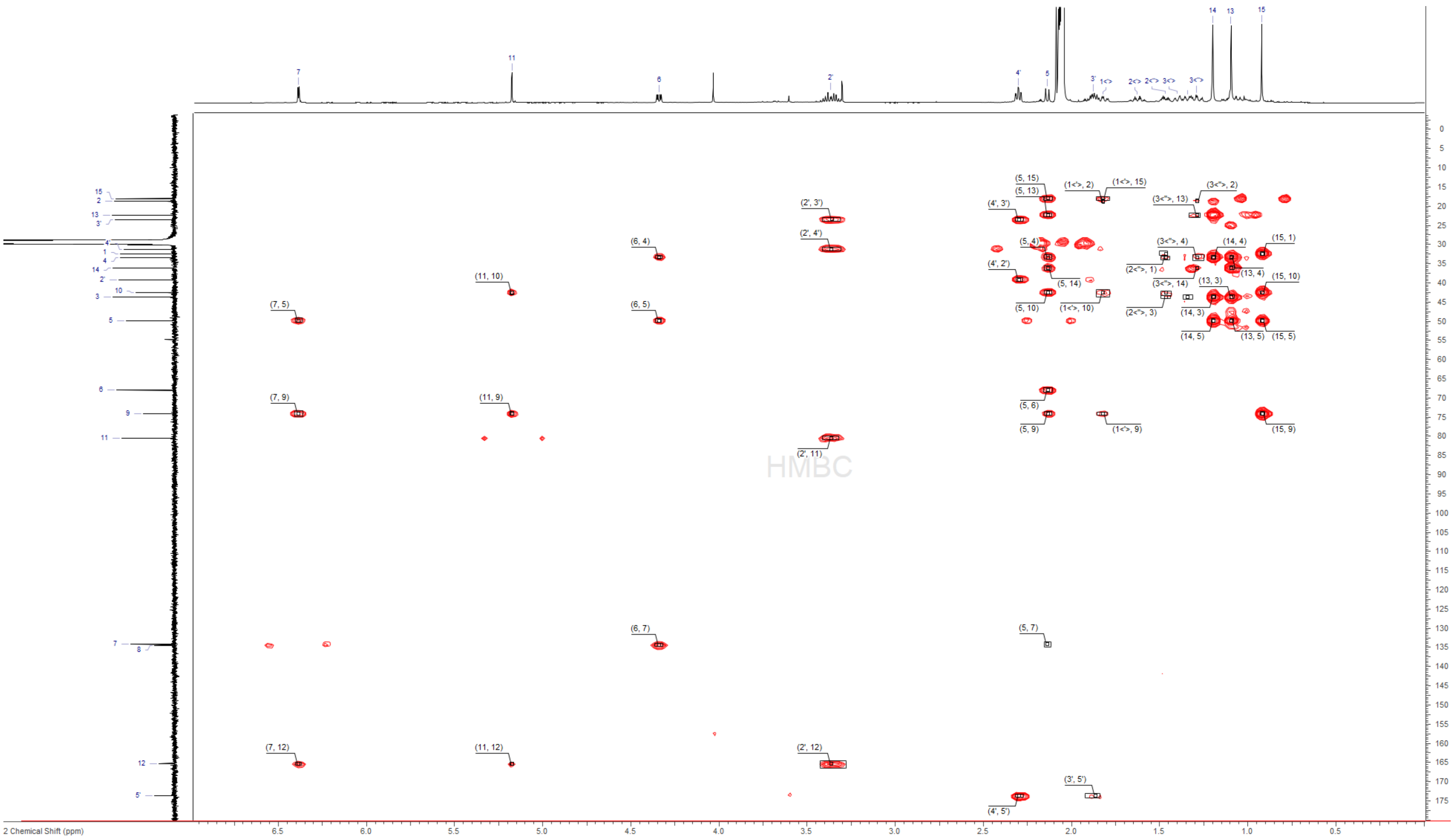

Figure S43 HMBC NMR spectrum (500 MHz, acetone-d_6) of **7**.

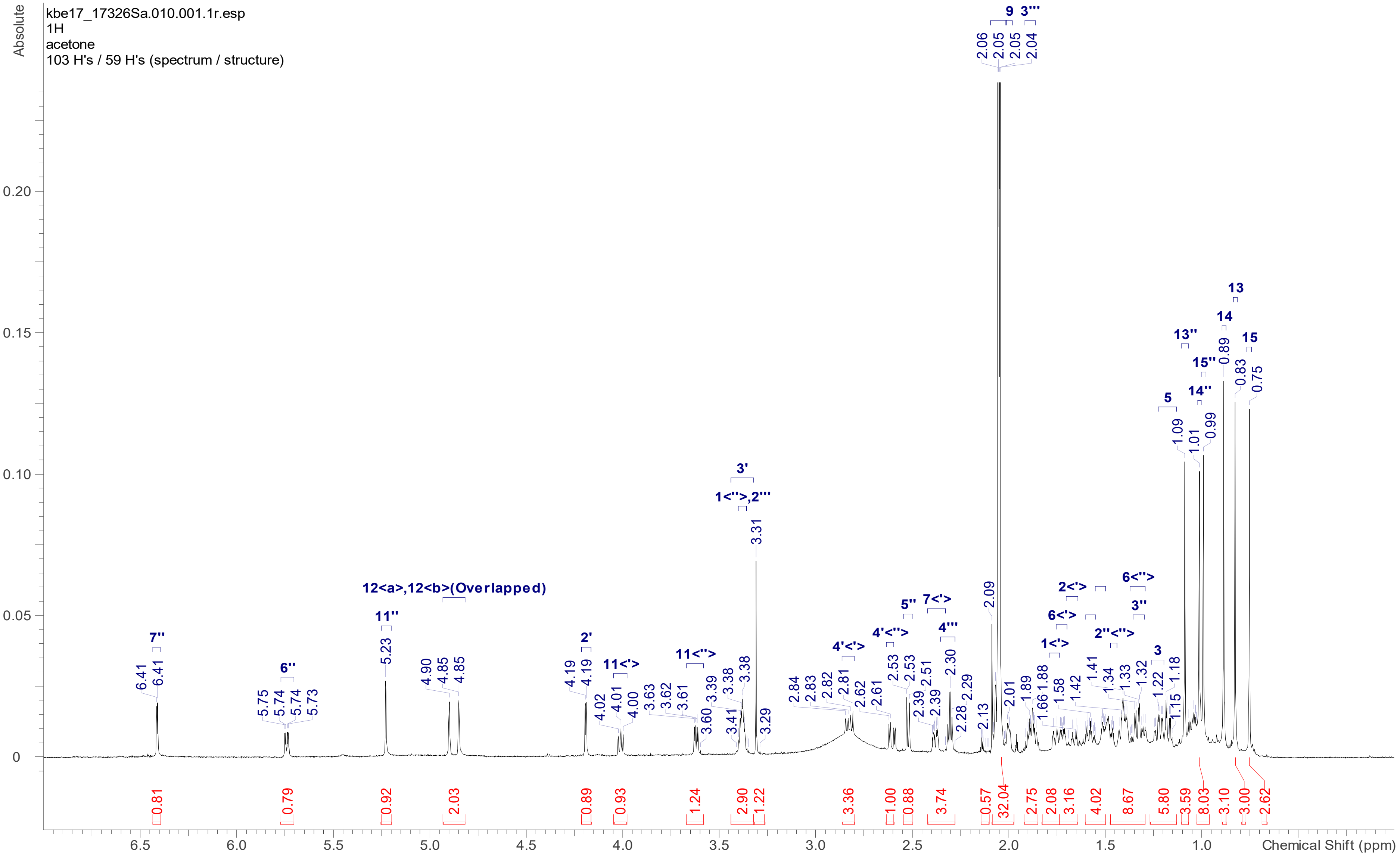

Figure S44 ^{1}H NMR spectrum (700 MHz, acetone-d_6) of **8**.

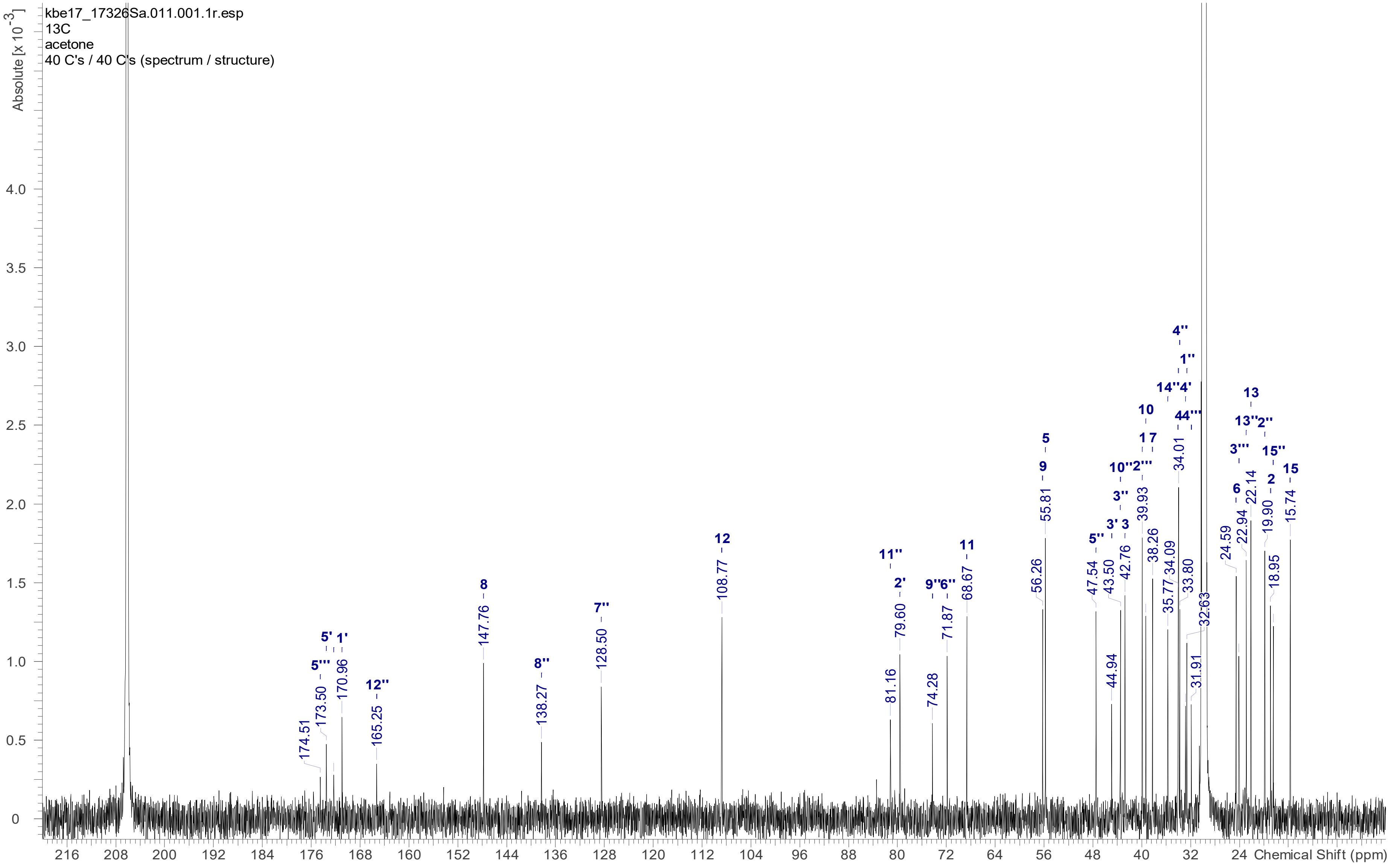

Figure S45 ^{13}C NMR spectrum (175 MHz, acetone-d_6) of **8**.

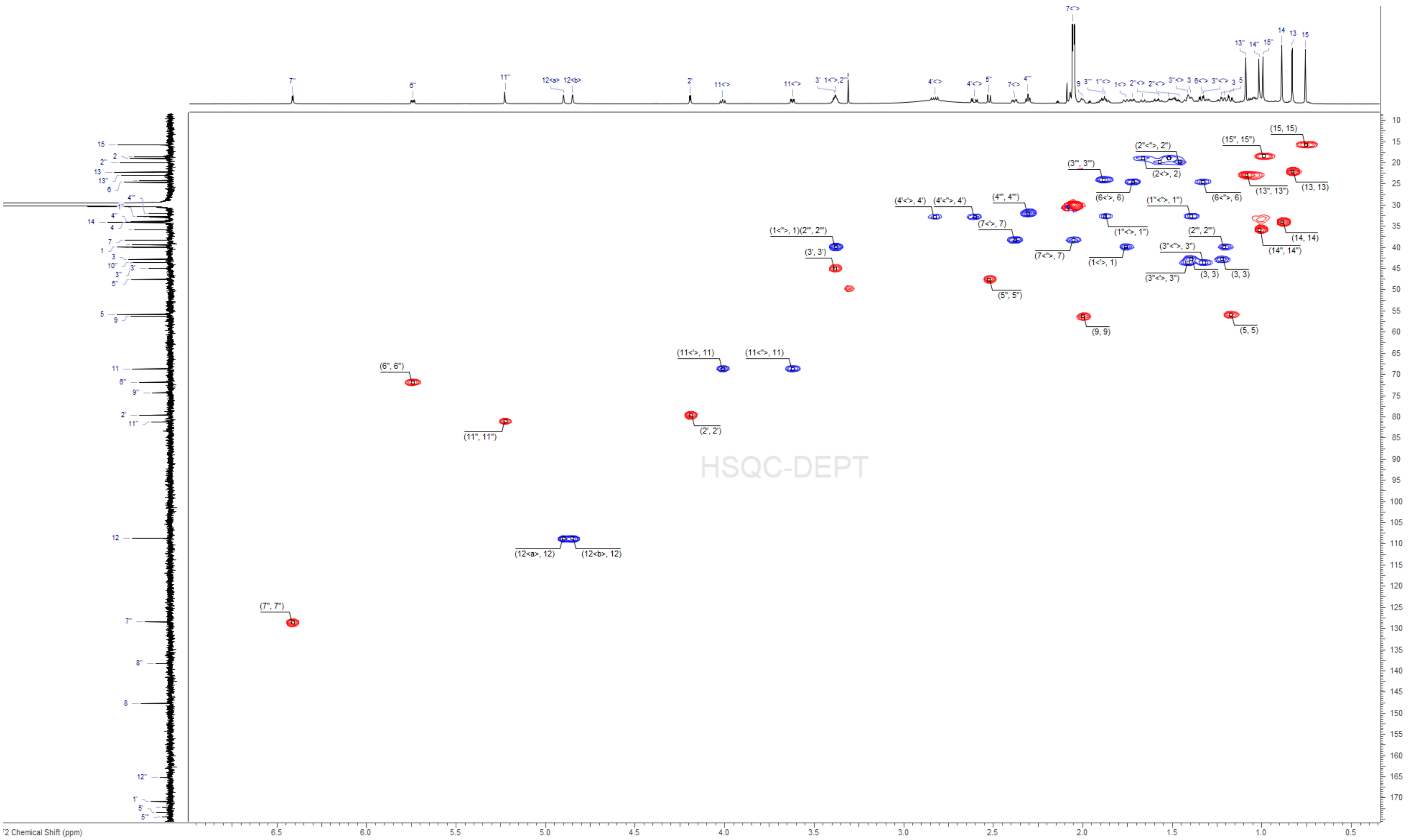

Figure S46 HSQC NMR spectrum (700 MHz, acetone-d_6) of **8**.

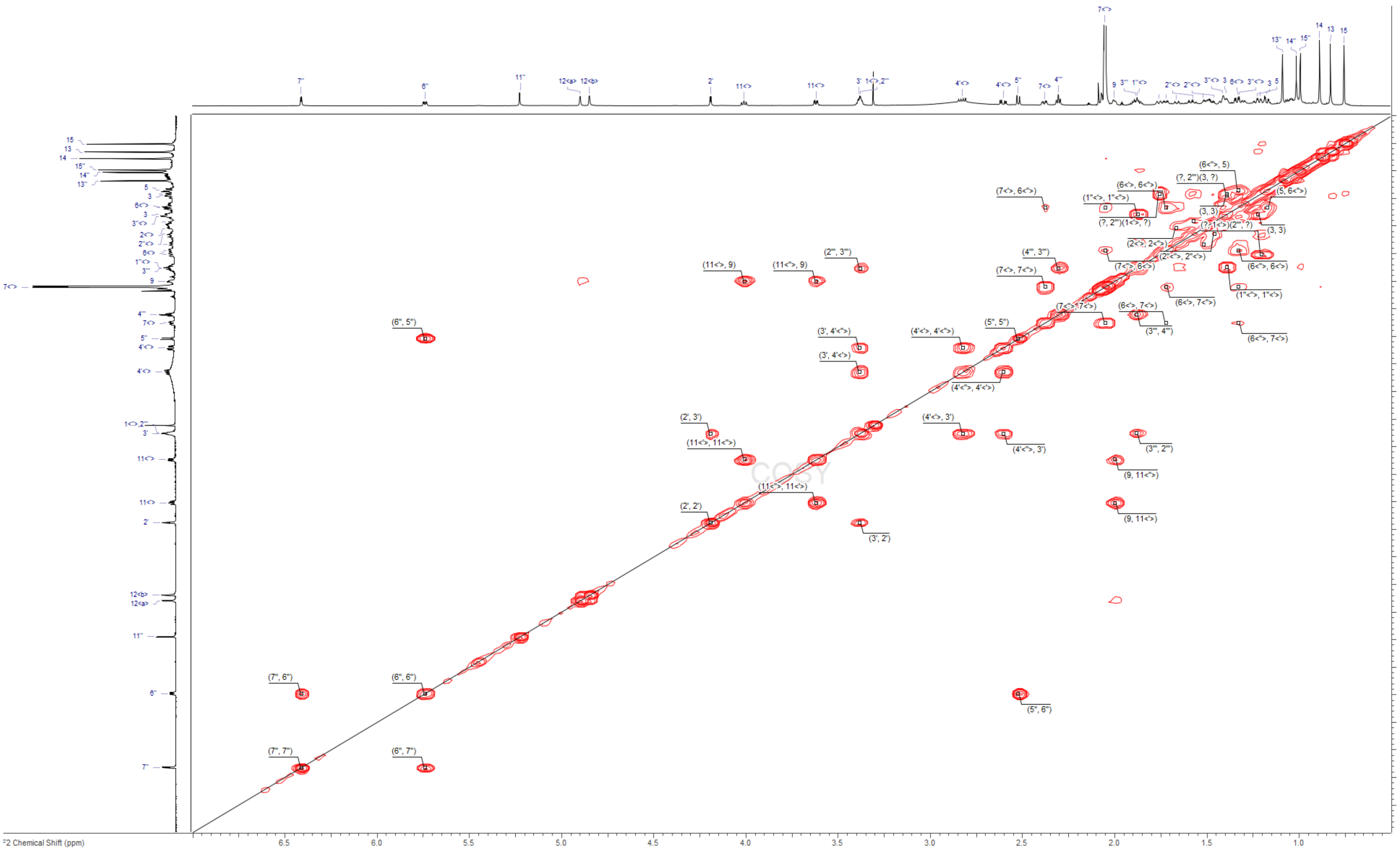

Figure S47 COSY NMR spectrum (700 MHz, acetone-d_6) of **8**.

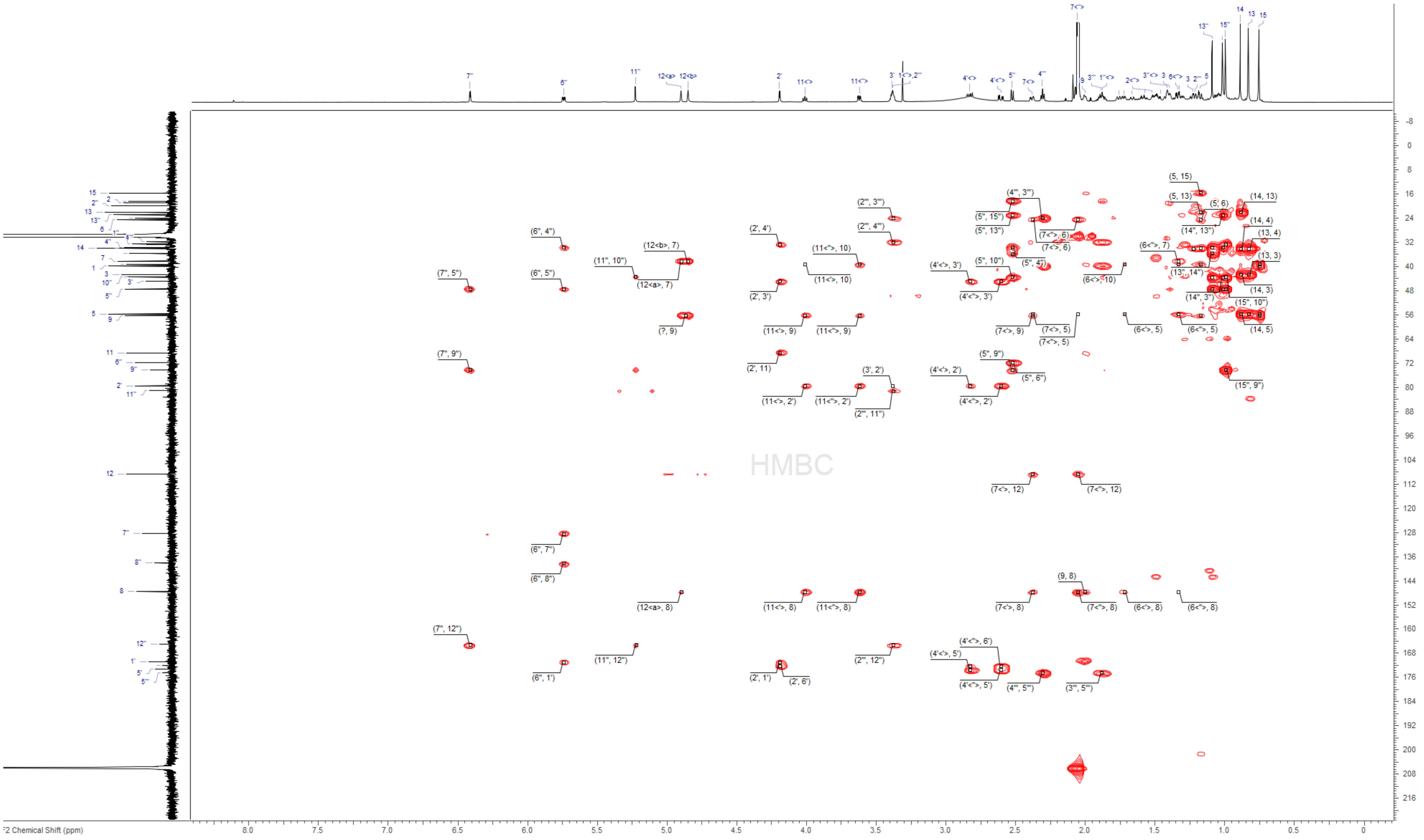

Figure S48 HMBC NMR spectrum (700 MHz, acetone-d_6) of **8**.

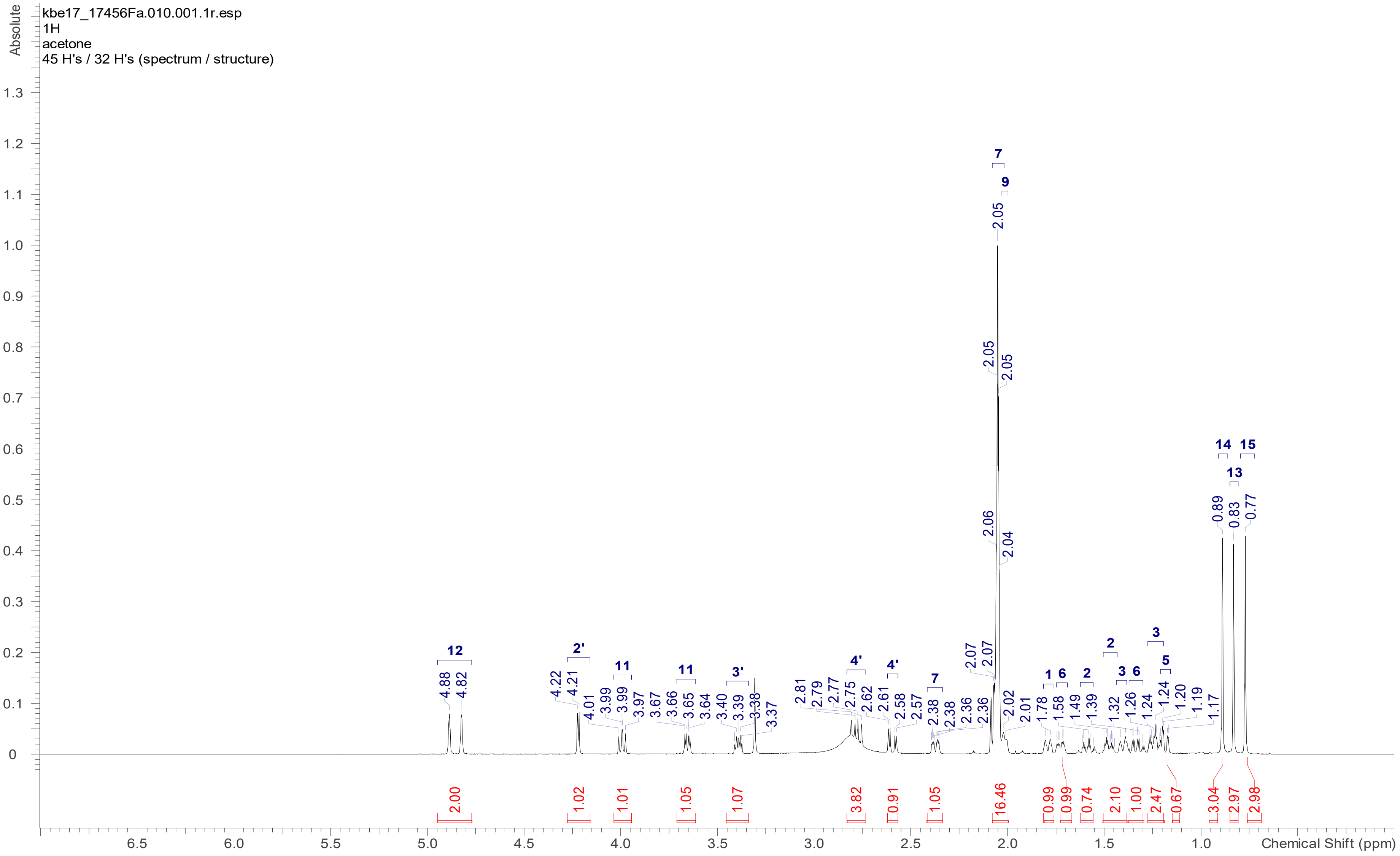

Figure S49 ^{1}H NMR spectrum (500 MHz, acetone-d_6) of cryptoporic acid H (**12**).

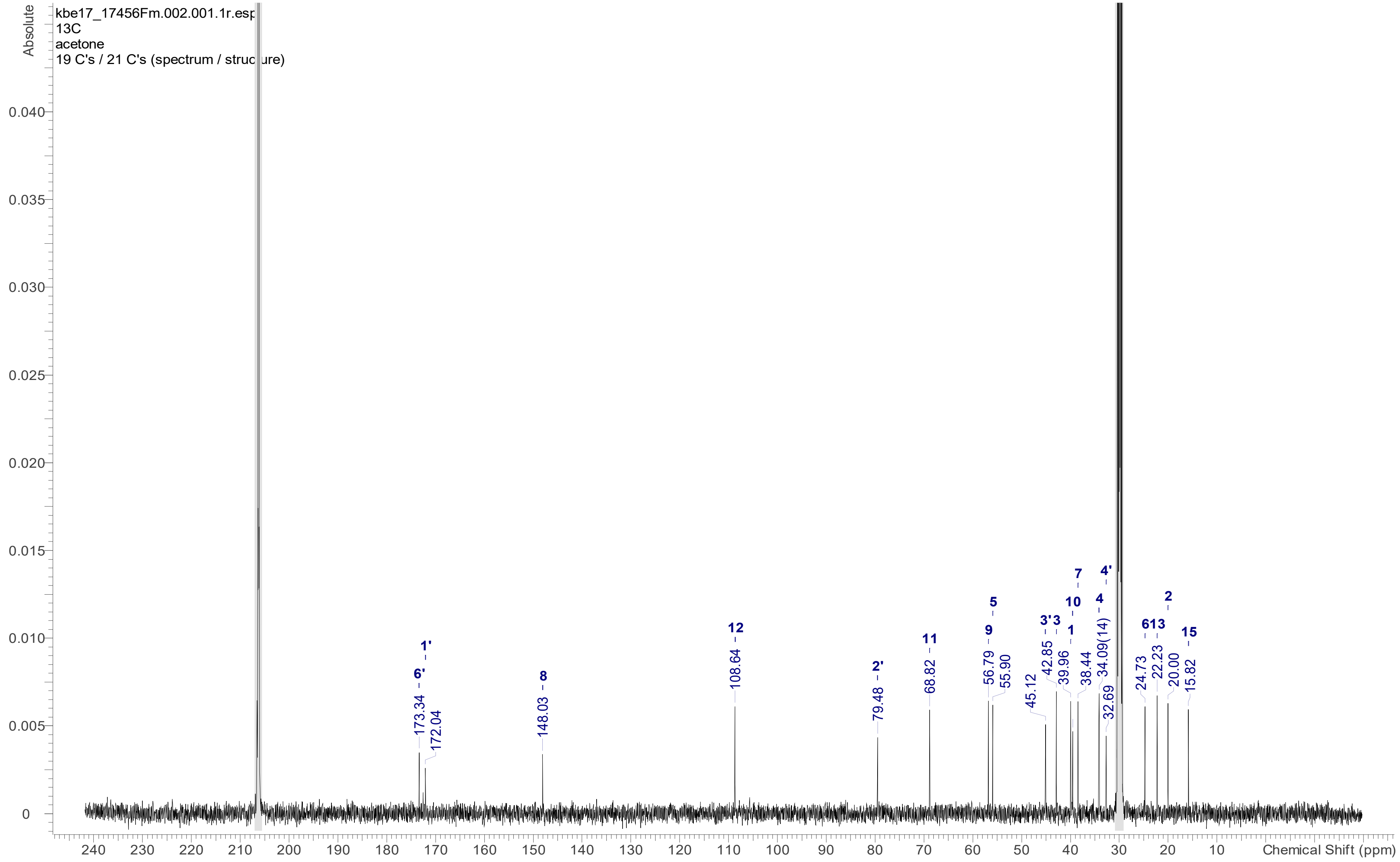

Figure S50 ^{13}C NMR spectrum (125 MHz, acetone-d_6) of cryptoporic acid H (**12**).

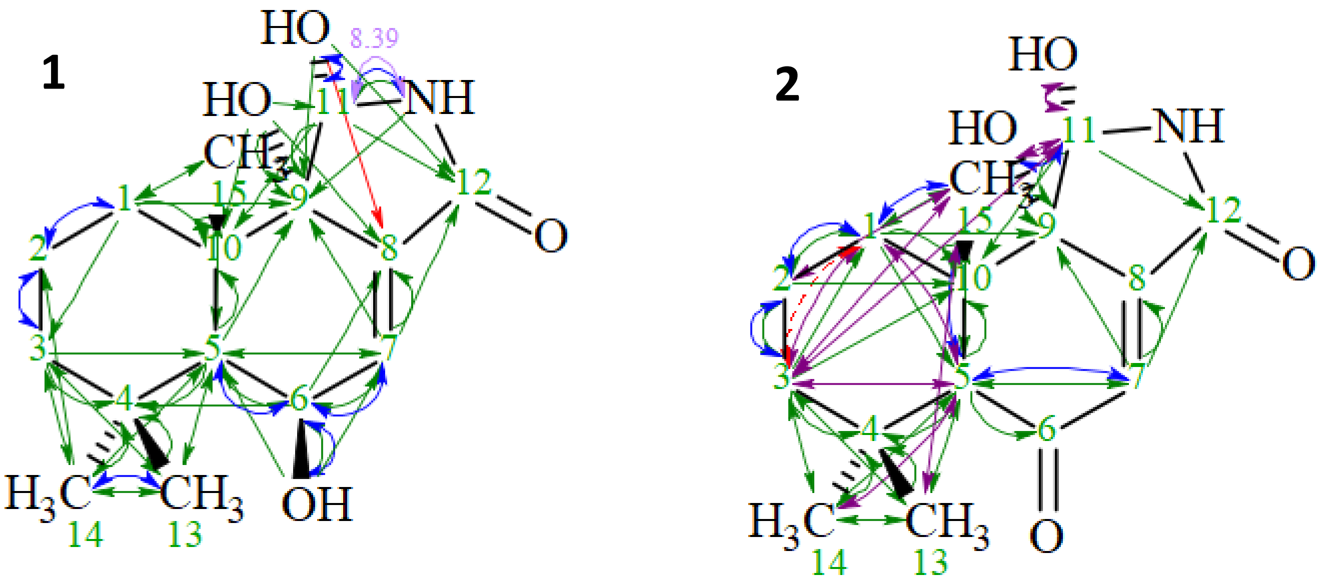

Figure S51 COSY (blue arrows), HMBC (green arrows) and ROESY (purple arrows) correlations for **1** and **2**.

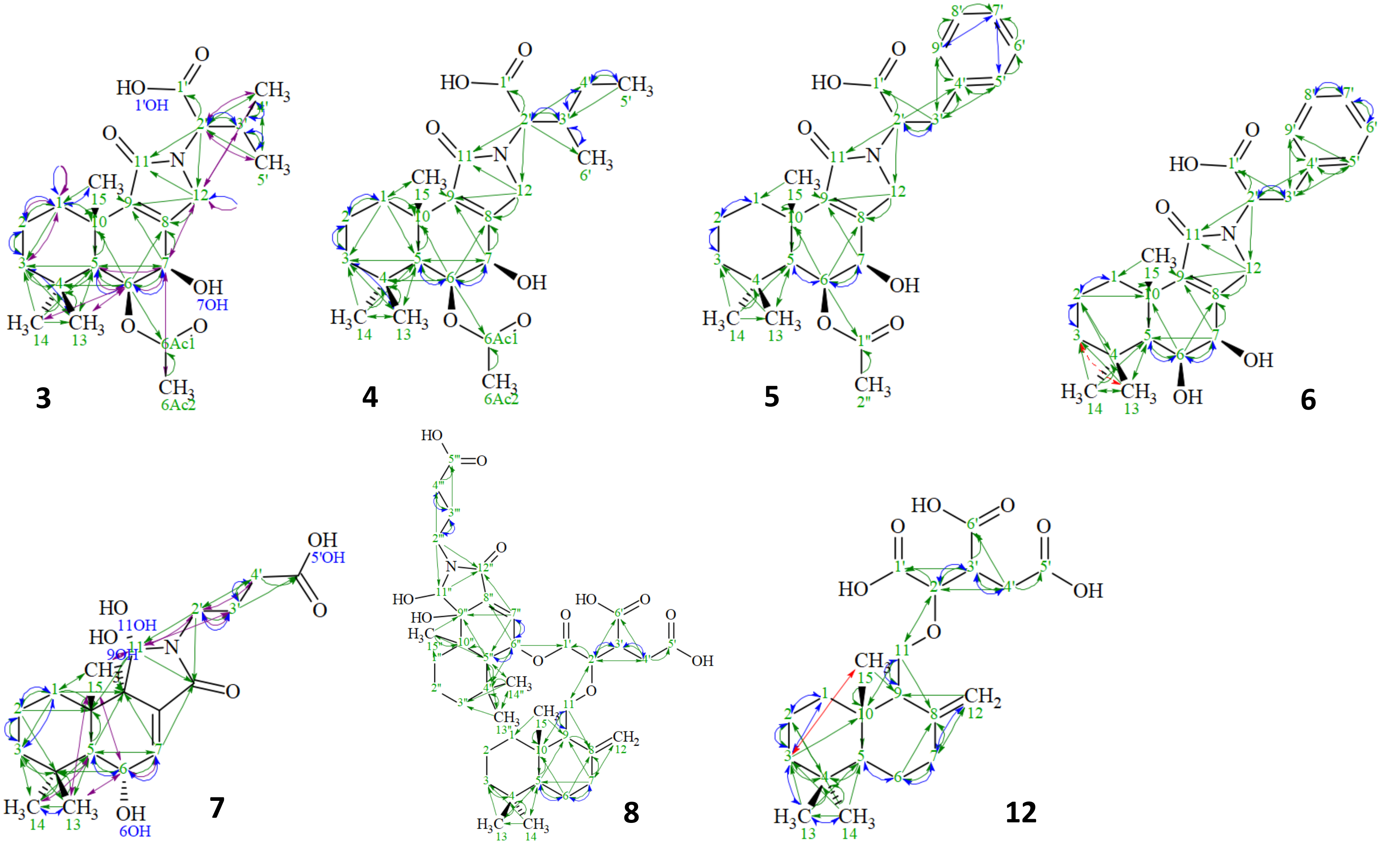

Figure S52 COSY (blue arrows), HMBC (green arrows) and ROESY (purple arrows) correlations for **3**–**8** & **12**.

Table S1. Hydrogen-bond geometry (Å, º) for **1**.

**D—H···A**	**D—H**	**H···A**	**D···A**	**D—H···A**
O6—H6···O12^{i}	0.87	2	2.848 (2)	164
O9—H9···O12^{i}	0.88	1.92	2.759 (2)	160
O11—H11···O9	0.91	1.91	2.500 (2)	120
O11—H11···O12^{ii}	0.91	2.24	2.937 (3)	133
N1—H1···O11^{iii}	0.9	2.08	2.956 (2)	165
O1W—H1W···O6^{iv}	1.02	1.71	2.712 (7)	166
O2W—H1W···O6^{iv}	1.17	1.71	2.76 (4)	145
O1W—H2W···O11	0.81	2.11	2.891 (7)	162
O2W—H2W···O11	0.92	2.11	2.71 (3)	122

Symmetry codes: (i) -x+1, y-1/2, -z+1; (ii) x, y-1, z; (iii) -x, y+1/2, -z+1; (iv) x-1, y-1, z.

Table S2. Geometric parameters (Å, º) for **1**.

C1—C2	1.527 (3)	O9—H9	0.8789
C1—C10	1.534 (3)	C9—C10	1.554 (3)
C9—C11	1.575 (3)	C10—C15	1.543 (3)
C2—C3	1.530 (3)	O11—C11	1.401 (3)
O11—H11	0.9141	C11—N1	1.464 (3)
C3—C4	1.538 (4)	O12—C12	1.245 (3)
C12—N1	1.336 (3)	C4—C13	1.534 (3)
C4—C14	1.536 (3)	C4—C5	1.563 (3)
C5—C6	1.552 (3)	C5—C10	1.568 (3)
O6—C6	1.443 (3)	N1—H1	0.8974
O6—H6	0.8683	C6—C7	1.509 (3)
C7—C8	1.330 (3)	O1W—O2W	0.87 (4)
C8—C12	1.481 (3)	O1W—H2W	0.8109
C8—C9	1.494 (3)	O2W—H1W	1.171
O9—C9	1.432 (3)	O2W—H2W	0.9232
O1W—H1W	1.0192		

Table S3 Result of a quantification assay

Compound code	Batch1			Batch2			Batch3			Batch4			Average	
	count	total	%	count	total	%	count	total	%	count	total	%	Average%	SD
**1**	48	65	73.85	75	115	65.22	48	61	78.69	59	109	54.13	68.0	9.3
**2**	45	87	51.72	22	64	34.38	25	74	33.78	26	79	32.91	38.2	7.8
**3**	12	65	18.46	13	75	17.33	16	96	16.67	18	83	21.69	18.5	1.9
**4**	35	84	41.67	23	97	23.71	36	87	41.38	18	99	18.18	31.2	10.5
**5**	23	90	25.56	21	98	21.43	16	87	18.39	22	109	20.18	21.4	2.6
**6**	18	95	18.95	24	98	24.49	23	108	21.30	12	78	15.38	20.0	3.3
**7**	25	75	33.33	32	154	20.78	12	89	13.48	21	108	19.44	21.8	7.2
**8**	36	95	37.89	32	115	27.83	23	151	15.23	24	87	27.59	27.1	8.0
**9**	10	95	10.53	12	87	13.79	15	105	14.29	12	98	12.24	12.7	1.5
**10**	8	78	10.26	12	102	11.76	18	98	18.37	11	107	10.28	12.7	3.3
**11**	48	75	64.00	38	67	56.72	42	69	60.87	47	84	55.95	59.4	3.3
Positive control	56	87	64.37	60	101	59.41	75	89	84.27	62	85	72.94	70.2	9.4
Blank	5	87	5.75	12	153	7.84	7	98	7.14	11	145	7.59	7.1	0.8
13	25	75	33.33	18	98	18.37	12	105	11.43	13	87	14.94	19.5	8.3
14	49	75	65.33	52	105	49.52	46	87	52.87	56	94	59.57	56.8	6.1
15	12	103	11.65	35	87	40.23	12	104	11.54	15	98	15.31	19.7	12.0
16	13	97	13.40	35	75	46.67	24	109	22.02	38	76	50.00	33.0	15.7
17	23	109	21.10	24	97	24.74	9	99	9.09	14	107	13.08	17.0	6.2
18	39	86	45.35	28	96	29.17	46	104	44.23	33	89	37.08	39.0	6.5
Positive control	65	87	74.71	43	75	57.33	68	96	70.83	87	104	83.65	71.6	9.5
Blank	9	102	8.82	5	98	5.10	12	105	11.43	6	98	6.12	7.9	2.5

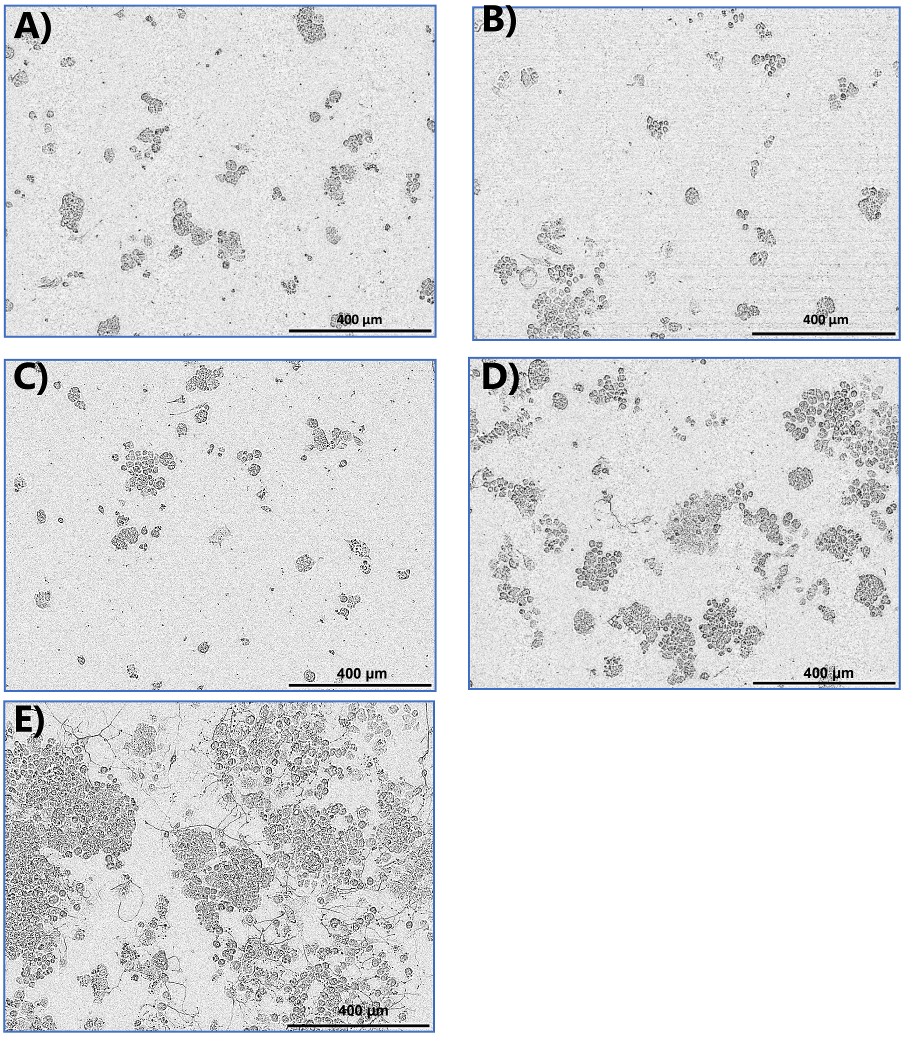

Figure S53 The image of PC12 cells after incubation with supernatant from astrocyte cells with various supplements at 5 days; A) Blank, B) Blank + NGF, C) compound **1**, D) compound **1**+NGF, G) NGF

Table S4 The gradients, retention times and yields for the individual compounds

**Compound**	**Yield(mg)**	**Retention time (min)**	**Gradient (Solvent B)**				
**1**	5.8	12	5%<br>(3 min)	5-50%<br>(45 min)	50-100%<br>(10 min)	100%<br>(10 min)	
	70	10.5	0%<br>(10 min)	0-15%<br>(10 min)	15-100%<br>(30 min)	100%<br>(20 min)	
**2**	2.2	15.5	5%<br>(3 min)	5-50%<br>(45 min)	50-100%<br>(10 min)	100%<br>(10 min)	
	3.7	18	10%<br>(10 min)	10-40%<br>(30 min)	40-100%<br>(10 min)	100%<br>(5 min)	
**3**	7.5	20.2	20%<br>(5 min)	20-35%<br>(10 min)	35-45%<br>(40 min)	45-100%<br>(5 min)	100%<br>(5 min)
**4**	2.4	25.5	20%<br>(5 min)	20-40%<br>(10 min)	40-50%<br>(40 min)	50-100%<br>(5 min)	100%<br>(5 min)
**5**	6	28.5	20%<br>(5 min)	20-35%<br>(10 min)	35-45%<br>(50 min)	45-100%<br>(5 min)	100%<br>(5 min)
**6**	2.4	35.2	20%<br>(10 min)	20-25%<br>(10 min)	25-35%<br>(40 min)	35-100%<br>(10 min)	
**7**	8	38.2	20%<br>(10 min)	20-30%<br>(10 min)	30-40%<br>(30 min)	40-100%<br>(10 min)	100%<br>(5 min)
**8**	1.5	35.4	5%<br>(3 min)	5-50%<br>(45 min)	50-100%<br>(10 min)	100%<br>(10 min)	5%<br>(3 min)

Table S5 The following PCR primers were used for amplifying specific cDNA fragments

PCR primer	Sense	Antisense	Size (bp)
gapdh	5′-ACCACAGTCCATGCCATCAC-3′	5′-TCCACCACCCTGTTGCTGTA-3′	451
ngf	5′CCAAGGGAGCAGTTTCTATCCTG G-3′	5′GGCAGTTGTCAAGGGAATGCTGAAGT T-3′	189
bdnf	5′-TAACGGCGGCAGACAAAAAGA-3′	5′-GAAGTATTGCTTCAGTTGGCCT-3′	101

Method descriptions

HPLC-DAD/MS measurements were performed using an amaZon speed ETD (electron transfer dissociation) ion trap mass spectrometer (Bruker Daltonics) and measured in positive and negative ion modes simultaneously, with the HPLC system (column C18 Acquity UPLC BEH (Waters), solvent A: water (H_2O); solvent B: acetonitrile (ACN) supplemented with 0.1% formic acid (FA), gradient conditions: 5% B for 0.5 min, increasing to 100% B in 20 min, maintaining isocratic conditions at 100% B for 10 min, flow rate 0.6 mL/min, UV/Vis detection 200-600 nm), used.
HR-ESIMS (high-resolution electrospray ionization mass spectrometry) data were recorded on a MaXis ESI-TOF (electrospray ionization-time of flight) mass spectrometer (Bruker Daltonics, Bremen, Germany) coupled to an Agilent 1260 series HPLC-UV system and equipped with a C18 Acquity UPLC BEH (ultraperformance liquid chromatography) (ethylene bridged hybrid) (Waters) column; DAD-UV detection at 200–600 nm; solvent A (H_2O) and solvent B (ACN) supplemented with 0.1% FA as a modifier; flowrate 0.6 mL/min, 40 C, gradient elution system with the initial condition 5% B for 0.5 min, increasing to 100% B in 19.5 min and holding at 100% B for 5 min.

1D and 2D NMR spectra were measured on a Bruker 700 MHz Avance III spectrometer equipped with a 5 mm TCI cryoprobe (^{1}H: 700 MHz, ^{13}C: 175 MHz) and a Bruker Avance III 500 (^{1}H 500 MHz, ^{13}C 125 MHz) spectrometer. NMR data were referenced to selected chemical shifts of acetone-d_6 (^{1}H: 2.05 ppm, ^{13}C: 29.32 ppm) and CH_3OH-d_4 (^{1}H: 3.31 ppm, ^{13}C: 49.15 ppm), respectively. Optical rotations were measured using an Anton Paar MCP-150 Polarimeter (Graz, Austria) with 100 mm path length and sodium D line at 589 nm. The UV spectra were measured on a Shimadzu (Kyoto, Japan) UV/Vis 2450 spectrophotometer using methanol (Uvasol, Merck, Darmstadt, Germany) as a solvent. ECD (electronic circular dichroism) spectra were measured with a J-815 spectropolarimeter (Jasco, Pfungstadt, Germany) using methanol as a solvent.

For cell culture, Astrocytoma (1321N1, Sigma-Aldrich, acc. No. 86030402) cells were cultured in Gibco DMEM medium (Fisher Scientific, Inc., Waltham, MA, USA) containing 10% heat-inactivated FBS ((Capricorn™ Scientific GmbH, Ebsdorfergrund, Germany) Rat pheochromocytoma cells (PC-12) purchased from the European Collection of Authenticated Cell Cultures (ECACC) general collection were grown in Gibco™ RPMI-1640 (Fisher Scientific) medium containing 10% horse serum (Capricorn) and 5% heat-inactivated fetal bovine serum-FBS (Capricorn). The media were supplemented with penicillin (0.15 mM), streptomycin (86 µM), and glutamine (2 mM). The cells were incubated at 37 °C in a humidified environment of 7.5% CO_2 and 95% air and were routinely passaged every 3-4 days. Collagen type IV (Sigma C5533) was coated on 96 well plates and left for 6 hours or more before using the plates whenever seeding PC-12 cells.

The PC-12 cells were seeded at a density of 1×10^3 cells per well in growth medium in 96-well culture plates and incubated overnight. The supernatant of treatment between compounds and 1321N1 cells was transferred to treat with PC12 cells. Cells treated with nerve growth factor (50ng/mL) were used as a positive control. After 3 days, cell differentiation and neurite outgrowth were examined using an IncuCyte S3 live-cell analysis system (Sartorius, Göttingen, Germany). Six random fields were examined in each well. Neurite length was measured using IncuCyte NeuroTrack Software Module for 6 days. The number of cells differentiated i.e., axon-like protusions in the cells, defined as extensions longer than twice the cell body diameter, was recorded. Three independent experiments were conducted for each compound.

For real-time quantitative reverse transcriptase PCR, the total RNA was extracted from treatment of 1321N1 cells ($2\text{x}10^5$ cells) with selected compounds. For treatment, culture media was replaced by serum-reduced medium (Gibco RPMI with 1% FBS (Capricorn)) and cells were incubated for 24h. Then, media was replaced with media containing the drimanes dissolved in 0.5% DMSO. As control, serum-reduced medium supplemented with 0.5% DMSO was used. Cells were incubated for 48 h.

Total RNA was extracted using the NucleoSpinR RNA Plus kit (Macherey-Nagel GmbH& Co KG, Düren, Germany) followed by further purification (NucleoSpinR RNA Clean-up kit) according to the manufacturer's protocol. To determine the concentration of purified RNA the corresponding samples were measured using a DS-11+ spectrophotometer Nanodrop (DeNovix Inc., Wilmington, Delaware USA). First strand cDNA synthesis and subsequent real-time PCR was performed using SensiFast TM SYBR No-Rox One-Step Kit (Cat.No. BIO-72005 (Bioline)).

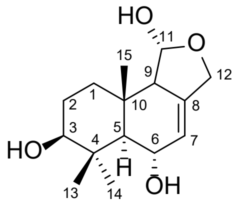

3β-hydroxy-9-dehydroxy-pereniporin A (13)

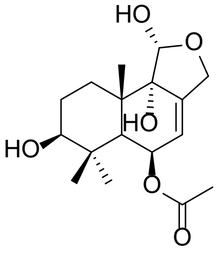

3β-hydroxy-6-*O*-acetylpereniporin A (14)

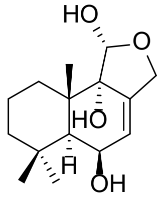

pereniporin A (15)

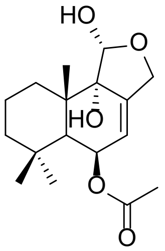

6-*O*-acetylpereniporin A (16)

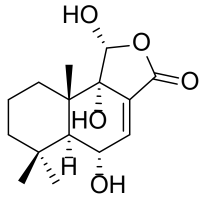

6α,9α,11α-trihydroxycinnamolide (17)

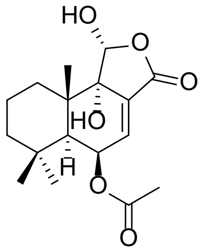

11α-hydroxycinnamosmolide (18)

Figure S54: Structures of metabolites **13** – **18** isolated from *Perenniporia centrali-africana* MUCL 56028.
